# From Allergen Molecules to Molecular Immunotherapy of Nut Allergy: A Hard Nut to Crack

**DOI:** 10.3389/fimmu.2021.742732

**Published:** 2021-09-23

**Authors:** Verena Fuhrmann, Huey-Jy Huang, Aysegul Akarsu, Igor Shilovskiy, Olga Elisyutina, Musa Khaitov, Marianne van Hage, Birgit Linhart, Margarete Focke-Tejkl, Rudolf Valenta, Bulent Enis Sekerel

**Affiliations:** ^1^ Division of Immunopathology, Department of Pathophysiology and Allergy Research, Center for Pathophysiology, Infectiology and Immunology, Medical University of Vienna, Vienna, Austria; ^2^ Division of Allergy and Asthma, Department of Pediatrics, Hacettepe University Faculty of Medicine, Ankara, Turkey; ^3^ Laboratory for Molecular Allergology, National Research Center (NRC) Institute of Immunology Federal Medical-Biological Agency (FMBA) of Russia, Moscow, Russia; ^4^ Pirogov Russian National Research Medical University, Moscow, Russia; ^5^ Department of Medicine Solna, Division of Immunology and Allergy, Karolinska Institutet and Karolinska University, Hospital, Stockholm, Sweden; ^6^ Karl Landsteiner University of Health Sciences, Krems, Austria; ^7^ Laboratory of Immunopathology, Department of Clinical Immunology and Allergology, Sechenov First Moscow State Medical University, Moscow, Russia

**Keywords:** allergen molecules, component, food allergy, immunotherapy, molecular allergy diagnosis, peanut, tree nut

## Abstract

Peanuts and tree nuts are two of the most common elicitors of immunoglobulin E (IgE)-mediated food allergy. Nut allergy is frequently associated with systemic reactions and can lead to potentially life-threatening respiratory and circulatory symptoms. Furthermore, nut allergy usually persists throughout life. Whether sensitized patients exhibit severe and life-threatening reactions (e.g., anaphylaxis), mild and/or local reactions (e.g., pollen-food allergy syndrome) or no relevant symptoms depends much on IgE recognition of digestion-resistant class I food allergens, IgE cross-reactivity of class II food allergens with respiratory allergens and clinically not relevant plant-derived carbohydrate epitopes, respectively. Accordingly, molecular allergy diagnosis based on the measurement of allergen-specific IgE levels to allergen molecules provides important information in addition to provocation testing in the diagnosis of food allergy. Molecular allergy diagnosis helps identifying the genuinely sensitizing nuts, it determines IgE sensitization to class I and II food allergen molecules and hence provides a basis for personalized forms of treatment such as precise prescription of diet and allergen-specific immunotherapy (AIT). Currently available forms of nut-specific AIT are based only on allergen extracts, have been mainly developed for peanut but not for other nuts and, unlike AIT for respiratory allergies which utilize often subcutaneous administration, are given preferentially by the oral route. Here we review prevalence of allergy to peanut and tree nuts in different populations of the world, summarize knowledge regarding the involved nut allergen molecules and current AIT approaches for nut allergy. We argue that nut-specific AIT may benefit from molecular subcutaneous AIT (SCIT) approaches but identify also possible hurdles for such an approach and explain why molecular SCIT may be a hard nut to crack.

## 1 Introduction

Nuts are nutrient-dense foods that receive increasing attention due to reports regarding their possible health-promoting properties and their pleasant taste (1, 2). At the same time, tree nuts and peanuts are among the most common elicitors of anaphylaxis, a severe, potentially life-threatening hypersensitivity reaction mediated by allergen-specific IgE antibody-induced mast cell and basophil activation (3–6). The possibility of accidental nut ingestion and the associated fear of experiencing severe allergic reactions is particularly challenging for nut-allergic children and their parents and results in a considerable reduction in quality of life (7–10).

In allergology, a distinction is made between tree nuts and peanuts by defining a nut according to what is considered a nut in the culinary sense and less according to the botanical definition. Generally, “real” botanical nuts like the hazelnut, but also several seeds and drupes that grow on trees are considered tree nuts. Peanuts, which grow underground, are classified as legumes (11). Walnut, pistachio, pecan, hazelnut, almond, cashew, Brazil nut and macadamia are responsible for most allergic reactions to tree nuts and therefore included in this review under the umbrella of “tree nuts” (11) and the term “nut” used herein generally refers to peanuts and tree nuts unless otherwise specified.

**Figure 1:**
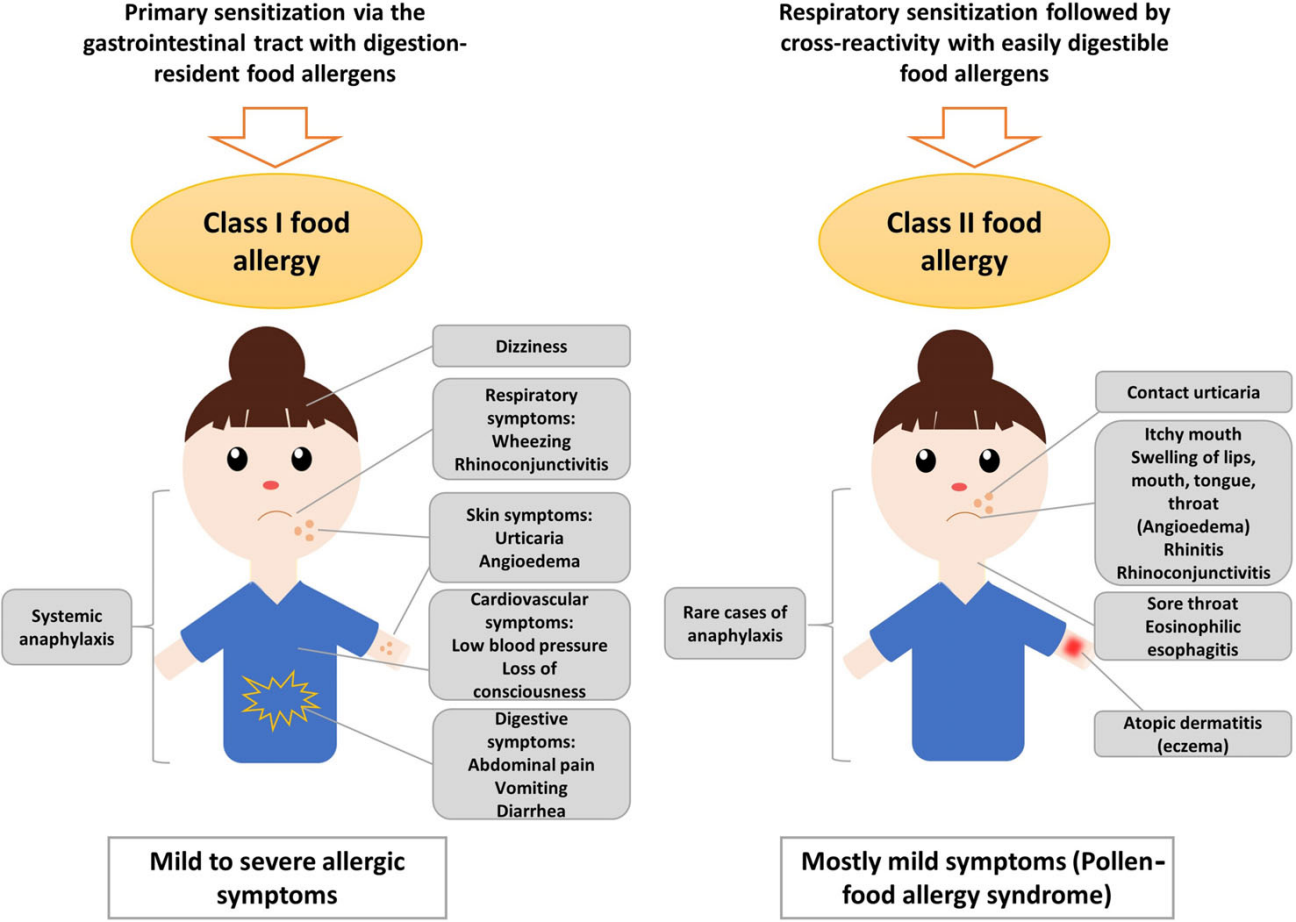
Sensitization to class I and class II nut allergens is associated with different clinical symptoms. Sensitization to class II nut allergens usually occurs by respiratory sensitization to cross-reactive respiratory allergens (e.g., pollen allergens) and is associated with mild symptoms such as oral allergy syndrome, local reactions in the oropharynx, esophagus and may trigger atopic dermatitis and/or urticaria. Sensitization to class I digestion-resistant nut allergens usually occurs *via* the gastrointestinal tract and eventually *via* the skin and is associated with systemic and severe manifestations such as anaphylaxis but also mild symptoms are possible.

True food allergy (class I) is characterized by the primary sensitization to the allergy-causing food *via* the gastrointestinal tract (12, 13) (**Figure 1**). Therefore, class I food allergens have usually higher stability to gastric digestion than other allergens (14). Immediate allergic reactions to nuts in sensitized patients occur within minutes after nut ingestion. It has been also speculated that IgE sensitization to class I food allergens may occur by epicutaneous sensitization (15) but on the other hand it was found that epicutaneous allergen application does not induce or boost allergen-specific IgE responses (16).

The severity of the allergic reaction depends on the amount of allergen to which the patient is exposed and on other factors such as barrier function and allergen-specific sensitivity which often is associated with specific IgE levels. Class I food allergens often contain sequential IgE epitopes in addition to conformational IgE epitopes which indicates that sensitization occurs also to allergen fragments emerging through digestion in the gastrointestinal tract (17–19). Allergic reactions to nuts are typically IgE-mediated (type I reactions) and might cause symptoms affecting the gastrointestinal tract (abdominal pain, vomiting), the skin (urticaria, angioedema), the respiratory tract (rhino-conjunctivitis, wheezing) and, in severe cases, the cardiovascular system (loss of consciousness, low blood pressure) (**Figure 1**). Anaphylactic shock characterized by drop in blood pressure and cardiovascular failure involves several organ systems and requires immediate treatment with epinephrine (20). Several factors such as mast cell activation and/or load, existing co-allergies or asthma might enhance the risk of anaphylactic reactions to tree nuts (21).

Class II food allergy is associated with sensitization to pollen allergens. Patients are usually first sensitized to a pollen allergen and produce IgE antibodies which cross-react with allergens present in food. Examples include the major birch pollen allergen, Bet v 1 and the panallergen, profilin which were discovered first in birch pollen (22–26). IgE sensitization to class II food allergens is usually associated with mild allergic reactions known as pollen-food allergy syndrome (PFAS) or oral allergy syndrome (OAS) (20, 27). Clinical characteristics of PFAS include mainly oropharyngeal symptoms (27). Interestingly, it has been indicated that ingestion of birch pollen-related allergens from food sources such as Cor a 1 from hazelnut, could activate allergen-specific T cells independent of IgE, leading to late-phase and chronic allergic inflammation and this might further cause disorders such as atopic dermatitis in sensitized patients (28, 29). Moreover, pollen-related nut allergens causing PFAS might be associated with eosinophilic esophagitis, although they seem to be of less relevance than homologues from fruits and vegetables (30). However, eosinophilic esophagitis can be caused also by class I food allergens from milk, egg and wheat, while peanut and tree nuts seem to be of minor relevance (31). Major features of class II food allergens are that they contain mainly conformational but not sequential IgE epitopes which are sensitive to digestion and heating (32–34). The sensitization to class II food allergens is initially caused by pollen allergens and results in IgE and T cell cross-reactivity with the related food allergens (35, 36). IgE sensitization to class II food allergens is highly prevalent in countries with high exposure to the cross-reactive pollen allergens (37, 38). Accordingly, diagnostics including the measurement of IgE against the originally sensitizing pollen allergens (39) and allergen-specific immunotherapy to the cross-reactive pollen allergens can improve not only pollen allergy but also the associated food allergy to some extent (40, 41).

Diagnosis of nut allergy usually starts with a thorough evaluation of the patient’s history. Allergic sensitization can be detected by skin prick tests (SPT) and *in vitro* diagnostics with allergen extracts. However, sensitization determined by measurement of specific IgE antibodies and SPT does not always indicate clinical food allergy, which can only be confirmed by the occurrence of specific allergic symptoms after food exposure. Double-blind placebo-controlled food challenges (DBPCFC) are still considered the “gold standard” of food allergy testing, although patients are at risk of anaphylaxis during the procedure (42, 43). Lip dose challenge (LDC) is another possibility of testing which has a good predictive value for nut allergy (44). However, in recent years, molecular diagnosis with defined and mainly recombinant allergens by IgE serology has turned out to be very helpful in diagnosing nut allergy, in particular when it is combined with a thorough medical history (45). Another key problem in therapy of nut allergy is the lack of modern and effective allergen-specific treatment options. At present, avoidance of the disease-causing allergens is a possible option but there is also evidence that early introduction of for example of peanuts in the diet of sensitized but not yet symptomatic children may have beneficial effects (46). Accordingly, there are different opinions whether avoidance or rather intake should be recommended for sensitized children. Another major problem is that there is currently little progress regarding the development of modern molecular immunotherapy forms for nut allergy. Hypoallergenic allergen derivatives have been described (47) but no clinical studies have been performed so far. Sensitization to different nut allergens varies in different parts of the world due to dietary habits in diverse populations and varying allergen exposure in different areas but this is undergoing changes due to globalization and migration.

## 2 Importance of Various Nuts as Allergen Sources in Different Parts of the World

The prevalence of nut allergies among children and adults has been investigated in several studies (11, 48–51). However, there are large variations regarding methodology and study design which make it difficult to compare the studies and to understand the true nut allergy rates. It seems that reports on nut allergy prevalence do not provide accurate information regarding actual prevalences in the different populations due to several reasons. First of all, most studies that include a representative study population are limited to self-reports and do not contain a detailed clinical evaluation of patients. Moreover, several studies do not distinguish between sensitization to class I and class II food allergens. In this context, it must be considered that allergic reactions to nuts might be due to cross-reactivity with pollen allergens and are not caused by primary nut sensitization (11). Especially in studies from Europe, hazelnut allergy prevalence might thus be overestimated and sensitization should therefore be evaluated by molecular diagnosis to clearly distinguish between birch pollen allergic patients and those with true hazelnut allergy. This applies also to several other nuts that contain cross-reactive panallergens and cross-reactive carbohydrate determinants (CCDs). For example, many subjects who were tested positive by IgE serology using peanut allergen extracts in Zimbabwe were found to be sensitized to CCDs which usually do not cause allergic reactions (52).

**Table 1** provides an overview of nut allergy prevalence studies, in particular from Europe, Northern America, Asia, Australia and Africa (38, 48–50, 52–84).

**Table 1:** Importance of peanut and different tree nuts as allergen sources in different parts of the world.

Region	Test methodology	Subjects tested	Allergens tested	Results	References
**Europe (11 countries), USA, Australia**	Sera were screened for specific IgE to food allergen mixes and individual foods using ImmunoCAP. Test was considered positive if sIgE ≥ 0.35 kU_A_/L.	4522 young adults (aged 20–44) were tested for at least one allergen mix. 4220 were tested for all five food allergen mixes. Participants had been previously included in the “random sample” group during the second phase of the European Community Respiratory Health Survey.	Walnut Peanut Hazelnut (no information on individual allergen molecules)	Sensitization by country (%): **Walnut:** Germany (3.3), Italy (3.1), France (3.7), Belgium (2.5), USA (2.1), Australia (2.1), Spain (3.1), Norway (0.6), Sweden (1.1), UK (0.8), Iceland (0.0), **Overall (2.2, excluding birch positive 1.8)** **Peanut:** Germany (4.2), Italy (3.6), France (3.0), Belgium (2.0), USA (9.3), Australia (3.0), Spain (1.9), Norway (0.8), Sweden (1.0), UK (1.5), Iceland (1.2), **Overall (2.6, excluding birch positive 1.8)** **Hazelnut:** Germany (14.7), Italy (7.7), France (5.0), Belgium (6.0), USA (14.9), Australia (4.1), Spain (2.6), Norway (12.8), Sweden (11.8), UK (4.9), Iceland (0.4), **Overall (7.2, excluding birch positive 3.1)**	(48)
**Europe (8 centers: Zurich, Madrid, Utrecht, Lodz, Sophia, Athens, Reykjavik and Vilnius)**	Questionnaire followed by serum analysis. Detection of IgE sensitization to groups of food allergens and individual foods using ImmunoCAP, which was considered positive if sIgE ≥ 0.35 kU_A_/L. Sera of subjects were tested for IgE reactivity to specific food allergens using an allergen microarray assay.	Serum samples taken from in total 719 potentially-food allergic adults (aged 20-54) and 1642 controls. Up to 240 potentially food-allergic subjects per center and 240 controls, oversampling of centers with less than 240 cases (applied for all centers).	Hazelnut Walnut Peanut (individual allergen molecules tested)	IgE sensitization to food allergy by center (first number: percentage of weighted IgE-sensitization prevalence; second number: percentage of weighted IgE-sensitization prevalence to “true” food allergens not associated with cross-reactivity to plant pollen allergens): **Hazelnut**: Zurich (17.8, 1.1), Madrid (6.0, 2.4), Utrecht (12.0, 0.0), Lodz (6.5, 0.3), Sofia (6.3, 3.0), Reykjavik (1.3, 0.7), **Overall (9.3, 0.9)** **Walnut:** Zurich (5.6, 0.1), Madrid (7.7, 0.4), Utrecht (1.9, 0.1), Lodz (3.6, 0.3), Sofia (2.7, 0.0), Reykjavik (0.1, 0.0), **Overall (3.0, 0.1)** **Peanut:** Zurich (5.0, 0.4), Madrid (7.2, 0.5), Utrecht (1.6, 0.1), Lodz (3.1, 0.0), Sofia (1.8, 0.0), Reykjavik (0.5, 0.1), **Overall (2.7, 0.14)**	(50)
**UK (Isle of Wight)**	Clinical peanut allergy and/or IgE sensitization of participants was determined. Sensitization to peanuts determined by a wheal size ≥ 3 mm in presence of negative control during SPT. Clinical allergy was confirmed by positive SPT and convincing history or positive OFC (only Cohorts B and C).	Peanut allergy prevalence was assessed in three cohorts of children born on the Isle of Wight. Cohort A: 2181 children (aged 4) born in 1989 Cohort B: 1273 children (aged 3-4) born between 1994-1996 Cohort C: 891 children (aged 3) born between 2001-2002 Number of patients included for evaluating clinical allergy: Cohort A: 1218 Cohort B: 1273 Cohort C: 891 Number of patients tested by SPT: Cohort A: 981 Cohort B: 1246 Cohort C: 642	Peanut (commercial extracts)	Percentage of sensitization: Cohort A: 1.3 Cohort B: 3.3 Cohort C: 2.0 Percentage of clinical peanut allergy diagnosis based on positive SPT and clinical history or positive OFC: Cohort A: 0.5 Cohort B: 1.4 Cohort C: 1.2	(53)
**UK**	Families were chosen by primary questionnaire. Mothers were asked about their peanut allergy status and dietary changes regarding peanut consumption during pregnancy. Selected children underwent SPT. SPT was considered positive if wheal size ≥ 3 mm in presence of negative control and wheal size of at least 3 mm to histamine (1:10w/v). Sera from children with positive SPT were tested for peanut-specific IgE using ImmunoCAP. Those showing IgE sensitization underwent OFC.	1072 mother-child pairs (children aged 3-6 years) were chosen for allergy testing based on valid questionnaire.	Peanut (allergen extracts for SPT, peanut flour-based biscuits for OFC)	30 of 1072 children (2.8%) showed IgE sensitization to peanut, confirmed by positive SPT or high peanut-specific IgE levels (> 100 kU_A_/L). 21 children underwent DBPCFC of which 15 had positive results. 5 children had convincing medical history and supportive blood and skin test results. 20 of 1072 children (1.8%) were considered to have clinical peanut allergy.	(54)
**UK**	Participants were recruited prenatally. At the age of 8 years information on exposure and reactivity to peanut was collected by a questionnaire. Peanut sensitization was confirmed by SPT with wheal size of at least 3 mm greater than the negative control and/measurement of IgE (≥ 0.2 kU_A_/l) using ImmunoCAP. Sensitized patients underwent OFC. Three children underwent open OFC with roasted peanuts. Peanut-consuming children underwent open OFC with peanut protein in brownies. All others underwent DBPCFCs with peanut protein-containing brownies. The sensitization profile of peanut-allergic children was compared to the profile of those who were considered tolerant using microarray assays.	933 children at age 8 years (unselected population-based birth cohort)	Peanut (tested for individual allergen molecules; peanut protein in brownies for OFC and DBPCFC; three children with milk/egg allergy underwent open OFC with roasted peanuts)	110 of 933 children (11.8%) were considered sensitized to peanut. 19 were not further challenged. 12 children were considered peanut-allergic due to reports of allergic reactions together with sIgE ≥ 15 kU_A_/L and/or SPT ≥ 8 mm without further challenge. Of the remaining 79 subjects that underwent OFC, 7 were considered allergic due to showing objective symptoms. Ara h 2 was the most relevant predictor of clinical peanut allergy.	(55)
**UK**	Evaluation of ethnic differences in nut sensitization profiles. Sensitization was assessed by SPT. Wheal size ≥ 3 mm was considered sensitization and ≥ 8 mm was defined as allergy.	Data from 2638 patients was collected (new referrals at the children’s allergy clinic in Leicester).	Almond Hazelnut Peanut Pecan Brazil nut Cashew Pistachio Walnut (allergen solutions and whole nuts for SPT)	Nut sensitization (SPT wheal ≥ 3 mm) and allergy (SPT wheal ≥ 8 mm) in south Asian children (percentage of all tested): **Almond**: 61.9, 7.4 **Brazil nut:** 17.2, 1.5 **Cashew:** 69.1, 27.4 **Hazelnut:** 30.5, 4.2 **Peanut:** 63.2, 30.3 **Pistachio:** 64.3, 25.7 **Pecan:** 26.9, 8.3 **Walnut:** 30.1, 8.1 Nut sensitization (SPT wheal ≥ 3 mm) and allergy (SPT wheal ≥ 8 mm) in White children (percentage of all tested): **Almond:** 31.1, 1.8 **Brazil nut:** 20.1, 5.4 **Cashew:** 35.6, 10.6 **Hazelnut:** 25.8, 2.4 **Peanut:** 64.7, 36.1 **Pistachio:** 31.2, 6.9 **Pecan:** 24.7, 7.1 **Walnut:** 20.8, 5.4	(56)
**UK** **Israel**	Participants completed validated questionnaires about food allergy (schoolchildren) or food consumption (infants) (period 2004-2005). Food frequency questionnaire was completed by the mothers of the infants. Food allergy questionnaires in primary schools were completed by the children’s parents. Children with questionnaire-based peanut allergy were invited for allergy testing (SPT, sIgE or both) which was considered positive if results were > 95% positive predictive values or in case of positive OFC.	The food allergy questionnaire was returned by 4148 Jewish schoolchildren (aged 4-19 years) in the UK and 4672 in Israel. Food frequency questionnaire included 77 Jewish infants (aged 4-24 months) in the UK and 99 in Israel.	Peanut Tree nuts (questionnaire based; products for allergy testing not further specified)	Questionnaire-based peanut allergy prevalence was 1.85% in the UK and 0.17% in Israel. Of peanut-allergic children, 58.9% (43 of 73) in the UK and 50% (4 of 8) in Israel had tree nut allergy. Dietary introduction of peanut occurred earlier in Israel than the UK. At age 9 months, 69% of infants in Israel and 10% in the UK were eating peanuts.	(57)
**Denmark**	Questionnaire followed by SPT, histamine release (HR) assay and OFC. Positive SPT was defined as wheal size ≥ 3 mm. Histamine release of ≥ 10 ng/ml was considered as positive. DBPCFC was performed for peanut using peanut-containing chocolate bars. Distinction made between primary food hypersensitivity (FHS) (independent of pollen) and secondary FHS (pollen allergic patients).	Total study population were 1272 unselected young adults (age 22 years), of which 843 responded to questionnaire and were included in the analysis.	Peanut Almond Hazelnut Brazil nut Walnut (only peanut was used for SPT (fresh peanut) and OFC (peanut in chocolate bars))	223 of 843 subjects that returned the questionnaire suspected FHS. Of those, 165 self-reported primary FHS (independent of pollen) and 141 secondary FHS (pollen-associated). **Prevalence of primary FHS:** **Peanut:** Self-reported n (%): 45 (5.3) Challenged n: 12 Confirmed by OFC n (%): 5 (0.6) **Prevalence of secondary FHS (only self-reported):** **Almond** n (%): 2 (0.2) **Brazil nut** n (%): 23 (2.7) **Hazelnut** n (%): 56 (6.6) **Walnut** n (%): 4 (0.5)	(58)
**France**	Clinical symptoms of asthma, allergic rhinitis and food allergy assessed using a questionnaire that was completed by the parents. Evaluation of food allergy prevalence and its association with respiratory manifestations of allergy by SPT to food and aeroallergens. For positive SPT wheal size had to be ≥ 3 mm and greater than the negative control.	In total, 6672 schoolchildren (aged 9-11 years) from 108 randomly chosen schools were recruited for clinical examination and completed the questionnaire.	Peanut Tree nuts, only listed as “nuts” and not further defined (only peanut was tested by SPT)	**Reported symptoms of FA, n (%):** **Peanut:** 21 (0.3) **Nuts:** 10 (0.2) **Food sensitization n (%):** **Peanuts:** 70 (1.1) Of the children sensitized to at least one food allergen (n = 119), 58.8% were sensitized to peanut. About 26.7% were sensitized to at least one aeroallergen. Of the 10 children that reported symptoms to nuts, 22.2% were sensitized to birch pollen.	(59)
**Finland**	Investigation of nut sensitization and cross- and co-sensitization to other nuts and birch pollen by using available SPT data. SPT was considered positive if wheal size was at least 3 mm.	50604 patients (children and adults) at the Helsinki Allergy Hospital (1997–2013), that underwent SPT to at least one nut (18603 birch-positive, 32001 birch-negative).	Peanut Hazelnut Almond Pistachio Macadamia Walnut Cashew Pecan Brazil nut (for SPT raw nuts were used (prick-to-prick method))	Of 50604 patients that were tested for nuts, 36.8% were birch positive and 63.2% were birch-negative. **Nut sensitization in birch-positive patients (%):** Hazelnut: 84 Almond: 71 Peanut: 60 Pistachio: 55 Macadamia: 45 Walnut: 41 Cashew: 28 Pecan: 21 Brazil nut: 18 **Nut sensitization in birch-negative patients (%):** Pistachio: 14 Cashew: 12 Walnut: 11 Macadamia: 10 Brazil nut: 8 Pecan: 8 Peanut: 7 Almond: 6 Hazelnut: 5 In a subgroup of patients without birch sensitization, children <5 years were most commonly nut‐sensitized (8–40%), with decreasing prevalence with age. Cross-reactivity was strongest between cashew and pistachio and pecan and walnut.	(60)
**Turkey**	Initial selection based on 6963 available questionnaires of subjects with suspected food allergy, followed by a telephone interview. Clinical diagnosis of consented patients by SPT, physical examination, sIgE and OFC. SPT was considered positive if wheal size was at least 3 mm in comparison to the negative control. SIgE was measured by ImmunoCAP.	Study included 6963 schoolchildren (aged 10-11 years) from the multicenter ISAAC Phase II study (2005-2006). 1162 children, including 909 symptom-positive, 301 SPT-positive and 48 for which applied both were selected and 813 participated in a telephone interview. Of 152 adolescents with current complaints, 87 agreed to clinical investigation.	Peanut Hazelnut Walnut Pistachio (commercial extracts or prick-to-prick testing)	Percentage of parental-reported food allergy prevalence in the ISAAC Phase II study population (n = 6963): **Pistachio:** 0.8 **Walnut:** 1.2 **Peanut:** 1.4 **Hazelnut:** 1.5 Percentage of SPT-confirmed prevalence in the ISAAC Phase II study population: **Hazelnut:** 0.4 **Peanut:** 0.7 **Walnut:** 4.5 In total, 12 food allergies were diagnosed in 9 adolescents including allergy to peanut (n = 1), hazelnut (n = 1) and walnut (n = 3).	(61)
**Turkey**	Pre-selection by questionnaire, clinical evaluation by SPT and DBPCFC. SPT was considered positive if wheal size diameter was at least 3 mm in presence of a negative control and a positive histamine reaction after 15 minutes. DBPCFC was preceded by a 7-day elimination diet.	2739 of 3500 randomly selected schoolchildren (aged 6-9 years) from the eastern Black sea region of Turkey returned questionnaire. SPT was performed in 145 children and DBPCFC was performed in 44 children.	Hazelnut Walnut Peanut (commercially available extracts for SPT; DBPCFC performed with all three nuts, masked in chocolate pudding)	Of the 2739 subjects that returned the questionnaire, 156 had parent-reported IgE-mediated food allergy and were further recruited for a second-phase study. Of these 156 children, 145 underwent SPT of which 48 were considered positive to at least one food. 41 children with positive SPT and 3 with negative SPT underwent DBPCFC. Nuts that were most commonly associated with allergic reactions (of total foods reported (n = 256), food positive in SPT (n = 88) and positive in OFC (n = 22)): **Hazelnut:** Reported n (%): 8 (3.1) SPT n (%): 2 (2.2) DBPCFC n (%): 0 (0) **Peanut:** Reported n (%): 3 (1.1) SPT n (%): 2 (2.2) DBPCFC n (%): 0 (0) **Walnut:** Reported n (%): 3 (1.1) SPT n (%): 2 (2.2) DBPCFC n (%): 0 (0)	(62)
**Turkey**	Pre-selection of adolescents by parental questionnaire and phone survey. Clinical evaluation in selected patients by SPT (positive if wheal diameter at least 3 mm), measurement of specific IgE by ImmunoCAP (> 0.35 kU_A_/L for positive result) and DBPCFC.	10,096 parents of schoolchildren (aged 11-15 years) responded to a questionnaire. Of those, 1139 reported food allergy of their children and were selected for phone survey. Finally, 107 adolescents were selected for clinical evaluation.	Peanut Walnut Hazelnut Almond Pistachio (commercially available extracts for SPT; hazelnut peanut and walnut were masked in chocolate pudding for DBPCFC)	Clinical evaluation (n = 107) of pre-selected patients with suspected food allergy: **Walnut (n = 14):** SPT positive: 4/14 sIgE positive: 3/14 DBPCFC: 4/5 **Hazelnut (n = 11):** SPT positive: 1/11 sIgE positive: 1/11 DBPCFC: 1/3 **Peanut (n = 9):** SPT positive: 6/9 sIgE positive: 3/9 DBPCFC: 4/6 **Almond (n =1):** SPT positive: 0/1 sIgE positive: 0/1 No DBPCFC **Pistachio (n =1):** SPT positive: 1/1 sIgE positive: 1/1 No food challenge due to history of anaphylaxis The most common foods causing allergies were peanut (0.05%) and tree nuts (0.05%).	(63)
**Russia**	Initially, parents completed the international ISAAC questionnaire. Based on the questionnaire two groups of children were formed (with and without symptoms of allergy). All children underwent SPT and sera from both groups were tested for sIgE using microarray-based allergen chip (MeDALL allergen chip). Allergen-specific IgE level of = or > 0.3 ISU was considered positive.	In total, 200 children that attended the National Research Center—Institute of Immunology Federal Medical‐Biological Agency of Russia with their parents were included in this study. Group 1: Children with allergic symptoms (n = 103; 12.24 ± 2.23 years) Group 2: Children without allergic symptoms (Group 2: n = 97; 12.78 ± 2.23 years)	Hazelnut Peanut Walnut Cashew Pistachio Brazil nut (tested for individual allergen molecules)	Food allergen-specific IgE sensitization was dominated by cross-reactive allergens (PR10 proteins) such as rAra h 8 (peanut) rCor a 1 (hazelnut), with the latter being among the most frequently recognized allergens (52.4%) in symptomatic children. Within the group of symptomatic children (n = 103) the following nut allergens were recognized, n (%): rCor a 1 (hazelnut): 54 (52.4) rAra h 8 (peanut): 47 (45.6) nJug r 2 (walnut): 15 (14.5) rJug r 3 (walnut): 8 (7.8) rAra h 9 (peanut): 6 (5.8) rCor a 8 (hazelnut): 5 (4.8) nAna o 2 (cashew): 2 (1.9) nCor a 9 (hazelnut): 2 (1.9) rJug r 1 (walnut): 2 (1.9) rAna o 3 (cashew): 1 (0.9) rAna o 1 (cashew): 1 (0.9) nAra h 1 (peanut): 1 (0.9) rPis v 3 (pistachio): 1 (0.9) rCor a 14 (hazelnut): 0 rBer e 1 (Brazil nut): 0 rPru du 3 (Almond): 0 rPru du 4 (Almond): 0 rPru du 6 (Almond): 0 rPru du 6.01 (Almond): 0 rPru du 6.02 (Almond): 0 nAra h 3 (Peanut): 0 nAra h 6 (Peanut): 0 rAra h 2 (Peanut): 0 Similarly, recognition of PR10 proteins predominated in subjects without symptoms. Of genuine nut allergens not associated with respiratory sensitization, walnut allergens were most commonly recognized in the symptomatic group. The lack of reactivity to peanut storage proteins suggests low prevalence of peanut allergy in Russia.	(38)
**Iran**	Initial questionnaire in 2 different groups: population within the Kerman Province, the largest pistachio cultivation region of the world, and a population outside this region. Adults completed the questionnaire themselves or as guardians for their children. Clinical evaluation by SPT and testing of *in vitro* cross-reactivity with other nuts by Western blot and inhibition ELISA. SPT was considered positive with a wheal diameter > 3 mm in regard to the negative control.	1724 subjects responded to the questionnaire. Within the pistachio cultivation region were 564 responses (average age 31.35 ± 13.6 years). In the population outside this region were 1160 responses received (average age 37 ± 10 years). Clinical evaluation of 21 patients. Testing of IgE-cross-reactivity in 3 pistachio-allergic patients.	Pistachio (protein extracts used for SPT) Cashew Almond Peanut (protein extracts used for cross-reactivity study)	Questionnaires revealed a pistachio allergy prevalence of 0.65% within the pistachio cultivation site and a prevalence of 0.3% for outside this region based on reports of allergic reactions to pistachios. Cross-reactivity between pistachio and cashew was shown, followed by partial cross-reactivity between pistachio and almond (determined by inhibition ELISA).	(64)
**Iran**	Medical record review of patients referred to the Immunology and Allergy Medical Center of Khatam Hospital during a 7-year period (1996-2003). Patients underwent SPT and responded to a questionnaire. SPT with wheal diameter > 3 mm in regard to the negative control and flare diameter of > 10 mm were considered positive.	1286 allergic patients (aged 2-79 years) were included.	Walnut Hazelnut (no information on individual allergen molecules)	29.16% of patients were sensitized to walnut and 15.32% were sensitized to hazelnut, determined by positive SPT.	(65)
**South Korea**	Retrospective medical record review performed in 14 university hospitals in South Korea (2009–2013) in order to collect cases of anaphylaxis that were caused by peanut, tree nuts or seeds. Measurement of sIgE levels using ImmunoCAP and SPT. SPT was considered positive if wheal diameter > 3 mm or ≥ the histamine control.	Pediatricians identified 991 cases of anaphylaxis in patients (< 19 years) based on retrospective medial record review. IgE data of 104 patients available, 11 patients underwent SPT.	Peanut Walnut Almond Hazelnut Cashew Pistachio Pecan Macadamia (products used for testing not specified)	In total, 126 of 991 cases of anaphylaxis were caused by peanut, tree nuts or seeds. Affected patients were between 0.8 and 18.9 years old (over 80% of children < 7 years old). Nuts that caused anaphylaxis, n (%): **Peanut:** 41 (32.5) **Walnut:** 52 (41.3) **Cashew:** 6 (4.8) **Almond:** 3 (2.4) **Hazelnut:** 3 (2.4) **Pecan:** 3 (2.4) **Pistachio:** 1 (0.8) **Macadamia:** 1 (0.8) In 104 cases, sIgE levels were measured. Median sIgE levels to peanut and walnut were 10.50 and 8.74 kU_A_/L.	(66)
**China**	Medical records of patients at the First Affiliated Hospital of Zhengzhou University, Henan Province, China (2012-2016) were retrospectively analyzed. SIgE of Patients was measured by AllergyScreen test with sIgE ≥ 0.35 IU/mL being considered positive.	Medical records of 15534 patients with suspected allergy were included. The study population included 7388 males and 8146 females (5257 children and 10277 adults). The average age was 30.56 ± 20.98 years.	Cashew (no information on individual allergen molecules)	Cashew nut was one of the most frequent tested food allergens (n = 1320, 8.5%).	(67)
**China**	Parents that attended routine baby health checks with their children at the Department of Primary Child Care, Children’s Hospital of Chongqing Medical University were asked to complete a questionnaire. Children underwent SPT. Wheal size of ≥ 3 mm greater than the negative control was considered positive. Children with positive SPT or positive medical history were asked to undergo OFC (not for peanut).	497 infants and young children (aged 0-12 months) were included in the study, of which 477 fully participated.	Peanut (product used not further specified)	In 46 of 497 cases parents reported allergic reactions of their children to food. 2 subjects had positive SPT to peanut.	(68)
**China**	Two cross‐sectional studies were performed, the first in 1999 and the second in 2009. Children that attended the division of Primary Child Care, Children’s Hospital, Chongqing Medical University for well-baby checking were randomly enrolled. Parents completed an initial questionnaire. Subsequently, all subjects underwent SPT. SPT was considered positive if wheal diameter was at least 3 mm larger than the negative control. Elimination diet was followed by OFC if positive effect of food elimination was observed.	In total, 401 infants were randomly selected (0-24 months), and 382 were included in the final analysis (in study from 2009). Results were compared with study from 1999. In 1999, 314 questionnaires were returned and infants were skin prick tested. 10 infants dropped out during food elimination, thus, 304 were included in the final analysis.	Peanut (Extracts or prick-to-prick technique used for SPT; peanut butter used for oral provocation)	Of 32 infants with positive SPT in 1999, 1 showed reactivity to peanut. In 2009, 72 infants had positive SPT, including 6 that reacted to peanut. In 1999, peanut was among the offending foods causing food allergy in infants (observed in 1 of 11 children with challenge-confirmed food allergy). In 2009, confirmed food allergy only included egg and cow´s milk.	(69)
**Singapore**	Retrospective study to evaluate clinical features of peanut allergy in children in the largest pediatric hospital in Singapore. Peanut allergy was diagnosed based on medical history, together with SPT (positive of wheal diameter of ≥ 3 mm in comparison to the negative control), sIgE (positive for sIgE ≥ 0.35 kU_A_/L) and OFC.	269 children (≤ 16 years old) with clinical diagnosis of peanut allergy were included.	Peanut Cashew Almond Hazelnut Walnut (SPT with commercial extracts; OFC using peanut butter or roasted peanuts)	269 patients that were diagnosed with peanut allergy were identified, together with 59 patients that were considered peanut tolerant (positive SPT, but tolerant to peanut ingestion). In the peanut allergy group, the median age of first allergic presentation was at 24 months. The rate of anaphylactic reactions in the study population was 7.1%. In the peanut allergy group, 32.3% were also sensitized to the following tree nuts: cashew nut (17.1%), almond (15.6%), hazelnut (15.6%), walnut (14.1%).	(70)
**Singapore** **Philippines**	Administration of a questionnaire to assess prevalence of peanut and tree nut allergy in Singapore (local and expatriate) and Philippine schoolchildren of different age groups. Allergy diagnosis was based on convincing history which was defined by reports on the appearance of specific allergic symptoms within two hours after food ingestion.	In total, 25,692 schoolchildren responded to the survey. Of these, 23,425 children (4-6 and 14-16 years) were included in the final analysis. The analysis included 4515 local Singapore children (4-6 years old), 6498 local Singapore children (14-16 years old), 978 Singapore expatriates (4-6 and 14-16 years old) and 11434 Philippine children (14-16 years old).	Peanut “Tree nuts” including the following: Almond Brazil nut Cashew Hazelnut Macadamia Pecan Walnut (only questionnaire based)	**Peanut allergy prevalence based on convincing history:** Singapore (4-6 years: 0.64% Singapore (14-16 years): 0.47% Philippines (14-16 years): 0.43% **Tree nut allergy prevalence based on convincing history:** Singapore (4-6 years): 0.28% Singapore (14-16 years): 0.30% Philippines (14-16 years): 0.33% Higher rates of peanut and tree nut allergy were reported in Singapore expatriates: Peanut (4-6 years): 1.29% Peanut (14-16 years): 1.21% Tree nuts (4-6 years): 1.12% Tree nuts (14-16 years): 1.21 Most common reported tree nuts (decreasing order of frequency) were cashew, hazelnut, almond, walnut, macadamia, pistachio, pecan and Brazil nut.	(71)
**Singapore**	Patients from the allergy database at Kandang Kerbau Children’s Hospital (KKH), Singapore, with positive SPT or peanut-specific ImmunoCAP FEIA < 0.35 kU_A_/L were selected (2003-2006). Eligible patients completed a questionnaire. Specific serum IgE to Ara h 1, Ara h 2 and Ara h 3 was detected by ELISA. Peanut-specific IgE was detected using CAP-FEIA.	31 patients (aged 0.7-13.2 years) consented to the study (of 62 eligible patients).	Peanut (specific IgE to Ara h 1, 2, 3 was measured; commercial extracts used for SPT)	SPT wheal size of the 31 tested patients ranged from 3-17 mm. 28 patients had positive peanut-specific IgE. 87.1% had IgE specific to Ara h 1, 87.1% to Ara h 2 and 54.8% to Ara h 3.	(72)
**Singapore**	Retrospective study of Singaporean children that experienced anaphylaxis and visited a tertiary pediatric hospital between 2005-2009. Patients with history of anaphylaxis underwent SPT. SPT was considered positive if wheal size was ≥ 3 mm compared to the negative control.	98 children (aged 3.6-10.8 years) included in study (108 cases of anaphylaxis).	Peanut Tree nuts (commercial extracts used for SPT)	Peanut was the most common food trigger of anaphylaxis (19%). Tree nuts accounted for 4% of anaphylaxis.	(73)
**Taiwan**	Serum was collected and sIgE to individual nuts was measured (positive if sIgE ≥ 0.35 kU_A_/L) using ImmunoCAP.	333 patients (aged 2-93 years) from the outpatient department of Kaohsiung Veterans General Hospital, Taiwan that showed symptoms of asthma, atopic dermatitis and allergic rhinitis were included in the study (from 2014-2017).	Peanut Cashew Brazil nut Almond (no information on individual allergen molecules)	In total, 555 sIgE data were available, of which 339 were considered as food sensitization (≥ 0.35 kU_A_/L), including peanut (n = 124, 36.6%), cashew nut (n = 64, 18.9%), Brazil nut (n = 28, 8.3%) and almond (n = 73, 21.5%).	(74)
**Japan**	A questionnaire was provided to the participants in order to collect data on anaphylaxis-causing foods.	1383 individuals from 878 families (including 319 patients with history of anaphylaxis) provided a valid questionnaire. Average age was 11.25 years (range, 0–93 years). The most frequently recorded age was 5 years.	Peanut (only questionnaire based)	27 of 319 patients (8.5%) reported peanut-related anaphylaxis. In comparison, anaphylaxis to milk, eggs and wheat was reported by 221 (69.3%), 144 (45.1%) and 92 (28.8%) patients, respectively.	(75)
**USA**	Follow-up study to determine prevalence of peanut and tree nut allergy in the USA by a nationwide, cross-sectional random phone survey. Allergic reactions were considered “convincing” if specifically defined allergic symptoms were reported.	5300 households (13,534 subjects) were surveyed (children and adults from 0 to ≥65 years).	Peanut Walnut Cashew Pecan Almond Pistachio Brazil nut Macadamia (only questionnaire based)	Overall prevalence of peanut allergy (children and adults): 0.8% Overall prevalence of tree nut allergy (children and adults): 0.6% For children < 18 years the prevalence of peanut or tree nut allergy was 2.1%, compared with 1.2% in 2002 and 0.6% in 1997. Number of participants reporting tree nut allergy: **Walnut:** 41 **Cashew:** 29 **Pecan:** 26 **Almond:** 25 **Pistachio:** 19 **Brazil nut:** 19 **Hazelnut:** 17 **Macadamia:** 17	(76) Previous studies: (77, 78)
**Mexico**	Cross-sectional, observational, retrospective trial. Data registries (2016-2018) from an allergy laboratory in Mexico City that included patients with suspected food allergy of all ages were analyzed. Data included results of sIgE measurements using ImmunoCAP (sIgE ≥ 0.35 kU_A_/L for positive result).	In total, 2633 patients (of all ages and gender) were included in the serological testing. In the final analysis, 1795 patients with suspected clinical allergy were included.	Hazelnut Peanut Almond Cashew Pecan (no information on individual allergen molecules)	Hazelnut, peanut and almond were among the 15 most frequent foods with positive sIgE (≥ 0.35 kU_A_/L) results (number of tested patients and % of positive results of all patients tested for this food): Hazelnut: 63, 49% Peanut: 219, 25% Almond: 65, 18% Sensitization to peanut and tree nuts was more frequent in older children (aged 6-17 years). In the group of foods with low sample size (< 50) cashew showed high positivity: of 22 patients tested, 27.3% had sIgE levels of ≥ 0.35 kU_A_/L and 13.6% had sIgE levels of ≥ 0.71 kU_A_/L. Of 34 patients that were tested to pecan, 14.7% had both sIgE levels of ≥ 0.35 kU_A_/L and ≥ 0.71 kU_A_/L.	(79)
**Mexico**	Prevalence of peanut and tree nut allergy in Mexican adults assessed based on a survey. Probably allergy was defined by reports of specific allergic symptoms appearing within two hours after food ingestion.	1126 participants (50.1% young adults aged 18-24 years and 49.9% adults aged 25-50 years) were included in the study.	Peanut Pecan Hazelnut Pistachio Almond (only questionnaire based)	Due to lack of documented adverse reactions to hazelnuts, pistachios, and almonds in the tree nut category perceived and probable allergy applied only for pecan and was 0.4% and 0.3%, respectively. Perceived and probably peanut allergy was both 0.6%.	(80)
**Canada**	Food allergy prevalence was assessed by a random telephone survey. Food allergy was either defined as perceived (self-report), probable (convincing history or reported confirmation by a physician) or confirmed (convincing medical history and confirmatory test results). Confirmatory test results included positive SPT (wheal size at least 3 mm greater than the negative control), food specific serum IgE levels of IgE ≥ 0.35 kU_A_/L or positive OFC. Additionally, patients that had uncertain clinical history were considered having confirmed allergy if they had positive SPT together with sIgE of ≥ 15 kU_A_/L for peanut and tree nut or positive SPT together with positive OFC or OFC alone.	Of 10596 households, 3613 (9667 individuals) completed interview and were included in the analysis. Participation was eligible if respondents were 18 years or older. However, respondents also provided information on any additional allergic household member.	Peanut Tree nut (not distinguished between individual tree nuts) (only questionnaire based)	**Peanut allergy prevalence (%):** **Children:** Perceived: 1.77 Probable:1.68 Confirmed: 1.03 **Adults:** Perceived: 0.78 Probable: 0.71 Confirmed: 0.26 **Entire study population:** Perceived; 1.00 Probable: 0.93 Confirmed: 0.61 **Tree nut allergy prevalence (%):** **Children:** Perceived; 1.73 Probable: 1.59 Confirmed: 0.69 **Adults:** Perceived: 1.07 Probable:1.00 Confirmed: 0.35 **Entire study population:** Perceived; 1.22 Probable: 1.14 Confirmed: 0.68	(81)
**Australia**	Parents completed an initial questionnaire. Detection of IgE sensitization to foods in 1-year-old infants by SPT and those with sensitization in SPT (wheal size ≥ 1 mm compared to the negative control) underwent OFC.	2848 infants (12 months old) were included in the study. Of those, 45 did not undergo SPT because they had been already tested by their doctor.	Peanut (products used not further specified)	Prevalence of sensitization to peanut was 8.9% (wheal size ≥ 1 mm). Prevalence of clinically relevant sensitization (SPT ≥ 3 mm) to peanut was 6.4%. Peanut allergy prevalence confirmed by OFC was 3.0%.	(49)
**Australia**	**At age 1 year:** *Tree nut sensitization* was defined by SPT wheal size of at least 3 mm (compared to the negative control) to almond, cashew or hazelnut. *Tree nut tolerance* was defined by history of tolerance to food ingestion or negative SPT. *Parent reported tree nut allergy* was defined by reports of specific allergic reactions. No OFC for tree nuts was performed at age 1, but OFC performed for peanut. *Sensitized tolerance to peanut* was defined by SPT wheal size of at least 2 mm and negative OFC. *Peanut allergy* was defined by SPT wheal size of at least 2 mm and positive OFC. **At age 6 years:** *Tree nut sensitization* was defined by SPT with wheal size of at least 3 mm (compared to the negative control) to almond, Brazil nut, cashew, hazelnut, macadamia, pecan, pistachio or walnut. *Definite tree nut allergy* was defined by positive OFC and IgE sensitization or history of objective symptoms or positive OFC at age 4 years and SPT wheal size of 8 mm at age 6 years. *Probable tree nut allergy* was defined by SPT response of at least 8 mm, without reaction history or previous OFC result, SPT wheal size of 3-7 mm at age 6 years together with positive OFC at 4 years of age, history of objective symptoms or report of food avoidance due to allergy. *Tree nut tolerance* was defined by negative OFC result, SPT wheal size of 0-2 mm, SPT response of 3-7 mm and reported food ingestion, or lack of reaction since age 4 years without food avoidance.	Initially, 5276 1-year-old children were recruited. 3232 participated in the follow-up study at age 6 years and completed questionnaire and SPT assessment, while 1222 completed questionnaire only.	Cashew Almond Hazelnut Pistachio Walnut Macadamia Pecan Brazil nut (extracts for SPT)	Of the 5276 infants that participated in the study, 924 had positive SPT results to egg, sesame, peanut, shrimp or cow´s milk. The positive-tested infants further attended OFC clinic and had SPT to tree nuts. Food allergy to egg, peanut or sesame was confirmed by OFC in 530 patients. **Tree nut sensitization at age 1 year:** Of patients with challenge-confirmed food allergy, 31% were sensitized to at least 1 tree nut. Tree nut sensitization was more common in infants with both peanut and egg allergy. **Tree nut sensitization at age 6 years:** 234 children were sensitized to tree nuts at the age of 6, corresponding to 7.3% of all that underwent SPT to tree nuts (n = 3232). 154 children were considered allergic to at least one tree nut. Cashew was the most common tree nut causing allergy (2.7%), followed by hazelnut (0.9%) and almond (0.3%). Other tree nuts allergies were diagnosed in < 1.0% of the subjects (pistachio, n = 50; walnut, n = 28; macadamia, n = 12; pecan, n = 8; and Brazil nut, n = 5).	(82)
**Australia**	Preselection by questionnaires for students and parents. Clinical evaluation in eligible students by SPT and OFC in case of SPT result with wheal size of at least 3 mm. Current clinical food allergy was defined by positive OFC or convincing history including data on IgE sensitization (SPT wheal size of > 3 mm or sIgE > 0.35 kU_A_/L), or SPT with wheal size of > 8 mm.	9816 randomly selected students (aged 10-14 years) provided either a student questionnaire (history of food allergy) or a parent questionnaire (history of food allergy and additional information). 5016 students were included in the clinical evaluation.	Peanut Tree nuts: Cashew Pistachio Walnut Hazelnut Macadamia Pecan Almond Brazil nut (products used not further specified)	Clinical-defined current food allergy in the clinical group (n = 5016) had a prevalence of 4.5%. The most common foods causing allergy in the clinical group were peanut (2.7%) and tree nuts (2.3%). Among tree nuts, cashew was most prevalent (1.6%), followed by pistachio (1.0%), walnut (0.7%), hazelnut (0.7%), macadamia (0.2%), pecan (0.2%), almond (0.1%) and Brazil nut (0.1%).	(83)
**South Africa**	Evaluation of IgE sensitization to several allergen molecules by using an allergen microarray (ISAC technology-based). Values ≥ 0.1 ISU were considered positive.	166 black South African children (aged 9-38 months) from urban and rural areas with and without atopic dermatitis (AD) were included: Urban AD (n = 32) Urban non-AD (n = 40) Rural AD (n = 49) Rural non-AD (n = 45)	Peanut (tested for individual allergen molecules)	31% of urban and 41% of rural AD patients were sensitized to at least one peanut allergen. However, self-reported peanut exposure was significantly higher in urban (79%) than rural (39%) regions. In non-AD children sensitization was significantly lower. Ara h 2 (29% rural, 19% urban AD children) and Ara h 6 (25% rural, 22% urban AD children) were most commonly recognized.	(84)
**Zimbabwe** **Sweden**	Sera from peanut-sensitized and peanut-allergic patients were analyzed for IgE to Ara h 1-3, 6, 8 and 9 using an allergen microarray. IgE levels were considered low (0.35-1 ISU-E), moderate-high (> 1-15 ISU-E) or very high (> 15 ISU-E). Allergen-specific IgE to peanut extract was measured by ImmunoCAP (≥ 0.10 kU_A_/L for positive result).	54 peanut-sensitized patients from Zimbabwe (aged 0.9-59 years), 25 peanut-allergic (aged 3-15 years) and 25 peanut-sensitized, but tolerant patients (aged 3-18 years) from Sweden were included.	Peanut (tested for individual allergen molecules)	46% of African patients and all of the peanut-allergic Swedish patients had IgE to at least one highly allergenic peanut allergen (Ara h 1, 2, 3, 6 or 9). Of the African patients, 48% showed IgE toward cross-reactive carbohydrate determinants (CCDs). 60% of Swedish peanut-tolerant patients had IgE to Ara h 8.	(52)

Listed are studies investigating prevalences of allergy to different nuts as determined by different methodologies in different populations with the corresponding references.

Importantly, the worldwide prevalence of nuts causing allergy correlates strongly with the nuts that are consumed in this region. However, for improved nut allergy management it is more relevant to consider the sensitization profile of nut allergic patients on a molecular level. As an example, sensitization to allergens of the family of pathogenesis-related class 10 (PR-10) proteins is widespread in northern countries, while IgE reactivity to non-specific lipid transfer proteins (nsLTPs) is predominant in the Mediterranean region. Molecular diagnostics significantly helps to distinguish between cross-reactive allergens and those that are a true indicator of sensitization to a particular nut.

In Europe, regional as well as ethnical differences in the sensitization profile of nut allergic patients have been observed (48, 50, 56). Generally, self-reported prevalence is significantly higher than food challenge-confirmed nut allergy (58). Several studies that investigated peanut allergy prevalence in Europe revealed varying prevalence rates (53–55, 59). In Russia, peanut allergy does not seem to play a major role in food allergy (38). Peanuts and cashew nuts are among the most common elicitors of anaphylaxis (85). Co-sensitization to different nuts correlates strongest between nuts of the same botanical family such as cashew and pistachio or pecan and walnut (60).

In the US, peanut is one of the most common foods causing allergy (76–78). Among tree nuts, walnut and cashew cause most of the allergic reactions, followed by almond, pistachio, Brazil nut, hazelnut and macadamia (76). Similar results were seen in a Canadian study with peanut allergy being most prevalent, predominantly in children (81).

In Central and South America, few studies reported sensitization of allergic patients to peanut and almond, although in this region, allergy to nuts seems to be low in general (79, 80, 86, 87). In most Latin American countries, frequent foods that cause allergy include fish, seafood, milk, egg, vegetables and fruits (87, 88).

In Asia, peanut allergy prevalence seems to be low compared to US and certain western countries (76, 89–92). Cashew nut is one of the most common reported tree nuts causing allergy in Asia (67, 70, 71, 74). However, tree nut allergy prevalence varies significantly across Asia especially between East and Southeast Asia and the Middle East (62, 63, 66, 70, 74). It can be assumed that the availability of nuts in certain regions contributes to the prevalence of allergies to these nuts, as can be seen by the increased frequency of pistachio allergy in pistachio cultivation regions (64).

In Australia, peanut allergy is one of the most frequent elicitors of IgE-mediated food allergy (49, 93). Tree nut allergy in Australia is less common than peanut allergy and prevalence rates of individual tree nut allergies vary significantly between studies (82, 83, 93).

Peanut allergens are the most frequently recognized nut allergens in South Africa (84) as determined in allergic children whereas IgE recognition of peanut allergens seems to be often asymptomatic as reported for Zimbabwe (52) but data regarding the prevalence of nut allergies in Africa are rare.

**Figure 2:**
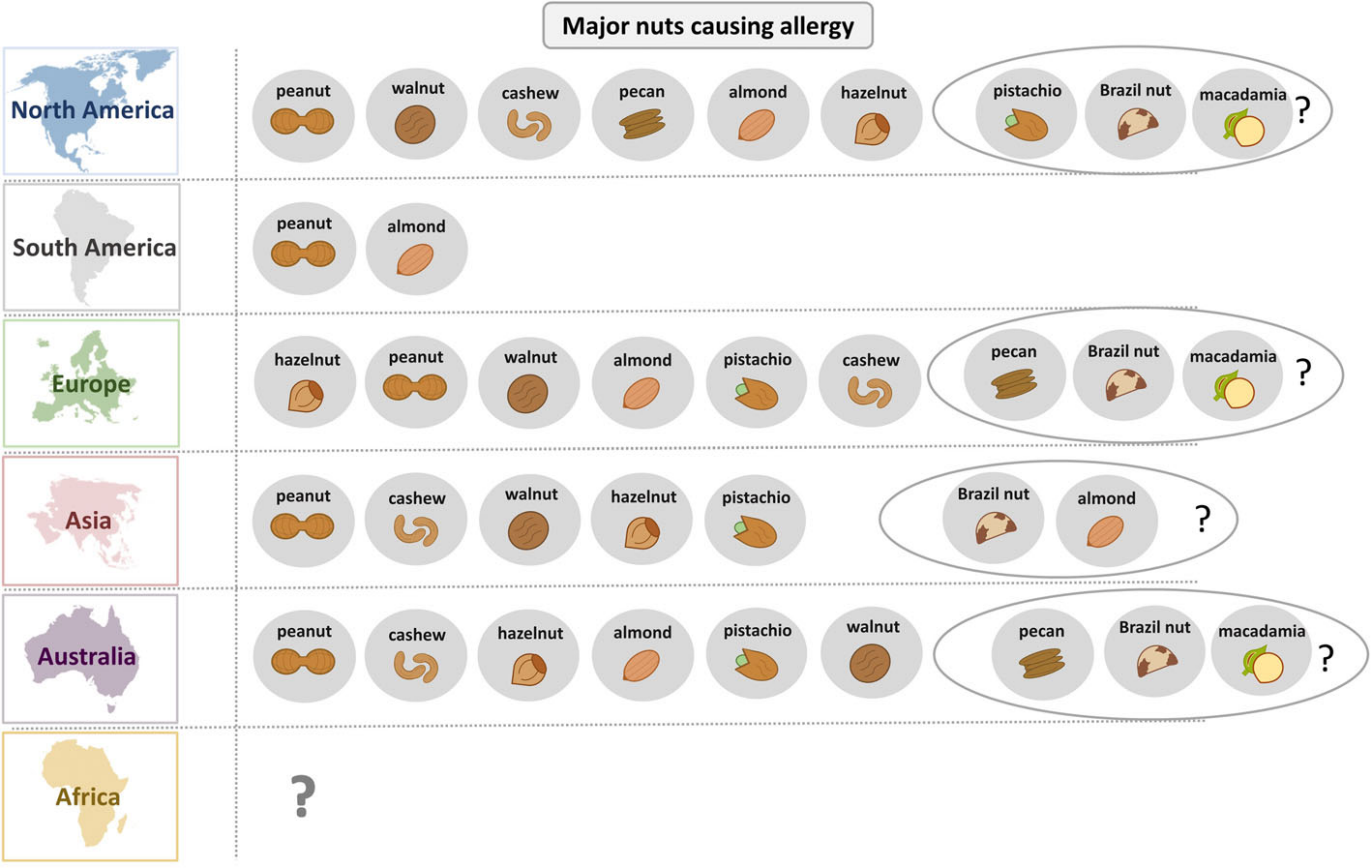
Overview of the relevance of different nuts as allergen sources in different parts of the world.

**Figure 2** provides an overview of the role of different nuts as allergen sources for different regions of the world. Peanut allergy seems to be most frequent in most parts of the world whereas in Europe hazel nut allergy seems to be more important. Interestingly, different molecular IgE sensitization patterns can be observed in different geographic regions depending on birch pollen exposure involving IgE reactivity to Ara h 8, sensitization to lipid transfer proteins in southern Europe with sensitization to Ara h 9, and the classical peanut sensitization involving storage proteins such as Ara h 1, Ara h 2, Ara h 3 and Ara h 6 (94–96). In South America, nut allergy seems to be less common than in other parts of the world. Only few data are available for Africa indicating a need for further studies. It seems that early introduction of peanut in the diet as it occurs in Zimbabwe results in a low rate of symptomatic peanut allergy (52).

Notably, reports on the prevalence of nut allergies among adults are rare and most studies have been conducted in children. More studies taking into account the molecular IgE sensitization profiles and symptoms verified by highly indicative case history and/or provocation testing in children and adults are needed to obtain a more complete picture of the dominating nut allergies in different parts of the world.

## 3 Clinical Relevance of Nut Allergen Molecules

Peanut allergy is a good example for the importance of molecular diagnosis for identifying the culprit sensitizing allergen source. Patients may be allergic to peanut due to primary sensitization to birch pollen and cross-reactivity of PR-10 allergen (i.e., cross-reactivity between Bet v 1 and Ara h 8), some are sensitized to lipid transfer proteins from fruits and eventually certain pollen (e.g., cross-reactivity between Pru p 3 and Ara h 9), others may be genuinely sensitized to peanut and the corresponding peanut-specific marker allergens (Ara h 1, 2, 3 and 6) and there can be mixed sensitizations (94–96). The deconvolution of the molecular IgE sensitization profiles is therefore of high importance for identifying the genuinely sensitizing allergen source, predicting clinical manifestations (mild or severe forms of allergy), prevention and treatment based on avoidance/diet and AIT (13). New approaches for the diagnosis and therapy of nut allergies will be increasingly based on individual nut allergen molecules. The clinical relevance of different allergens significantly varies by region and age. In the overview of nut allergen molecules in **Table 2** (94, 97–161) a clear distinction has been made between cross-reactive class I food allergens, such as lipid transfer proteins, and confirmed and putative class II food allergens. Key references are given for each of the allergen molecules and reference is made to the WHO/IUIS allergen nomenclature data base (94, 97–161).

**Table 2:** Nut allergen molecules according to the WHO/IUIS allergen nomenclature (97) including information regarding biochemical, immunological and clinical features with key references.

Species	Allergen name	Protein family	Function	MW (SDS-PAGE):	Route of sensitization	Prevalence	Clinical relevance	References
***Arachis hypogaea* (peanut)**	Ara h 1	Vicilin	Seed storage protein	64 kDa	Food	Major allergen in the US, central and northern Europe	Risk of severe allergic reactions up to anaphylaxis	(94, 98–100)
	Ara h 2	2S albumin	Seed storage protein	17 kDa	Food	Major allergen in the US, central and northern Europe	Risk of severe allergic reactions up to anaphylaxis	(94, 99–104)
	Ara h 3	Legumin	Seed storage protein	60 kDa, 37 kDa (fragment)	Food	Prevalence varies between studies, but generally more prevalent in Central and North America and Northern Europe than in Mediterranean regions	Risk of severe allergic reactions up to anaphylaxis	(105)
	Ara h 5	Profilin	Actin-binding protein	15 kDa	Food	Panallergen (class II food allergy)	Pollen-food allergy syndrome	(100, 106)
	Ara h 6	2S albumin	Seed storage protein	15 kDa	Food	Reactivity usually in patients who are primarily sensitized to Ara h 2, but monosensitization possible	Risk of severe allergic reaction up to anaphylaxis	(100, 103)
	Ara h 7	2S albumin	Seed storage protein	15 kDa	Food	Reactivity usually in patients who are primarily sensitized to Ara h 2, but monosensitization possible	Predictive ability for peanut allergy similar to Ara h 2 and Ara h 6	(100, 107, 108)
	Ara h 8	PR-10 (Bet v 1-like)	Plant defense, stress mechanisms	17 kDa	Food	Panallergen (class II food allergy)	Pollen-food allergy syndrome	(94, 104, 109)
	Ara h 9	nsLTP1	Transfer of lipids across membranes, plant defense, response to environmental stress	9.8 kDa	Food	Major allergen in the Mediterranean region	Severe allergic reaction	(94, 110, 111)
	Ara h 10	Oleosin	Structural protein of oil bodies	16 kDa	Food	Not yet reported	Might be associated with severe allergic reactions	(112, 113)
	Ara h 11	Oleosin	Structural protein of oil bodies	14 kDa	Food	Not yet reported	Might be associated with severe allergic reactions	(112, 113)
	Ara h 12	Defensin	Plant defense	8 kDa (reducing), 12 kDa (non-reducing), 5.184 kDa (mass)	Food	Not yet reported	Might be associated with severe allergic reactions	(114)
	Ara h 13	Defensin	Plant defense	8 kDa (reducing), 11 kDa (non-reducing), 5.472 kDa (mass)	Food	Not yet reported	Might be associated with severe allergic reactions	(114)
	Ara h 14	Oleosin	Structural protein of oil bodies	17.5 kDa	Food	Not yet reported	Might be associated with severe allergic reactions	(112, 113)
	Ara h 15	Oleosin	Structural protein of oil bodies	17 kDa	Food	Not yet reported	Might be associated with severe allergic reactions	(112, 113)
	Ara h 16	nsLTP2	Transfer of lipids across membranes, plant defense, environmental stress	8.5 kDa by SDS-PAGE reducing	Food	Not yet reported	Not yet reported	(97)
	Ara h 17	nsLTP1	Transfer of lipids across membranes, plant defense, environmental stress	11 kDa by SDS-Page reducing	Food	Not yet reported	Not yet reported	(97)
	Ara h 18	Cyclophilin	Peptidyl-prolyl cis-trans isomerase	21 kDa	Food	Not yet reported	Not yet reported	(97)
***Juglans regia* (English walnut)**	Jug r 1	2S albumin	Seed storage protein	15-16 kDa	Food	Major allergen in the US	Risk of severe allergic reactions	(115, 116)
	Jug r 2	Vicilin	Seed storage protein	44 kDa	Food	Major allergen in the US	Risk of severe allergic reactions up to anaphylaxis	(117, 118)
	Jug r 3	nsLTP	Transfer of lipids across membranes, plant defense, response to environmental stress	9 kDa	Food	Major allergen in the Mediterranean region	Severe allergic reactions	(118)
	Jug r 4	Legumin	Seed storage protein	58.1 kDa	Food	Major allergen in patients with objective symptoms	Risk of severe allergic reactions up to anaphylaxis	(119, 120)
	Jug r 5	PR-10 (Bet v 1-like)	Plant defense, stress mechanisms	20 kDa	Food	Panallergen (class II food allergen)	Pollen-food allergy syndrome	(121)
	Jug r 6	Vicilin	Seed storage protein	47 kDa	Food	Minor allergen	Might be associated with severe allergic reactions	(122)
	Jug r 7	Profilin	Actin-binding protein	13 kDa	Food	Panallergen (class II food allergen)	Pollen-food allergy syndrome	(97)
	Jug r 8	nsLTP2	Transfer of lipids across membranes, plant defense, response to environmental stress	9 kDa	Food	Not yet reported	Not yet reported	(97)
***Juglans nigra* (black walnut)**	Jug n 1	2S albumin	Seed storage protein		Food	Not yet reported	Not yet reported	(97)
	Jug n 2	Vicilin	Seed storage protein		Food	Not yet reported	Not yet reported	(97)
	Jug n 4	Legumin	Seed storage protein	34 kDa, 22 kDa	Food	Not yet reported	Not yet reported	(123)
***Corylus avellana* (hazel)**	Cor a 1: Cor a 1.01 major hazel pollen allergen; Cor a 1.04 major allergen in hazelnut	PR-10 (Bet v 1-like)	Plant defense, stress mechanisms	17 kDa	Airway, Food (seed and pollen)	Panallergen (class II food allergy)	Pollen-food allergy syndrome	(124–126)
	Cor a 2	Profilin	Actin-binding protein	14 kDa	Airway, Food (seed and pollen)	Panallergen (class II food allergy)	Pollen-food allergy syndrome	(127)
	Cor a 6	Isoflavone reductase homologue		35 kDa	Airway (pollen)	Not yet reported	Not yet reported	(97)
	Cor a 8	nsLTP	Transfer of lipids across membranes, plant defense, response to environmental stress	9 kDa	Food	Major allergen in the Mediterranean region; might also be associated with severe allergy in birch-endemic regions	Risk of severe allergic reactions	(124, 128–130)
	Cor a 9	Legumin	Seed storage protein	40 kDa	Food	Major allergen in patients with objective symptoms unrelated to pollen; predominantly in children	Risk of severe allergic reactions	(131, 132)
	Cor a 10	Luminal binding protein		70 kDa	Airway (pollen)	Not yet reported	Not yet reported	(133)
	Cor a 11	Vicilin	Seed storage protein	48 kDa	Food	Minor allergen, predominantly in children	Might be associated with severe allergic reactions	(132, 134, 135)
	Cor a 12	Oleosin	Structural protein of oil bodies	17 kDa	Food	Not yet reported	Might be associated with severe allergic reactions	(136)
	Cor a 13	Oleosin	Structural protein of oil bodies	14-16 kDa	Food	Not yet reported	Might be associated with severe allergic reactions	(136)
	Cor a 14	2S albumin	Seed storage protein	10 kDa reducing	Food	Major relevance in patients with severe allergy unrelated to pollen; predominantly in children	Risk of severe allergic reactions	(137–139)
	Cor a 15	Oleosin	Structural protein of oil bodies	17 kDa	Food	Not yet reported	Might be associated with severe allergic reactions	(136)
***Pistacia vera* (pistachio)**	Pis v 1	2S albumin	Seed storage protein	7 kDa	Food	Major allergen	Not defined on single molecule level; pistachio allergy can lead to severe allergic reactions	(140)
	Pis v 2	Legumin	Seed storage protein	32 kDa	Food	Major allergen	Not defined on single molecule level; pistachio allergy can lead to severe allergic reactions	(140)
	Pis v 3	Vicilin	Seed storage protein	55 kDa	Food	Minor allergen	Not defined on single molecule level; pistachio allergy can lead to severe allergic reactions	(141)
	Pis v 4	Manganese superoxide dismutase	Prevention of oxidative damage	25.7 kDa	Food	Major allergen in study by Ayuso et al.; minor allergen in study by Noorbakhsh et al.	Not defined on single molecule level; pistachio allergy can lead to severe allergic reactions	(142, 143)
	Pis v 5	Legumin	Seed storage protein	36 kDa (acidic subunit)	Food	Minor allergen according to Willison et al. (referring to unpublished data)	Not defined on single molecule level; pistachio allergy can lead to severe allergic reactions	(97, 144)
***Anacardium occidentale* (cashew)**	Ana o 1	Vicilin	Seed storage protein	50 kDa	Food	Major allergen	Not defined on single molecule level; cashews are associated with severe allergic reactions	(145)
	Ana o 2	Legumin	Seed storage protein	55 kDa	Food	Major allergen	Not defined on single molecule level; cashews are associated with severe allergic reactions	(146)
	Ana o 3	2S albumin	Seed storage protein	14 kDa	Food	Major allergen	Not defined on single molecule level; cashews are associated with severe allergic reactions	(147)
***Prunus dulcis* (almond)**	Pru du 3	nsLTP1	Transfer of lipids across membranes, plant defense, response to environmental stress	9 kDa	Food	LTPs usually prevalent in Mediterranean region	Might lead to severe allergic reactions, based on allergenicity of other nsLTPs	(97)
	Pru du 4	Profilin	Actin-binding protein	14 kDa	Airway (pollen)	Panallergen (class II food allergy)	Pollen-food allergy syndrome	(148)
	Pru du 5	60s acidic ribosomal protein. P2		10 kDa	Airway (pollen)	Possibly major allergen, but more studies needed	Not yet reported	(149)
	Pru du 6	Legumin	Seed storage protein	60 kDa (360 kDa hexamer)	Food	Major allergen	Might be a specific marker for almond allergy	(150–152)
	Pru du 8	Antimicrobial seed storage protein	Seed storage protein	31 kDa	Food	Not yet reported	Not yet reported	(153)
	Pru du 10	Mandelonitrile lyase 2		60 kDa	Food	Not yet reported	Not yet reported	(97)
***Bertholletia excelsa* (Brazil nut)**	Ber e 1	2S albumin	Seed storage protein	9 kDa	Food	Major allergen	Risk of severe allergic reactions up to anaphylaxis	(154, 155)
	Ber e 2	Legumin	Seed storage protein	29 kDa	Food	Major allergen	More studies needed for clinical evaluation	(156, 157)
***Carya illinoinensis* (pecan)**	Car i 1	2S albumin	Seed storage protein	16 kDa	Food	Major allergen	More studies on single-molecule level needed for clinical evaluation	(158)
	Car i 2	Vicilin	Seed storage protein	55 kDa	Food	Minor allergen	More studies on single-molecule level needed for clinical evaluation	(159)
	Car i 4	Legumin	Seed storage protein	Subunit of hexameric protein: 55.4 kDa	Food	Major allergen	More studies on single-molecule level needed for clinical evaluation	(160)
***Macadamia integrifolia* (macadamia)**	Mac i 1	Vicilin	Seed storage protein	50 kDa	Food	Not yet reported	More studies on single-molecule level needed for clinical evaluation	(97)
	Mac i 2	Legumin	Seed storage protein	60 kDa non reducing; 20 kDa and 40 kDa reducing	Food	Not yet reported	More studies on single-molecule level needed for clinical evaluation	(97)

Confirmed (light blue) and putative (dark blue) cross-reactive class II allergens are highlighted.

### 3.1 Overview of Source-Related Nut Allergen Molecules

#### 3.1.1 Peanut

At present, 17 peanut (*Arachis hypogaea*) allergens – Ara h 1 to Ara h 18 – have been identified, with exception of Ara h 4 which was identified as isoform of Ara h 3 (97) (**Table 2**). Peanut allergens belong either to the prolamin superfamily (Ara h 2, Ara h 6, Ara h 7, Ara h 9, Ara h 16, Ara h 17), the cupin superfamily (Ara h 1, Ara h 3) or different other proteins such as profilin (Ara h 5), Bet v 1-like (Ara h 8), oleosins (Ara h 10, Ara h 11, Ara h 14, Ara h 15) or defensins (Ara h 12, Ara h 13) (97). Recently, the cyclophilin-peptidyl-prolyl cis-trans isomerase Ara h 18 was officially recognized as peanut allergen by the WHO/IUIS Allergen Nomenclature Sub-committee (97).

In America, Central and Northern Europe, Ara h 1 and Ara h 2 are major peanut allergens (94, 99). Valcour et al. showed that in the US, patients with reported peanut allergy most frequently recognized Ara h 2 but IgE reactivity to Ara h 1 and Ara h 3 was also highly prevalent in the tested patients (104). Kleber-Janke et al. reported IgE reactivity to Ara h 1 in 65% and to Ara h 2 in 85% of sera from patients (n = 40) with reported peanut allergy (100). Koppelman et al. compared the IgE reactivity of 32 peanut-allergic patients to Ara h 1, Ara h 2 and Ara h 3 and showed that of these three allergens, Ara h 2 was most frequently recognized (26/32) (102). Importantly, sensitization to Ara h 2 is associated with severe allergic reactions (103). Ara h 2 further has the potential to cross-react with other 2S albumins such as Ara h 6 and Ara h 7, with Ara h 2 possibly representing the primary sensitizing agent (108, 162). However, in rare cases, monosensitization to Ara h 6 and Ara h 7 might be observed and thus must be considered for accurate diagnosis (108, 163). It has been shown that detection of IgE reactivity to peanut extract together with reactivity to rAra h 2 and rAra h 6 allows reliable peanut allergy diagnosis and Ara h 2 could significantly increase diagnostic specificity (164). In comparison to Ara h 1 and Ara h 2, sensitization to Ara h 3 is less frequently observed (94, 102, 105).

In the Mediterranean region, sensitization to the nsLTP Ara h 9 is common and has high cross-reactive potential with homologous allergens of the *Rosaceae* family, in particular the peach nsLTP Pru p 3 (94, 110, 111, 165).

Schwager et al. reported sensitization to peanut oleosins in patients with a history of severe allergic reactions (113). According to the authors, roasting of peanuts seemed to increase the IgE-binding capacity of oleosins. Previously, several studies have reported that roasting might enhance the allergenic activity of peanut allergens (166–169).

So far, little is known regarding the clinical relevance of peanut defensins and the nsLTPs Ara h 16 and Ara h 17 as well as the currently approved cyclophilin-peptidyl-prolyl cis-trans isomerase Ara h 18 which may be cross-reactive with corresponding pollen and respiratory allergens.

#### 3.1.2 Walnut

For the English walnut (*Juglans regia*), which belongs to the *Juglandaceae* family, 8 allergens have been officially approved by the allergen nomenclature (Jug r 1 to 8), making it the clinically most relevant walnut species (97, 116) (**Table 2**). For the black walnut (*Juglans nigra*) 3 allergens have been identified (Jug n 1, 2, 4) (97). However, their clinical relevance is not yet well described in the literature.

Teuber et al. reported that 12 out of 16 walnut-allergic patients showed IgE reactivity to a 2S albumin from English walnut, designated Jug r 1, thus identifying it as a major walnut allergen (115).

IgE reactivity to another major walnut allergen, the vicilin Jug r 2, was detected in 9 out of 15 patients from the US (117). In a study by Pastorello et al., IgE reactivity to vicilin-like protein precursors and vicilin precursors of 9 kD was observed in 10 out of 46 sera from Italian patients, suggesting a minor role of vicilins in allergic patients in the Mediterranean region (118).

Pastorello et al. further reported that 37 out of 46 sera showed IgE binding to the walnut nsLTP Jug r 3, leading to the conclusion that in southern Europe, Jug r 3 represents a major allergen of walnut (118). Notably, peach LTP (Pru p 3) completely inhibited IgE binding to Jug r 3, indicating strong cross-reactivity between walnut and peach.

In 2003, Teuber et al. observed IgE sensitization of patients who experienced life-threatening systemic reactions after walnut consumption to a walnut protein of the legumin group, designated Jug r 4 (119). IgE binding to a recombinant Jug r 4 fusion protein was observed in 15 out of 23 tested sera, suggesting major importance of Jug r 4 in patients with confirmed symptoms. Another study showed IgE reactivity to recombinant Jug r 4 in 21 out of 37 sera from walnut-allergic patients (120).

Jug r 6, like Jug r 2 and Jug r 4, is a member of the cupin superfamily. Although Jug r 2 and Jug r 6 belong to the same protein family, they share only 44% identity (122). In comparison to Jug r 2, which was identified as a major walnut allergen by Teuber et al., Jug r 6 showed IgE reactivity in 20 of 77 walnut-allergic patients, indicating it is of minor clinical relevance (117, 122). Interestingly, cross-reactivity has been shown between Jug r 6 and homologues from pistachio, sesame and hazelnut, which, however, did not apply for Jug r 2 (122).

#### 3.1.3 Hazelnut

So far, 11 allergens from common hazel (*Corylus avellana*) are registered in the WHO/IUIS database (97) (**Table 2**).

Sensitization to the nsLTP, Cor a 8 predominantly occurs in patients from the Mediterranean region and has been associated with severe allergic reactions (128, 130). However, also in birch-endemic regions, sensitization to Cor a 8 was found in children who had objective reactions during DBPCFC (129). Pastorello et al. reported IgE reactivity to Cor a 8 in patients with a history of anaphylactic reactions to hazelnuts and demonstrated inhibition of IgE binding to Cor a 8 by the purified Pru p 3 (124).

Severe allergic reactions unrelated to pollen allergy have also been reported from patients with sensitization to the 11S globulin Cor a 9 and the 7S globulin Cor a 11 (132). IgE reactivity to Cor a 9 was detected in 12 of 14 patients with a history of systemic reactions to hazelnuts (131). In hazelnut-allergic patients from birch-endemic regions, age-related differences regarding the sensitization to Cor a 9 were observed (126). In total, 65% of pre-school children and 50% of schoolchildren, but only 17% of adults with systemic reactions were sensitized to Cor a 9. In a study by Lauer et al., IgE sensitization to Cor a 11 was observed in less than 50% of 65 hazelnut-allergic patients and the allergen demonstrated significantly lower biological activity in comparison to Cor a 1, suggesting that Cor a 11 is a less relevant hazelnut allergen (134). Similar to Cor a 9, in birch-endemic regions, sensitization to Cor a 11 is age-dependent and is recognized predominantly by children with objective symptoms (135).

The 2S albumin Cor a 14 was first identified in 2010 (137). In a study by Faber et al., IgE reactivity of hazelnut-allergic patients to Cor a 14 was analyzed in different age groups, revealing that Cor a 14 was predominantly recognized in pre-school (18/20) and school-aged children (8/10) (139). In Dutch patients with hazelnut allergy, sensitization to Cor a 14 and Cor a 9 was shown to be highly specific for predicting more severe hazelnut allergy (138). Similar results were obtained in another study that examined the role of component resolved diagnostics for the prediction of clinical allergy in hazelnut-allergic children (170). Specific IgE to Cor a 14 was found to be reliable for the discrimination between patients with clinical reactivity and those that were nonreactive.

The hazelnut oleosins Cor a 12, Cor a 13 and Cor a 15 might be associated with severe allergic reactions (136, 171). However, more studies are needed to establish their clinical relevance. In Europe, sensitization to Cor a 12 in patients with reported reactions to hazelnuts ranged from 10 to 25% and appeared to be more frequent in children than adults (172). The clinical relevance of Cor a 6, a isoflavone reductase-related protein, and Cor a 10 a luminal binding protein with possible pollen cross-reactivity remains to be determined.

#### 3.1.4 Pistachio

Five allergens from *Pistacia vera* (Pis v 1, Pis v 2, Pis v 3, Pis v 4 and Pis v 5) have been officially approved (97) (**Table 2**). The sensitization profile of patients with pistachio allergy varies significantly across Europe, indicating age-related, demographic and ethnic differences among the population (56, 60, 63). The clinical relevance of individual pistachio allergens has not been investigated in detail, but it has been shown that pistachio allergy can lead to severe allergic reactions (173).

Ahn et al. reported IgE reactivity in the serum of 19 out of 28 pistachio-allergic patients to a 7 kDa 2S albumin, which was designated Pis v 1. Moreover, 14 out of 28 patients showed IgE binding to the legumin-like protein Pis v 2 (140). These allergens were further identified as homologous of the cashew allergens Ana o 3 and Ana o 2, respectively. The cashew tree belongs just like pistachio to the *Anacardiaceae* family, which explains the high structural similarity of the proteins and indicates cross-reactivity.

IgE sensitization to the 7S globulin Pis v 3 was shown in 7 of 19 patients who had a history of allergic reactions to pistachio and/or cashew (141). The patients with IgE reactivity to rPis v 3 also reacted to rAna o 1 from cashew nut.

In 16 out of 27 sera from pistachio-allergic patients, IgE reactivity to a manganese superoxide dismutase (MnSOD)-like protein, designated Pis v 4, from pistachio was detected (142). MnSOD-like proteins are known as cross-reactive respiratory allergens (174) and hence Pis v 4 may be considered as a class II food allergen. In 2010, Noorbakhsh et al. reported the expression and purification of recombinant Pis v 4, which exhibited IgE reactivity in 10 of 25 patients (143). Moreover, cross-reactivity with other MnSODs was suggested by the authors.

Pis v 5 is another legumin of pistachio nut, but little is known about the clinical relevance of this protein (97). However, it was described as minor pistachio allergen by Willison et al., referring to unpublished data that reported IgE reactivity in 10 out of 28 patients (144).

#### 3.1.5 Cashew

Currently, three cashew (*Anacardium occidentale*) allergens are registered in the database of the WHO/IUIS (97) (**Table 2**). The vicilin Ana o 1, the legumin Ana o 2 and the 2S albumin Ana o 3 are listed as the major allergens of cashew nut.

Wang et al. reported IgE reactivity to rAna o 1 in 10 out of 20 patients with a history of severe reactions to cashew (145). IgE reactivity to rAna o 2 was shown in 13 out of 21 cashew-allergic patients (146). Robotham et al. detected IgE reactivity to rAna o 3 in 21 of 26 patients with cashew nut allergy (147). Cross-reactivity between the botanically related cashew and pistachio nuts, both members of the *Anacardiaceae* family, has been observed in several studies (64, 141, 175).

#### 3.1.6 Almond

So far 6 allergens from *Prunus dulcis* (Pru du 3, Pru du 4, Pru du 5, Pru du 6, Pru du 8 and Pru du 10) have been officially recognized by the WHO/IUIS (97) (**Table 2**).

Pru du 3 belongs to the nsLTP family, which is usually associated with high allergenic activity and cross-reactivity between members of the *Rosaceae* family, mainly in the Mediterranean region (176, 177). However, large clinical studies evaluating the prevalence of IgE sensitization to Pru du 3 in almond-allergic patients from different regions are needed.

The 60s acidic ribosomal protein P2 has been identified as Pru du 5, and IgE reactivity to a recombinant variant of the protein was shown in 4 of 8 almond-sensitized patients (149). Acid ribosomal proteins have been identified in molds as allergens and it may therefore be considered that this allergen may represent a class II food allergen (178).

Reactivity to recombinant variants of the amandin Pru du 6, Pru du 6.01 and Pru du 6.02, was seen in 9 of 18 and 5 of 18 almond-allergic patients, respectively, while only 4 of the tested patients showed IgE reactivity to both isoforms (151). Kabasser et al. suggested that Pru du 6 might be a specific marker for almond allergy since 16 of 18 almond-allergic patients showed IgE reactivity to the allergen (152). Moreover, positive sIgE to Pru du 6 provided a specificity of 78% and a sensitivity of 83% for almond allergy, while at the same threshold level, the detection of sIgE to almond extract significantly lacked specificity (33%). In comparison, Pru du 8 and Pru du 10 had specificities of 100% and 61% but were less sensitive (41% and 67%) (152). The antigenicity of almond amandin does not seem to be influenced by roasting, blanching or autoclaving, indicating high protein stability (179, 180).

In 2019, Che et al. reported that Pru du 8 might be a member of a novel food allergen family with antimicrobial properties and demonstrated IgE reactivity against rPru du 8 in 6 of 18 patients (153).

#### 3.1.7 Brazil Nut

To this date, the 2S albumin Ber e 1 and the 11S globulin Ber e 2 from Brazil nut (*Bertholletia excelsa*) have been registered in the allergen data base (97) (**Table 2**).

Pastorello et al. reported that each of 11 patients with a history of anaphylaxis after the consumption of Brazil nut, showed IgE reactivity to a 2S albumin, implying that it represents a major allergen from Brazil nut (154). Rayes et al. suggested improvement of allergy diagnosis by measurement of IgE to recombinant Ber e 1, which provides higher sensitivity without loss of specificity compared to whole nut extract (181). Beyer et al. reported the identification of a 11S globulin, designated Ber e 2, as another major allergen from Brazil nut, showing IgE reactivity to the native protein in 56% and the recombinant variant in 44% of sera from Brazil nut-sensitized patients (n = 27) (157).

#### 3.1.8 Pecan

Three proteins from *Carya illinoinensis*, the 2S albumin Car i 1, the vicilin Car i 2 and the legumin Car i 4 have been officially approved as allergens (97) (**Table 2**).

In 2011, the 2S albumin Car i 1 was characterized and IgE binding to recombinant Car i 1 was detected in 22 of 28 patients with pecan allergy (158). The same study showed that pecan and walnut extracts inhibited IgE binding to recombinant Car i 1, indicating strong cross-reactivity with homologous proteins from these nuts. In 2016, Zhang et al. reported that 6 out of 25 patients with DBPCFC-confirmed pecan allergy, showed IgE reactivity to pecan vicilin Car i 2 (159). In a study by Sharma et al., an 11S globulin from pecan, designated Cari i 4, was recognized by IgE from 16 out of 28 patients with pecan allergy (160). Furthermore, extracts from pecan as well as walnut inhibited IgE binding to rCar i 4, suggesting cross-reactivity with legumins from other tree nuts.

#### 3.1.9 Macadamia

To date, 2 allergens from macadamia nut (*Macadamia integrifolia*), the vicilin Mac i 1 and the legumin Mac i 2, are included in the allergen list of the WHO/IUIS Allergen Nomenclature Sub-committee (97) (**Table 2**).

In a study by Sutherland et al., IgE reactivity to a 17.4 kDa protein from macadamia was shown in the serum of a patient that had experienced anaphylaxis after consumption of a cake made with macadamia meal (182). Herbst et al. reported IgE reactivity to a macadamia protein of 45 kDa and, under non-reducing conditions, to another protein of 12 kDa (183). Recently, Ehlers et al. reported IgE recognition of vicilin-like antimicrobial peptides in 24 of 82 nut-allergic patients, including 3 patients with a history of systemic reactions to macadamia nut (184). According to available data, measurement of specific IgE to macadamia nut does not always predict clinical allergy and might lead to false-negative results (185, 186). However, single allergen molecules of macadamia nut for component resolved diagnosis are lacking and it must be considered that macadamia extracts might not contain all relevant allergens and thus provide low diagnostic sensitivity (186). Therefore, the identification and characterization of macadamia proteins with established allergenic potential is urgently needed. Possible cross-reactivity between macadamia and hazelnut has been suggested (182, 183).

### 3.2 Clinically Relevant Panallergens to Be Considered as Class II Food Allergens

In peanuts, one of the most relevant panallergens is the Bet v 1-like homologue Ara h 8, which is of major importance in patients from birch-endemic regions where allergic reactions to peanuts can be strongly associated with sensitization to birch pollen (94, 104, 109). Similarly, IgE reactivity to the profilin Ara h 5 is associated with previous sensitization to pollen (106). In walnut, the pathogenesis-related protein (PR-10) Jug r 5 is associated with IgE cross-reactivity between homologous allergens from different plant sources and of minor relevance for patients with primary walnut allergy (121). The Bet v 1-like Cor a 1 and the profilin Cor a 2 are cross-reactive allergens of hazelnut and sensitization to these allergens is typically seen in birch-endemic regions (50, 125, 127). Both allergens are expressed in hazelnut as well as in hazel pollen. The profilin Pru du 4 is a minor allergen of almond and cross-reactivity with profilins from grass pollen was reported (148). It is quite likely that additional “food allergens” (**Table 2**, light blue) will be identified for which sensitization occurs by respiratory allergen sources and symptoms of food allergy will be low because the allergens are not heat stable and/or become easily digested and then lose their allergenic activity. Ara h 18, Cor a 6, Pis v 4 and Pru du 5 are possible candidates and there may be more discovered in the future (**Table 2**, dark blue). IgE reactivity to the class II nut allergens is not due to genuine nut sensitization and symptoms caused by these allergens may be treated by AIT directed to the originally sensitizing respiratory allergens.

## 4 Diagnosis of Nut Allergy

**Figure 3:**
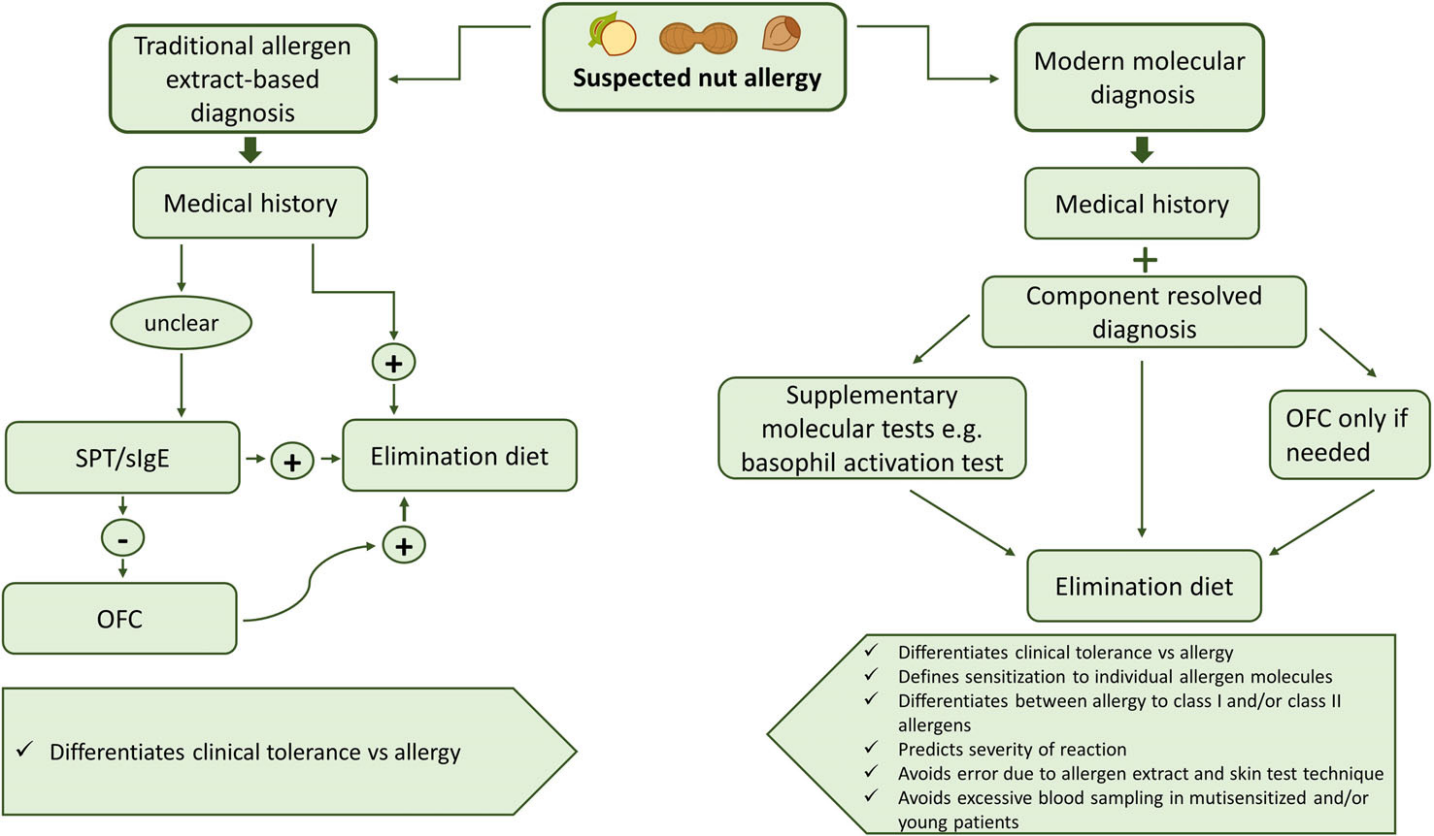
Overview of traditional allergen extract-based nut allergy diagnosis in comparison with modern molecular diagnosis.

Diagnosis of nut allergies usually starts with the evaluation of the medical history of the patient. While in the past, diagnosis was mainly achieved by allergen extract-based tests (SPT, OFC), these are increasingly being replaced by modern molecular techniques using specific allergen molecules (**Figure 3**) (187). **Figure 3** compares traditional allergen extract-based diagnosis for nut allergy with modern molecular allergy diagnosis. Traditional extract-based diagnosis uses allergen extracts prepared from the allergen sources for serology and provocation testing in conjunction with the clinical history to determine food which can elicit allergic reactions. Molecular allergy diagnosis is based on IgE serology to a broad panel of defined allergen molecules in combination with the clinical history. In this pathway provocation testing is reduced and usually only performed if necessary to confirm clinically relevant allergy if this cannot be determined by molecular testing and medical history. Molecular testing offers high precision regarding the identification of the culprit allergen molecules is fast and helps to reduce provocation testing which can give rise to severe reactions (187).

### 4.1 Food Challenges

Double-blind, placebo-controlled oral food challenge is still a common procedure for food allergy diagnosis, although in the case of strong clinical suspicion, this is usually avoided. Generally, it is recommended that DBPCFC is performed in a standardized procedure under consideration of several patient-related and procedure-related parameters (188, 189). Nevertheless, it must be taken into account that oral food challenges (OFC) bear the risk of potentially fatal anaphylaxis during the procedure (43). This applies particularly to nuts, which are among the most common foods causing anaphylaxis (5). In recent studies, lip dose challenges (LDC), using fresh nuts or nut paste, were suggested as a supplement for oral challenges for nut allergy diagnosis (44, 190). LDC might be performed as a preliminary test to an OFC but currently cannot replace the latter. However, LDC, in combination with modern molecular diagnostic, might reduce the need for OFC in the future.

### 4.2 Skin Tests

In principle, two types of skin tests can be performed for diagnostics purposes. Skin prick testing measures the induction of mast cell degranulation caused by cross-linking of IgE bound to the high affinity IgE receptor (FcϵRI) (191) whereas atopy patch testing (APT) detects allergen-specific T cell activation even in the absence of IgE-mediated effects (191, 192). Accordingly, SPT may be considered as surrogate test for IgE-mediated immediate allergic inflammation and APT as surrogate test for chronic, T cell-mediated allergic inflammation. SPT and the detection of food-specific serum IgE with allergen extracts have been traditionally used for allergy diagnosis but have major weaknesses. First of all, these tests are performed with poorly defined allergen extracts and hence do not identify the sensitizing allergen molecules (193). Second, both methods cannot be used to predict clinical sensitivity with certainty because the extent to which digestion affects allergenic activity cannot be measured with these methods. Several authors suggested that the use of fresh food might increase test sensitivity (194, 195). Therefore, food challenge tests are still recommended despite the associated risk factors.

### 4.3 Molecular Allergy Diagnosis

Molecular allergy diagnosis is based on the use of purified allergen molecules, mainly recombinant allergens, to determine the IgE sensitization profile of allergic patients (45). There are also attempts to improve the diagnosis of nut allergy by combining different forms of allergen extracts-based diagnosis. For example, it has been shown that prediction of clinical reactivity to pistachio and cashew was improved by SPT in combination with measurement of sIgE (196). However, nowadays native purified or recombinant single allergen molecules are increasingly replacing conventional extracts in *in vitro* diagnostics. Molecular tests that allow the detection of specific IgE antibodies to individual allergen molecules are also known under the term component-resolved diagnostics (CRD) (197). For peanut allergy, it was demonstrated that by measuring Ara h 2-specific IgE, the diagnostic accuracy could be considerably improved (198–201). When measured together, sIgE reactivity to Ara h 6 and Ara h 2 was shown to be predictive for severe peanut allergy (103). For the prediction of positive outcomes of food challenges in children, it was demonstrated that Ara h 2-specific IgE levels of 14.4 kU_A_/L and Cor a 14-specific IgE levels of 47.8 kU_A_/L had an estimated probability of 90% for predicting a positive peanut or hazelnut challenge (202). In another study, Cor a 14-specific IgE levels of 0.5 and 1.0 kU_A_/L had a probability of 50% and 95% to predict clinical reactivity to hazelnut in sensitized patients, respectively (170). Moreover, it was shown that measurement of sIgE levels for Cor a 9 in hazelnut-sensitized patients might improve the diagnostic accuracy for the prediction of hazelnut allergy in Japanese children (203). For cashew it was found that sIgE to individual allergen molecules from cashew nut had a predictive value for the diagnosis of clinical allergy (204–206). Measurement of Jug r 1-specific IgE was suggested for the prediction of walnut allergy in children due to improved clinical specificity in comparison with IgE to walnut extracts (207).

Several assays have been developed for the detection of serum IgE to either a single allergen analyte (singleplex assay) or various allergens at a time (multiplex assay) (187, 208, 209). The availabilities of single allergens and advanced microarray technology have made it possible to obtain a quick insight into the sensitization profile of a patient (210). In order to enable quantitative conversion between different multiplex IgE test-platforms for nut allergens, statistical models have been established recently (211). For the European MeDALL research project, an allergen chip with 170 allergen molecules, including natural purified and recombinant allergens from almond, cashew, pistachio and peanut, was developed which could be used even for dried blood samples (212). Recently, a study showed moderate agreement of microarray-based analysis in comparison with clinical diagnosis but high sensitivity of the microarray was seen for tree nuts (213). Moreover, the microarray results for tree nuts correlated with SPT results, promising a superior role of component resolved diagnostic for nut allergies in the future.

Another interesting approach for *in vitro* allergy diagnosis of nut allergy is the basophil activation test (BAT). Since the early description of allergen-induced histamine release from basophils (214) and the demonstration of the applicability of basophil activation testing for recombinant allergens (215), basophil activation testing has continuously developed (216). Importantly, basophil activation can discriminate between IgE-reactive antigens with no or poor ability to induce IgE-mediated receptor aggregation from potent allergens which induce basophil activation already at low doses (32, 217). Thus basophil activation testing is useful to address a major problem of *in vitro* allergy diagnostics, i.e., the possibility of false-positive results due to the presence of cross-reactive carbohydrate determinants (218). In plants, these IgE-binding carbohydrate structures are usually N-glycans with a core α-1,3-linked fucose residue. It is well established that CCDs are responsible for IgE cross-reactivity between a wide range of plant allergens and other unrelated allergen sources (219). Furthermore, the presence of N-glycans in cellulose-based ImmunoCap assays could lead to false-positive results in patients with high levels of CCD-reactive IgE antibodies (220). Possibilities to overcome IgE reactivity to CCDs are the production of non-glycosylated recombinant allergen molecules or the use of specific CCD inhibitors (221). CCD-directed IgE antibodies seem to have poor biological activity and are not associated with clinical symptoms (222–224). In basophil activation tests, flow cytometry can be used to analyze basophil activation, which, for example can be defined by the upregulation of the lineage-specific basophil marker CD203c together with the degranulation marker CD63 (225) as has been shown for hazelnut allergy (226). Alternatively, rat basophil cell lines transfected with human FcϵRI can be loaded with serum IgE and then stimulated with allergens (227). Basophil activation was found useful for predicting clinical reactions in peanut allergic patients. Glaumann et al. reported that negative basophil allergen threshold sensitivity correlated with negative DBPCFC in children with peanut allergy (228). Moreover, 92% with positive DBPCFC had positive threshold sensitivity results and increased levels of IgE antibodies to the major peanut allergens Ara h 1, Ara h 2 and Ara h 3. More recently, basophil activation testing was reported to have high accuracy for the diagnosis of peanut and tree nut allergy but it has not been studied if it can be used to differentiate between sensitization to class I and class II food allergens, causing mild and severe systemic anaphylactic reactions, respectively (229).

Basophil activation testing is also a useful tool to investigate the efficacy of AIT for nut allergy by demonstrating the ability of allergen-specific immunoglobulin G (IgG) antibodies to block IgE-mediated immediate allergic reactions (230, 231).

## 5 Allergen-Specific Immunotherapies for Nut Allergies

Most of the strategies for treatment and prevention of food allergy and in particular of nut allergy (e.g., allergen avoidance, diet, use of hypoallergenic food products, AIT) are tightly connected with the accurate identification of the culprit allergens. However, some measures like the management of severe acute and chronic inflammation may be achieved by drugs such as epinephrine injection for treatment of acute anaphylactic reactions, immunosuppressive drugs and anti-IgE treatment (232). Besides diet, AIT is the most important form of allergen-specific treatment. The immunological mechanisms underlying AIT include a modified allergen-specific antibody, cellular and cytokine response (233). Besides complex alterations of the cellular and cytokine responses it has become clear that the induction of allergen-specific IgG and perhaps of allergen-specific IgA antibodies which block IgE binding to the allergen and accordingly the IgE antibody-mediate pathology is a key mechanism of AIT (234–236). This has been evidenced in clinical studies using molecular approaches for AIT (237, 238) and by the demonstration that passive immunization with allergen-specific blocking IgG antibodies is clinically effective (239–241).

### 5.1 Current Forms of AIT For Nut Allergy Are Mainly Based on Allergen Extracts and Subcutaneous AIT Is Rarely Used

Regarding the treatment of respiratory allergy by AIT subcutaneous injection immunotherapy remains to be the most frequently used and effective form of AIT as documented by a large number of clinical studies although a huge effort has been done to promote sublingual immunotherapy (SLIT) in multiple studies (235, 242). However, SCIT is more effective than SLIT and patients adherence to SCIT is much better than to SLIT (235, 243). Regarding AIT of food allergy it is of note, that there are only few early studies regarding SCIT (244, 245) and it seems that due to unfavorable side effect profiles SCIT has not been further pursued for food allergy. Instead, oral immunotherapy (OIT) has been developed for class I food allergens which are resistant to digestion whereas OIT studies for respiratory allergens and class II food allergens which are sensitive to digestion have not been successful (246–248). Another important aspect is that only few attempts were made to introduce molecular forms of AIT for food allergy whereas different forms of molecular AIT have been evaluated for respiratory allergy (235). One possible reason for this could be that many more patients suffer from respiratory allergy than from food allergy and usually new forms of treatment are mainly evaluated for frequently occurring forms of allergy because the costs for the preclinical and clinical development of novel vaccines are high. Accordingly, the majority of AIT trials for food allergy have been performed with allergen extracts and by using the OIT approach.

### 5.2 Oral Immunotherapy

OIT is based on the controlled ingestion of the allergen-causing food, intending to achieve sustained desensitization in the patients. It has been shown that similar as for SCIT, the success of treatment is associated with the development of allergen-specific IgG blocking antibodies which have actually been measured in many of the OIT studies. **Table 3** provides and overview of OIT studies (249–279) informing about the number of participants, the study design, clinical and immunological outcomes, side effects and references and/or trial registration numbers which allow to track the studies in the Clinical Trials data base (https://clinicaltrials.gov/). Most of the studies were conducted for peanut allergy whereas OIT studies for tree nut allergies are scarce (**Table 3**). A study by Andorf et al. (280) is one of the few studies providing evidence for effects of OIT to several different nuts when OIT was combined with anti-IgE treatment.

**Table 3:** Overview of clinical studies performed for peanut and tree nut allergy grouped according to the route of administration (OIT, SLIT, EPIT, rectal application).

Allergen	Number of participant (age)	Study design	Protocol summary	Clinical outcome	Serological outcome	Reported side effects	Clinical trial number	Ref.
**OIT**								
**Peanut:** peanut flour (50% protein); for additional analysis peanut proteins were extracted from peanut flour, Ara h 2 was purified and protein concentrations were determined by bicinchoninic acid assay	29 subjects (1-16 years) completed the 3 phases of the study and OFC	Open-label	Initial dose escalation day starting at 0.1 mg peanut protein. Dose was doubled every 30 minutes up to 50 mg. Build-up phase started with highest tolerated dose during initial day escalation. During build-up phase daily ingestion of peanut protein with biweekly dose increases (by 25 mg) until 300 mg reached. For patients that stopped initial escalation dosing below 50 mg, doses were doubled every 2 weeks until 50 mg reached, followed by increases of 25 mg. After reaching a daily tolerated dose of 300 mg peanut protein, dose was maintained until OFC. After OFC, doses were increased until a daily dose of 1800 mg peanut protein was reached, provided that peanut-specific IgE was > 2 kU_A_/L after 1 year on maintenance dose. Evaluation of subjects every 4 months during maintenance phase (up to total duration of 36 months). OFC up to 3.9 g peanut protein or until objective symptoms appeared.	27 of 29 (93%) reached total peanut dose of 3.9 g in OFC after 36 months without showing more than mild symptoms and were thus considered desensitized. The other 2 stopped OFC after 2.1 g peanut protein. 7 subjects underwent open OFC to peanut protein after 13-22 months of maintenance dosing; 22 underwent OFC after 4-7 months	Within 4 months, basophil reactivity at peanut concentration of 10 µg/ml was significantly reduced. Within 3 months, peanut-specific IgE levels increased from an initial median concentration of 85.4 kU_A_/L to 249.0 kU_A_/L. For all time points after 18 months (up to 33 months) peanut-specific IgE levels were decreased. An increase of specific IgG levels was observed starting at 3 months of treatment and remained high until 24 months, before it returned to baseline by 33 months. Peanut-specific IgG4 levels reached significance at 3 months and increased until the end of the study. Several inflammatory cytokines/chemokines (IL-1β, IL-5, TNF-α, MIP-1β, G-CSF and GM-CSF) were increased over time (following peanut stimulation). At 6 and 12 months FoxP3 T cells increased 1.5-fold in peanut-stimulated cells before returning to baseline by 20 months.	Symptoms were reported after 46% of build-up doses. During maintenance phase, all subjects experienced adverse events at some point, which were mostly mild and affected most commonly the upper respiratory tract and the skin. Two of the participants received epinephrine once during home dosing.	NCT01074840	(249)
**Peanut:** whole crushed roasted peanuts; 4 g whole peanut = 1 g peanut protein**;** dose of individual major allergens not determined	23 initial subjects (3-14 years); 14 finished study protocol (until final DBPCFC)	Open-label, randomized	Participants underwent DBPCFC with increasing doses of whole peanut (0.03-2 g), equaling 0.0075-0.5 g peanut protein, which were given every 30 minutes (on 2 different days). In absence of objective reaction, patients were challenged on another day with 4 g whole peanut. The day after positive DBPCFC followed a rushed escalation protocol for 1 week during which increasing doses of whole peanuts were given 2-4x a day. The staring dose was approx. 1/100 of the reaction eliciting dose during DBPCFC. In those starting with more than 6 mg whole peanuts, doses were doubled. If starting point was 80 mg, doses were increased by 20%. Subjects that reached at least 500 mg whole peanut during the rushed protocol, continued with a maintenance phase of 8 weeks. Subjects that did not reach a dose of 500 mg peanut continued with individual long-term build-up protocol (0-20 months) during which the individual tolerated dose (24-400 mg peanut) was consumed daily, with dose increases every 2-4 weeks until 500 mg was reached, followed by a maintenance phase of 8 weeks. After 2 weeks of peanut avoidance, final DBPCFC was performed.	5 patients reached 500 mg peanut dose during the rushed protocol. Overall, 14 of 22 patients reached a daily maintenance dose of at least 500 mg whole peanut after a median of 7 months and underwent DBPCFC. At final DBPCFC, a median of 1 g peanut was tolerated. Three patients tolerated 4 g whole peanut. Median tolerated dose before OIT was 0.19 g peanut.	In the 14 patients that finished the study protocol, a reduction in the secretion of IL-5, IL-4, and IL-2 at the end of the OIT treatment (before avoidance) was observed and seen to be stable in most, but not all of the patients after avoidance phase. An increase in peanut-specific IgG4 levels was seen in all patients after OIT. However, a drop in the peanut-specific IgG4 level was detected after 2 weeks of avoidance. Patients that reached 500 mg peanut had lower median peanut-specific IgE levels (9.1 kU_A_/L) that those that tolerated less (212 kU_A_/L).	Of 6137 total OIT doses, 2.6% were associated with mild to moderate adverse effects. 4 patients stopped OIT due to adverse reactions. All of them had reported mild to moderate asthma before start of the study. During the rush protocol, objective allergic symptoms were associated with 25 of 317 total OIT doses.	No clinical trial number found; study was approved by the local ethics committee	(250)
**Peanut:** Peanut protein extracted from defatted peanut four (50% protein); intact allergen content in soluble extract of roasted peanut flour ∼8% Ara h 1 and ∼7% Ara h 2	Initially 28 (1-16 years) participants; 3 withdrew; 16 remained in peanut OIT group, 9 in placebo group	Randomized, placebo-controlled	Initial day escalation phase starting with 0.1 mg peanut protein (or placebo). Doses were doubled every 30 minutes up to 6 mg. Build-up phase started with highest tolerated dose in initial escalation. During home-dosing, subjects ingested daily doses and attended build-up visits every two weeks for approximately 44 weeks. Doses were increased by 50-100% until 75 mg and 25-33% until daily maintenance dose of 4000 mg was reached. The maintenance dose was consumed daily for one month, followed by an OFC at week 48.	16 of originally 19 participants (84%) in the OIT group reached a maintenance dose of 4000 mg and tolerated a maximum cumulative dose of 5000 mg peanut protein in OFC compared to a median cumulative dose of 280 mg peanut protein in the placebo group.	In the peanut OIT group, median peanut-specific IgE increased from baseline level of 104 kU_A_/L to 308 kU_A_/L by two months, but was not significantly different to baseline at time of challenge. No difference in the IgE levels were observed in the placebo group. At all time points, peanut OIT subjects showed increase in peanut-specific IgG levels, including IgG4, which were not increased in the placebo group. IL-5 and IL-13 levels significantly decreased in the peanut OIT group from baseline to 9 months and OFC, while there was no change in the placebo group. In the peanut OIT group, an increase in the ratio of FoxP3^hi^: FoxP3^intermediate^ CD4+CD25+ Treg cells at time of challenge was observed compared to the baseline. This did not apply for subjects in the placebo group.	During initial dose-escalation, 9 of 19 subjects in the peanut OIT group had clinically-relevant adverse effects and required antihistamine treatment. Of those, 2 additionally required treatment with epinephrine. No clinically-relevant symptoms were reported in the placebo group. Of 407 build-up doses, 1.2% caused clinically-relevant symptoms in the peanut OIT group. During home dosing, none of the peanut OIT subjects required epinephrine. In the placebo group, one subject received epinephrine after reporting symptoms. One patient in the peanut OIT group experienced mild-moderate symptoms after completing OFC and was given antihistamine treatment. In the placebo group, 8 subjects experienced side effects during OFC, 3 required epinephrine treatment.	No clinical trial number found; study approved by each institution’s Institutional Review Board	(251)
**Peanut:** peanut flour (50% protein); dose of individual major allergens not determined	22 subjects (4-18 years)	Interventional, open-label	Gradual build-up phase (56-264 days) with dose increases every 2 weeks up to 800 mg peanut protein per day. After reaching the highest tolerated dose, subjects continued with maintenance for 30 weeks during which dose was ingested on a daily basis. Patients underwent DBPCFCs after 6 weeks of maintenance and at the end of the study (week 30).	Primary endpoint was defined by rate of those passing challenge after approx. 6 months. Of 22 subjects, 19 tolerated build-up to a maximum daily dose of 800 mg peanut protein and successfully continued maintenance. After 6 weeks of maintenance, 19 subjects underwent OFC to 2.6 g peanut protein, 18 ingested the full dose. 12 of 19 (63%) had no symptoms during challenge, 7 (37%) showed mild to moderate symptoms. After 30 weeks, 18 subjects underwent final challenge with 6.6 g peanut protein. 14 of 18 subjects tolerated challenge without any symptoms.	Median peanut-specific IgE levels increased initially, before decreasing until week 30 (8.35 kU_A_/L) compared to the baseline (29.7 kU_A_/L). Median peanut-specific IgE level was significantly lower at baseline in the participants that passed final OFC compared to those that did not.	At some point during build-up and maintenance phase, 19 of 22 (86%) subjects experienced adverse reactions, most commonly affecting the respiratory or gastrointestinal tract.	NCT01259804	(252)
**Peanut:** peanut flour (50% protein); dose of individual major allergens not determined	99 participants (7-16 years) were randomized: 49 in peanut OIT group (10 did not have DBPCFC after OIT), 50 in the peanut avoiding control group (46 included in primary analysis)	Randomized, controlled (crossover)	Initial gradual up-dosing phase with biweekly increases until a target protein dose of 800 mg/day was reached. This was followed by a maintenance period with ingestion of the highest tolerated dose on a daily basis to complete 26 weeks of OIT. During the 26-week long first phase, subjects received peanut OIT or avoided peanut (control group). During the second phase (crossover), subjects in the control group received peanut OIT, followed by DBPCFC. Toleration of a cumulative dose of 1400 mg peanut protein during DBPCFC was considered desensitization.	Primary endpoint was defined as desensitization. In the first phase, 24 of 39 (62%) participants in the active OIT group compared to 0 of 46 (0%) in the control group tolerated a cumulative dose of 1400 mg peanut protein in OFC. 84% in the active group tolerated a daily dose of 800 mg peanut protein (secondary outcome). In the second phase (control group after OIT), 91% tolerated daily dose of 800 mg protein and 54% tolerated 1400 mg in OFC.	Increase in peanut-specific IgE was measured after 24 weeks in the OIT group.	Adverse reactions were reported to the same extend in both groups during treatment but were mostly mild. Oral itching occurred in 6.3% of all doses. 0.41% of doses in 22% of subjects caused wheezing which was treated either with antihistamines alone or, in one patient, additionally with epinephrine on two occasions. Cutaneous symptoms were reported after 0.16% of doses.	ISRCTN62416244	(253)
**Peanut:** peanut protein from partially defatted peanut flour (50% protein); see (249)	Initially 39 subjects (1-16 years) included; 24 completed the protocol	Open-label	End-of-study results of pilot trial by (249). OIT protocol by (249), which was described above. Extended treatment with a maximum of 4000 mg peanut protein per day for up to 5 years. At the end of the treatment, subjects underwent two DBPCFCs to 5 g peanut protein, 4 weeks apart. During these 4 weeks OIT was not continued in order to evaluate sustained unresponsiveness.	12 of 24 (50%) subjects showed treatment success by reaching 5000 mg peanut protein in the second OFC, 4 weeks after stopping OIT, and achieved sustained unresponsiveness (primary endpoint). In the first OFC, which was performed after a maximum of 5 years of OIT with 4000 mg peanut protein per day, all the subjects successfully ingested 5 g of peanut protein.	Patients that passed final OFC had lower median IgE levels specific for peanut allergens Ara h 1 and Ara h 2, than those that did not achieve sustained unresponsiveness. In all OIT subjects, a reduction to below baseline IgE levels specific for major peanut allergens (Ara h 1, 2, 3) was observed. Peanut-specific IgG levels, including IgG4, increased in all participants. However, IgG4 production was not associated with the clinical outcome of the study. Ara h 2-specific IgE levels were the best predictor of sustained unresponsiveness, followed by peanut-specific IgE levels.	6 of the initial 39 subjects withdrew due to allergic side effects (not further specified).	no clinical trial number found; ethics approval obtained through the Institutional Review Boards at Duke University Medical Center and University of Arkansas for Medical Sciences	(254)
**Peanut:** peanut powder used for OIT (protein content not given); dose of individual major allergens not determined; oat flour used for placebo OIT	21 subjects (7-13 years); 10 in active SLIT/placebo OIT group and 11 in active OIT/placebo SLIT group; 16 completed protocol (7 in active OIT group)	Randomized, double-blinded, placebo-controlled	Initial dose escalation starting with 0.1 mg peanut protein up to 6 mg. Dose increases every 1-2 weeks until a maintenance dose of 2000 mg/day reached. Doses were ingested on a daily basis for 16 weeks. The maintenance dose was taken daily for 12 months. OFC with 10 g peanut powder was performed at 6 and 12 months of maintenance. In those, that completed OFC without more than mild symptoms discontinued treatment for 4 weeks and were then rechallenged, all of the others proceeded with unblinding phase for additional 6 months. Subjects that reacted during OFC at 12 months to less than 5 g continued treatment with SLIT added. Subsequently, subjects underwent OFC with 10 g peanut protein. Those that tolerated the challenge discontinued treatment for 4 weeks and were then rechallenged.	Primary endpoint, defined as a toleration of at least 10-fold increase in OFC threshold after 12 months of treatment, was achieved by 7 of originally 11 subjects in active OIT group (considered desensitized). In the original active OIT group, 1 subject passed OFC at 12 months to 10 g peanut protein and was rechallenged after 4 weeks of treatment discontinuation. 3 extended the prior treatment for 6 months and another 3 continued OIT with SLIT added, before being rechallenged. 3 of originally 11 subjects in the active OIT group achieved sustained unresponsiveness.	In SLIT and OIT group peanut-specific IgE levels increases initially but decreased over the time of the treatment. In the OIT group median peanut-specific IgE was 68 and 53 kU_A_/L after 6 and 12 months compared to 169 kU_A_/L at the baseline. Peanut-specific IgG4 increased in both groups during treatment. In the OIT group IgG4 medial levels increased from 1.3 mgA/L at baseline to 76 mgA/L after 12 months.	2 subjects in the active OIT group discontinued treatment due to adverse reactions (one with gastrointestinal symptoms, one with systemic reaction). 43% of OIT doses were associated with adverse reactions. All subjects in the OIT group had symptoms with dosing. Epinephrine was required by one subject in the active OIT/additional active SLIT group during maintenance. 4 subjects in the OIT group required 5 doses of epinephrine. Overall, adverse reactions were more common in the OIT group.	NCT01084174	(255)
**Peanut:** peanut flour (50% protein); dose of individual major allergens not determined	11 subjects (4-16 years)	Open-label	Entry dose chosen based on threshold dose of reactivity. Dosing was increased approx. every 2 weeks during build-up phase until a maintenance dose of 2000 mg peanut protein was reached. The median time to maintenance was 41 weeks. After approximately 4 months of maintenance, 5000 mg DBPCFC was performed. Participants received 2000 mg peanut protein maintenance dose per day after DBPCFC.	9 of 11 subjects achieved maintenance dosing of 2000 mg peanut protein per day and passed 5000 mg DBPCFC, with 6 of 9 (66%) not showing symptoms during challenge.	Significant changes of peanut-specific IgE, IgG4 and IgE/IgG4 6 weeks after therapy.	264 of 3265 doses (7.9%) were associated with reported side effects, which were mostly mild. In 2 cases severe reactions were reported.	No clinical trial number found; study was approved by the University of Texas Southwestern Institutional Review Board	(256)
**Peanut:** peanut flour (50% protein) together with L*actobacillus rhamnosus* CGMCC 1.3724; dose of individual major allergens not determined; placebo group received maltodextrin	Initially 62 subjects (1-10 years); 6 withdrew from study; 56 reached end of trial: 28 in OIT group, 28 in placebo control group	Randomized, double-blind, placebo-controlled	Peanut OIT in combination with probiotic (PPOIT) was given. Initial 1-day rush dose escalation phase starting with 0.1 mg peanut protein up to a final dose of 12 mg. Build-up phase (approx. 8 months) with biweekly dose increases until daily tolerated dose of 2000 mg peanut protein reached, followed by maintenance for 12 months. If maintenance dose reached in more than 12 months, extension of total duration to ensure 6 months of maintenance. DBPCFC performed at last day of treatment (confirmation of desensitization) and repeated challenge 2-5 weeks after stopping treatment (confirmation of sustained unresponsiveness) in those that passed the challenge.	Possible sustained unresponsiveness was achieved in 23 of 28 (82.1%) PPOIT subjects and 1 of 28 (3.6%) in the placebo group (primary endpoint). Desensitization was achieved in 26 of 29 (89.7%) PPOIT-treated and 2 of 28 (7.1%) placebo-treated subjects.	After treatment, an overall reduction in peanut-specific IgE levels compared to the baseline (median, −4.45 kU_A_/L) was seen in the PPOIT-treated group together with an increase in peanut-specific IgG4 (median, 3.24 mgA/L). This did not apply for the placebo group.	At least 1 severe adverse reaction was reported in 45.2% in the PPOIT group and 32.3% subjects in the placebo group. Total number of severe events was greater in PPOIT group than placebo group. Overall, 6 treatment-related severe adverse events occurred in 3 patients in the PPOIT group, and 4 adverse reactions occurred in 4 placebo-treated patients.	ACTRN12608000594325	(257)
**Peanut:** peanut margarine made from roasted defatted peanut flour (50% protein); dose of individual major allergens not determined	60 subjects (6-18 years): 39 active OIT, 21 controls that avoided peanuts	Interventional	Patients ingested daily doses of peanut protein starting with 0.1 mg and dose escalations every 1-2 weeks. Build-up phase (approx. 8 months) until maintenance daily dose of 800 mg peanut protein (4 peanuts) was reached. DBPCFC was performed 1 month after reaching maintenance dose. Afterwards, subjects ingested 3-7 weekly doses of 4 raw or roasted peanuts. Patients that failed challenge continued with tolerated daily dose. Median follow-up period was 30 months.	33 of 39 (85%) OIT-treated patients reached daily maintenance dose (800 mg peanut protein) in a median of 269 days. 26 patients (67% in intention-to-treat analysis) passed challenge with 1255 mg peanut protein (5 g peanuts) (primary endpoint). None of the 21 controls showed desensitization. Median follow-up duration was 30 months. During the follow-up phase the median weekly peanut protein consumption was 5600 mg.	OIT had no significant effect on peanut-specific IgE to Ara h 1, 2, 3, 8, or 9. Specific IgG4 levels to peanut, Ara h 1, 2 and 3 increased significantly during the treatment. No difference was observed in the avoidance group. In 29 subjects that continued OIT (1-year follow-up) peanut-specific IgE levels to major peanut allergens (Ara h 1, 2, 3) decreased significantly.	30 of 39 (77%) OIT subjects reported adverse symptoms during build-up. 16 of 39 (41%) needed additional antihistamines, 15 of 39 (38%) received prednisolone and 1 of 39 (2.6%) used epinephrine autoinjector.	NCT01502878	(258)
**Peanut:** peanut flour (50% protein), for low dose mixed with oat flour; dose of individual major allergens not determined	37 subjects (9-36 months) eligible for study (5 withdrew); 154 standard-care controls	Randomized, double-blind, controlled	Initial dose escalation. Buildup-phase for 42 weeks until maintenance dose reached. Patients received either low- or high-dose early OIT (maintenance dose 300 mg or 3000 mg peanut protein/day) and underwent 2 final DBPCFCs after a maintenance phase of up to 36 months. Unresponsiveness 4 weeks after stopping OIT (4-SU) was defined by toleration of 5 g peanut protein (cumulative) during DBPCFC.	subjects underwent first DBPCFC to 5 g peanut, which two failed. The others repeated challenge after 4 weeks of peanut avoidance, which was completed by 29 patients. Thus, 29 of 37 (78%) achieved 4-SU (primary endpoint): 17/20 (85%) in low-dose, 12/17 (71%) in high-dose group. 4-SU was achieved over a median of 29 months. 30 of 37 (81%) subjects achieved desensitization by the end of the treatment (intention-to-treat analysis): 17/20 (85%) in low-dose and 13/17 (76%) in high-dose group	Over the time of the study, median peanut-specific IgE level declined in OIT treated subjects (1.6 kU_A_/L), compared to the baseline (14.4 kU_A_/L), while there was an increase in control subjects (57.4 kU_A_/L compared to 21.9 kU_A_/L at baseline). Treatment success correlated with lower peanut-specific IgE and peanut-specific IgE/total IgE ratio at baseline.	Of the initial 37 eligible study participants, 3 withdrew due to treatment-related adverse reactions. Overall, 95% of the participants were affected by adverse events which occurred more frequently during the build-up phase. Most adverse events were mild (85%), 15% were considered moderate and no severe reaction was reported.	NCT00932828	(259)
**Peanut:** AR101 peanut powder capsules containing 0.5-100 mg peanut protein; relative potency of Ara h 1, 2, and 6 determined to ensure content uniformity together with determination of additional allergen molecules such as Ara h 3 and Ara h 8; oat flour containing capsules for placebo group	55 subjects (4-26 years): 29 AR101 treated, 26 in placebo group	Randomized, double-blind, placebo-controlled	During the initial dose escalation day, doses were increased from 0.5 mg to a maximum of 6 mg. OIT subjects received daily AR101 or placebo with dose increases every 2 weeks to a final daily dose of 300 mg (20-34 weeks). Patients that tolerated daily dose of 300 mg for 2 consecutive weeks were eligible for final DBPCFC.	The primary endpoint, defined as the rate of subjects that completed final DBPCFC to a cumulative dose of at least 443 mg peanut protein (cumulative), was achieved by 23 of 29 (79%) in the AR101 group (Intention-to-treat population) and 5 of 26 (19%) in the placebo group. 18 of 29 (62%) in the AR101 group tolerated 1043 mg (cumulative) during DBPCFC compared to 0% in the placebo group.	In the AR101 group a significant increase in peanut-specific IgG4 levels was observed while almost no difference was seen in the placebo group. No statistically significant difference in peanut-specific IgE levels was seen between both groups during the treatment.	28/29 subjects (96.6%) in the AR101 group and 22/26 (84.6%) in the placebo group experienced at least 1 adverse event. Of the 23 AR101 subjects that passed 443 mg challenge, 3 (13%) had mild symptoms. In the placebo group, 10 subjects (38%) experienced severe symptoms during DBPCFC, 2 occurred at cumulative dose of 43 mg. At cumulative dose of 1043 mg 61% of AR101 subjects and none of the placebo subjects were symptom free. During final DBPCFC 11/26 (42%) placebo subjects and 2/23 (9%) AR1010 subjects received epinephrine.	NCT01987817	(260)
**Peanut:** AR101 peanut powder in capsules (doses of 0.5-100 mg) or foil-laminate sachets (300 mg); quantities administered reported as mg of peanut protein; for further information see (260)	496 subjects (4-17 years), 372 in active treatment group, 124 in placebo group	Randomized, double-blind, placebo-controlled	Initial dose-escalation day with doses from 0.5 to 6 mg. Doses were ingested on a daily basis and were increased every 2 weeks starting at 3 mg, until 300 mg peanut protein were tolerated. Maintenance dose was ingested for 24 weeks. At the end of the study (approx. 12 months) subjects underwent final DBPCFC.	Primary endpoint was proportion of subjects that responded to treatment and were able to ingest a single dose of at least 600 mg peanut protein during final DBPCFC without dose-limiting effects. This was achieved by 250 of 372 (67.2%) participants in the active treatment group, compared to 5 of 124 (4.0%) in the placebo group. 76.6% and 50.3% in the active OIT group tolerated 300 mg and 1000 mg peanut protein dose during DBPCFC. In comparison, 8.1% and 2.4% in the placebo group tolerated 300 mg and 1000 mg dose, respectively.	Peanut-specific IgG4 levels increased during treatment in the active OIT group. There was no significant between-group difference in regard to peanut-specific IgE levels from baseline to the trial endpoint.	During final DBPCFC, 25% in the OIT group and 59% in the placebo group experienced moderate symptoms. Severe symptoms were reported in 5% in the active group compared to 11% in the placebo group. In the active OIT group, 10% of the participants received epinephrine during final food challenge compared to 53% in the placebo group. During the intervention period (excluding final OFC), 98.7% in the OIT group and 95.2% in the placebo group had an adverse event. In the active OIT group, 34.7% and 59.7% experience mild and moderate reactions, respectively. In the placebo group 50% had mild and 44.4% had moderate side effects. Severe adverse events were reported in 4.3% in the OIT group and 0.8% in the placebo group.	NCT02635776	(261)
**Peanut:** peanut powder (protein content not specified); dose of individual major allergens not determined	24 (5-18 years) subjects with history of anaphylaxis in OIT group, 10 historical controls (avoided peanuts)	Open-label	Subjects ingested peanut powder 2x/day during 5 days of hospitalization. Up to 1 month after discharge, dosing was continued with amount decided at time of discharge. If dose was tolerated for 5 consecutive days after this month, dose was increased gradually until a target dose of 133 mg peanut protein/day was reached. Patients visited hospital every 1-3 months (total duration 12 months). One year after staring the treatment, patients stopped intake for 2 weeks and then underwent 133 mg and 795 mg OFC (on two consecutive days). Patients that passed the challenges continued with weekly ingestion of 795 mg peanut protein. Those without showing symptoms 3 months after OFC were considered having achieved sustained unresponsiveness.	After 12 months of treatment, 8 (33%) children in the OIT group achieved sustained unresponsiveness compared to 0% in the control group (primary endpoint). 22 of 24 (92%) participants in the OIT group achieved desensitization within 12 months. After 1 year, 16 (67%) of the OIT-treated children tolerated 133 mg and 14 (58%) tolerated 795 mg in OFC compared to 1 of 10 (10%) and 0 of 10 (0%) in the historical control group.	The median peanut- and Ara h 2-specific IgE levels increased significantly during the first month, and then decreased at 3, 6 and 12 months. Median peanut- and Ara h 2-specific IgG and IgG4 levels increased significantly from baseline to 1 month in the OIT group, while no changes were observed in the control group. Baseline Ara h 2-specific IgE levels were predictive for the achievement of sustained unresponsiveness.	In total, 79 of 119 admission doses (66.4%) caused allergic reactions, but none of them were severe. During home dosing, 9.1% of the subjects experienced adverse symptoms, which were severe in 0.01% of cases. One child required treatment with epinephrine.	UMIN000011202	(262)
**Peanut:** peanut paste made from roasted peanut (protein content approximately 20%); dose of individual major allergens not determined	Initially 30 subjects (12-18 years): 21 in peanut OIT group, 9 placebo controls; 2 patients withdrew	Randomized, double-blind, placebo-controlled	Initial DBPCFC, followed by 24 weeks of build-up phase during which subjects ingested daily doses between 2-400 mg peanut protein. Doses were increased every 2 weeks until subjects reached daily doses of up to 400 mg peanut protein. At the end of the build-up phase, subjects underwent DBPCFC.	Primary endpoint was defined by toleration of at least 400 mg (cumulative) peanut protein during DBPCFC, performed 1-3 days after the end of the build-up phase. This was achieved by 17 of 21 (81%) OIT treated subjects compared to 1 of 9 (11%) in the placebo control group (intention-to-treat analysis). 17 of 19 patients in the OIT group that finished the build-up protocol increased their reactivity threshold 4-fold between first and second DBPCFC compared to 2 of 9 in the placebo group.	Peanut-specific IgE levels increased significantly in the OIT group compared to the placebo group at second DBPCFC. No significant difference was observed for Ara h 1-, 2- and 3-specific IgE levels between first and second DBPCFC. Peanut-specific IgG4 levels increased significantly during build-up phase. The same applied for IgG4 levels specific for major peanut allergens. Peanut and Ara h 2-specific IgG4/specific IgE ratios increased in the OIT group at the second DBPCFC, and reached significance for Ara h 2. No difference in the peanut-specific IgE/total IgE ratio was observed in either group during the build-up phase.	Two patients in the OIT group withdrew during the build-up phase. One due to a severe reaction that required epinephrine and the other due to moderate side effects. Overall, only 3 subjects experienced no adverse event during the build-up phase. While there was no difference between the number of patients with adverse events between both groups, the number of events/patients was higher in the OIT group. In 91/1000 doses medication was required in the OIT group, compared to 36/1000 in the placebo group. Five systemic reactions occurred in 4 OIT-treated patients; one was life-threatening.	NCT02046083	(263)
**Peanut:** peanut flour (50% protein) in vehicle of chocolate pudding; dose of individual major allergens not determined; placebo group only received vehicle without peanut flour	62 subjects (3-17 years): 31 in OIT group, 31 placebo controls	Randomized, double-blind, placebo-controlled	Initial dose escalation phase during which patients received whole crushed roasted peanuts starting with 3 mg peanut protein in 2-hour intervals for a maximum of 3 days until 4500 mg peanut protein was reached or objective symptoms were observed. OIT was started with doses of 0.5-30 mg peanut protein, depending on eliciting dose during initial OFC. Doses were taken on a daily basis and increased approximately every 2 weeks (up to 14 months). Patients with eliciting dose of 3-100 mg during initial OFC had goal maintenance dose of 125 mg, subjects with an eliciting dose of 300-4500 mg should reach a maintenance dose of 250 mg peanut protein. Maintenance dose was continued for 2 months (+/- 2 weeks).	Primary endpoint was the proportion of subjects tolerating a single dose of at least 300 mg peanut protein during final OFC, which was achieved by 23/31 (74.2%) in the active OIT group versus 5/31 (16.1%) in the placebo control group. 13 of 31 (41.9%) subjects in the active OIT group compared to 1 of 31 (3.2%) in the placebo group tolerated 4.5 g peanut protein at final challenge. 50% in each group reached the goal maintenance dose (peanut protein or placebo).	In the peanut-OIT group a significant reduction in IL-4, IL-5, IL-10 and IL-2 production, a significant increase in median peanut-specific IgG4 levels and a decrease in the peanut-specific IgE/IgG4 ratio were observed after treatment when compared between the randomized arms.	Two patients in each group withdrew due to experiencing adverse events, which were severe in one of the subjects of each group. All patients experienced adverse events at some point, however, only 1.2% of placebo doses and 4.3% of peanut OIT doses were associated with treatment-related adverse reactions. 40% in the placebo group and 45% in the active OIT group needed at least 1 dose reduction because of reported side effects.	DRKS00004553	(264)
**Peanut:** suspension of peanut flour in Kool-Aid containing 2.5 µg of peanut protein; with increasing doses alternative forms of peanut were provided with equivalent doses of peanut protein; dose of individual major allergens not determined	270 subjects (4-18 years)	Retrospective record	Retrospective medical record review of OIT treated patients between 2009 and 2017. Initial dose escalation phase with ingestion of tolerated dose 2x/day for at least 1 week before participants returned for another dose increase. Buildup phase until target dose of 3000 mg peanut protein reached (individual duration). After 6000 mg peanut protein challenge, maintenance dose of 2000 mg was taken once or twice daily for at least 3 years. Sustained unresponsiveness was defined by passing 6 g DBPCFC 30 days after stopping OIT.	214 of 270 (79%) subjects reached target maintenance dose (211 reached target dose of 3000 mg peanut protein, 3 reduced target of 2000 mg) and were challenged with 6000 mg peanut protein, with all except one passing the challenge 14 of 214 (6.5%) patients achieved sustained unresponsiveness.	A decrease in peanut-specific IgE levels was observed during the maintenance period. In 54 tested patients, peanut-specific IgG4 level after reaching maintenance was > 80 µg/mL, however, measurement was discontinued afterwards.	During dose escalation, 63 of 270 (23%) patients required treatment with epinephrine. In total, 157 subjects (58%) reported 330 minor adverse reactions that did not require treatment with epinephrine.	No clinical trial number found; study was approved by the North Texas Institutional Review Board	(265)
**Walnut:** doses given as mg of walnut protein (not further specified); dose of individual major allergens not determined	73 subjects (4 years or older), 55 in OIT group, 18 observational controls (dietary exclusion)	Prospective	Initial dose escalation over 4 days (in in an ambulatory care setting). The highest tolerated dose was consumed daily for 24 days. Each month patients returned for dose escalations followed by daily dose intake until target dose of 4000 mg walnut protein was reached (was considered desensitization). Maintenance of 1200 mg walnut protein/day for 6 months in those patients that were desensitized, followed by OFC to 4000 mg walnut protein. Crossover of control group after observation period (median period 7.1 months).	Primary study endpoint was defined as toleration of 4000 mg walnut protein (26 g walnut) by the end of the study (desensitization). Desensitization was achieved in 49 of 55 (89%) patients (intention-to-treat analysis) in the OIT group, compared to 0 of 18 (0%) in the control group. Patients that were co-allergic to pecan (n = 46) also showed desensitization to pecan. 18 of 30 (60%) with co-allergy to hazelnut or cashew and 14 of 15 (93%) with co-allergy to hazelnut alone were considered either fully desensitized or treatment responders.	In the walnut OIT group, walnut-specific IgE levels, CD63 expression in BAT and IgE/IgG4 ratio significantly decreased, while walnut-specific IgG4 levels increased during the treatment period (similar results for antibody levels to all walnut-specific components). This did not apply for the control group.	In total, 47 (85%) of 55 patients in OIT group experienced adverse reactions during in-hospital up-dosing and 40 (73%) during home-dosing. However, reactions were mostly mild and occurred in response to 109 (4%) of in-clinic doses and 244 (2%) of home doses. Epinephrine treatment was required by 11 patients during in-hospital phase and 8 during at home treatment.	No clinical trial number found; study was approved by the institutional review board	(266)
**Peanut:** peanut flour**;** protein content was calculated and confirmed through protein assays (not further specified); oat flour for placebo group used	120 subjects (7-55 years): 60 in peanut-0 group, 35 in peanut-300 group, 25 in placebo group	Randomized, double-blind, placebo-controlled	Buildup phase until maintenance dose of 4000 mg peanut protein reached (week 104), followed by discontinuation (peanut-0 group), daily intake of 300 mg peanut protein (peanut-300 group) or placebo for 52 weeks. DBPCFC every 3 months if a cumulative dose of 4000 mg peanut protein was tolerated during previous challenge.	Primary endpoint was defined of proportion of subjects that tolerated a cumulative dose of 4000 mg peanut protein during DBPCFC at week 104 and week 117. At week 104, 51 of 60 (85%) peanut-0 subjects, 29 of 35 (83%) peanut-300 subjects and 1 of 25 (4%) in the placebo group passed DBPCFC. At week 117, 21 of 60 (35%) peanut-0 subjects, 1 of 25 (4%) in the placebo group and 19 of 35 (54%) peanut-300 subjects passed 4000 mg challenge. In the peanut-0 group, 8 of 60 (13%) participants passed 4000 mg challenge after week 156 compared to 13 of 35 (37%) in the peanut-300 group and 1 of 25 (4%) in the placebo group.	Lower peanut- and Ara h 2-specific IgE levels were associated with passing challenge at week 117 in the peanut-0 and the peanut-300 arms. In the peanut-0 group, higher peanut-specific IgG4/peanut-specific IgE ration was associated with week 117 success, however, this did not apply for the peanut-300 group. A higher Ara h 2-specific IgE/peanut-specific IgE ratio was associated with higher risk of treatment failure.	Two patients withdrew due to severe adverse events. During the first year, 95% in the peanut-0 group, 91% in the peanut-300 group and 64% in the placebo group reported adverse events. In the third year, adverse events were reported by 2% in the peanut-0 group, 20% in the peanut-300 group and 5% in the placebo group.	NCT02103270	(267)
**Hazelnut:** doses given as mg of hazelnut protein; 259 mg hazelnut protein equivalent to 1 whole hazelnut; dose of individual major allergens not determined	100 subjects (3-9 years)	Retrospective	DBPCFCs were performed at time of diagnosis and 6 months after starting OIT. During challenge doses were increased every 20 minutes up to a cumulative dose of 1635 mg hazelnut protein. Buildup phase started with one-tenth of eliciting dose from initial DBPCFC. Monthly dose increases until tolerated cumulative dose of 1635 mg hazelnut protein (equivalent to 8 whole hazelnuts) during OFC (performed after 6 months) was reached. After passing OFC, subjects continued with maintenance dose of 416 mg hazelnut protein 3x/week. If OFC was failed, schema was repeated until desensitization was achieved.	Primary endpoint was defined by proportion of desensitized subjects after 6 months of OIT treatment. 34 of 100 (34%) patients tolerated 1635 mg hazelnut protein during OFC after 6 months and were considered desensitized Patients without desensitization repeated procedure based on eliciting dose from 6-month OFC.	Desensitization to hazelnut was associated with lower hazelnut specific- and Cor a 14-specific IgE levels.	76 patients completed a survey about OIT side effects and 30% reported at least one side effect (non-severe). No serious adverse reactions were reported.	NCT03048149	(268)
**Peanut:** AR101 = drug consisting of peanut flour; see (Bird et al., 2018)	175 subjects (4-17 years): 132 in AR101 group, 43 in placebo group; 106 of the AR101 group and 40 of the placebo group completed the study	Randomized, double-blind, placebo-controlled (phase 3)	Build-up phase with biweekly dose increases (20-40 weeks) until daily dose of 300 mg peanut protein (AR101) reached, followed by maintenance for 3 months.	Primary endpoint was defined by proportion of subjects that could consume 1000 mg (cumulative dose 2043 mg) peanut protein at final DBPCFC (after 9 months) without dose-limiting effects. 77 of originally 132 (58%) subjects in the AR101 group passed challenge compared to 1 of 43 (2%) in the placebo group.	In the AR101 group peanut-specific IgG4 levels increased during the study. No significant difference in the change of peanut-specific IgE levels was observed between the active treatment and the placebo group. A reduction in the IgE/IgG4 ratio by the end of the trial in comparison to the initial screening in the AR101 group.	Adverse reactions were reported by almost all subjects, but were mostly mild to moderate in both, AR101 and placebo group. One severe adverse event was reported in the AR101 group. Gastrointestinal disorders were reported by 91% in the active treatment group and 77% in the placebo group.	NCT03201003	(269)
**Peanut:** PTAH, formerly AR101 = drug consisting of peanut flour; see (261)	358 eligible subjects (4-17 years): 256 in original active treatment group, 102 in original placebo group	Open-label, follow-on study to (261)	Patients that reached 300-mg dose at the exit DBPCFC in previous study and placebo group entered the follow-on study. Subjects were assigned to 5 dosing cohorts, receiving either daily doses of 300 mg (cohorts 1 and 3A) or non-daily doses (cohorts 2, 3B, 3C). PTAH-naïve subjects (from initial placebo group) underwent buildup to daily dose of 300 mg, followed by maintenance. At the end of the study (approx. 2 years), subjects underwent DBPCFC up to 2000 mg peanut protein (highest dose).	Cohort 3A (300 mg daily for approx. 56 weeks) had highest desensitization rates In PTAH-native group, desensitization rates at challenge doses of 2000 mg were 45.8% at maintenance challenge (after 24 weeks of 300 mg daily maintenance) and 51.4% at exit challenge (52 weeks after maintenance challenge).	IgG4 levels increased during the study period in daily-dosing cohorts. IgE levels decreased from PALISADE entry to study exit in PTAH-naïve and PTAH-continuing subjects.	83% of subjects experienced mild to moderate adverse events. 6.1% of subjects in non-daily dosing group had to revert to daily dosing due to adverse events. Severe adverse events occurred in 2.1% of daily-treated and 2.7% of non-daily treated subjects. 7 participants in PTAH-continuing group withdrew due to adverse events. In the PTAH-naïve group, 86% experienced treatment-related adverse events. 5 subjects (from cohorts 1 and 3C) experienced treatment-related anaphylaxis.	NCT02993107	(270)
**SLIT**								
**Hazelnut:** hazelnut extract in glycerosaline solution; dose of individual major allergens not determined; saline solution used as placebo	22 subjects (18-60 years): 11 in active treatment group, 11 in placebo group	Randomized, double-blind, placebo-controlled	Patients kept allergen solution in the mouth for at least 3 minutes before spitting out (sublingual-discharge technique). SLIT was performed with hazelnut extract of 5 strengths (F0, F1, F2, F3, FA). 4-day build-up phase during which doses were given in 15-minute intervals, followed by daily maintenance dose of 5 drops of maximum concentration (vial concentration (FA) 66.25 mg/ml) for 8-12 weeks. Treatment efficacy was assessed by DBPCFC at the end of the study.	5 of 11 (45%) in the SLIT group and 1 of 11 (9%) in the placebo group tolerated the highest level of 20 g raw hazelnuts (15-20 hazelnuts) in OFC.	Hazelnut-, Cor a 1- and Cor a 8-specific IgE levels were lower in both groups after treatment (no statistical significance). In the treatment group, increased mean IgG4 levels (7.34 allergen units (AU)/mL to 9.84 AU/mL) were observed. However, no statistical significance regarding hazelnut-specific IgG4 levels was found between the groups. Increase in IL-10 levels (from 1.62 pg/mL to 2.24 pg/mL) was seen in the active SLIT group after treatment.	• 3 of 1466 total SLIT doses caused systemic reactions, which appeared during build-up phase and were treated with antihistamines. • Local reactions (mainly oral itching) were observed in 109 of 1466 doses.	No clinical trial number found; study was approved by the ethics committees of the participating hospitals	(271)
**Peanut:** peanut and placebo sublingual drops; active group received crude peanut extract (1:20 w/v) dissolved in 0.2% phenol and 50% - 55% glycerinated saline to maximum peanut protein concentration of 5000 µg/ml; Ara h 2 content was approximately 6% of protein concentration; placebo was glycerinated saline solution	18 subjects (1-11 years): 11 in SLIT group, 7 in placebo group	Randomized, double-blind, placebo-controlled	Treatment started with 0.25 µg peanut protein (initial visit). Doses were taken on a daily basis after escalation. Patients returned every 2 weeks for dose escalation until maintenance daily dose of 2000 µg peanut protein was reached. Dose-escalation for 6 months was followed by 6 months maintenance. Final DBPCFC up to cumulative dose of 2500 mg peanut protein performed after 12 months of treatment.	The primary endpoint was defined as the evaluation of change in the reaction threshold to peanut after SLIT therapy compared to placebo. 11 subjects of the active SLIT group tolerated a cumulative dose of 1710 mg peanut protein in DBPCFC after 12 months of treatment, while 7 subjects in the placebo group tolerated a median cumulative dose of 85 mg.	During the first 4 months, peanut-specific IgE increased significantly in the active group (median level 118.5 kU_A_/L) compared to the baseline (33.5 kU_A_/L) and over the following 8 months decreased again (median level of 31.4 kU_A_/L). After 1 years of treatment, peanut-specific IgG4 levels were significant higher in the active SLIT group (1.12 mg/L), compared to the baseline (0.3 mg/L), however, this was not observed in the placebo group. In the active SLIT group, IL-5 levels decreased significantly during the treatment in the active SLIT group (79 pg/ml) compared to the placebo group (368.9 pg/ml). No significant difference in IL-13, IL-10 and IFN-gamma levels were observed between groups. An increase of Tregs was observed in the active group by the end of the treatment, but did not reach significance.	Adverse events were associated with 11.5% of active SLIT doses and 8.6% of placebo doses. No epinephrine was required any time during the study.	NCT00597727	(272)
**Peanut:** peanut and placebo sublingual drops; allergenic extract from whole non-roasted peanut with 0.5% sodium chloride and 0.54% sodium bicarbonate as aqueous extracts in 50% glycerin; Ara h 2 content = 6% of crude protein; placebo extract was prepared from a glycerinated saline solution with phenol	40 (12-37 years) subjects: 20 in SLIT group, 20 placebo controls	Randomized, double-blind, placebo-controlled	Escalation dosing started with 0.000165 µg peanut protein. Biweekly escalation through 660 µg with 3 doses given at minimal interval of 30 minutes. If participants failed 3 dose escalations after 3 consecutive (biweekly) attempts, 1-2 dose biweekly escalations were allowed. After each escalation, participants continued with daily dose intake until 660 µg target dose was achieved. Subsequently, single dose increase occurred, followed by a 2-week maintenance phase. Maintenance daily doses of 165-1386 µg peanut protein were taken until subjects underwent DBPCFC with 5 g peanut powder in weeks 44. After unblinding at week 44, placebo controls crossed over to higher-dose peanut SLIT (3696 µg maximum maintenance dose), followed by 5 g peanut powder OFC at week 44. Subjects in the original active treatment group continued with maintenance dose followed by 10 g peanut powder OFC after 12 months of maintenance.	After 44 weeks, 14 of 20 (70%) subjects in the SLIT group and 3 of 20 (15%) in the placebo group achieved primary endpoint of tolerating 5 g or at least 10-fold more peanut powder (~2.5 g peanut protein) compared to the baseline in OFC and were considered responders. In the crossover group, 7 of 16 (44%) subjects were considered responders.	In the active SLIT group, median peanut-specific IgE levels increased significantly from baseline to week 44, but not between week 44 and week 68. At week 44, no significant differences in peanut-specific IgE levels were seen between active SLIT and placebo group, SLIT responders and non-responders or high-dose crossover and original peanut SLIT subjects. Peanut-specific IgG4 levels increased significantly between baseline and week 44 in the active SLIT group, which was not observed in the placebo group. Between weeks 44 and 68 no increase was observed in the peanut SLIT group. In the crossover subjects an increase in peanut-specific IgG4 levels was observed from baseline to week 44. No difference in peanut-specific IgG4 levels was observed between treatment responders and non-responders.	In total, 127 of 11854 doses (1.1%) that were given until week 44 required treatment; only one with epinephrine. 59.9% of peanut SLIT doses compared to 99.4% of placebo doses were symptom-free until week 44. In the high-dose crossover group, 66.7% of doses were symptom-free.	NCT00580606	(273)
**Peanut:** peanut extract prepared from the edible part of the peanut with 0.5% sodium chloride and 0.54% sodium bicarbonate as aqueous extracts in 50% glycerin; dose of individual major allergens not determined; glycerinated saline used for placebo	21 (7-13 years) subjects: 10 in active SLIT/placebo OIT group and 11 in active OIT/placebo SLIT group; 16 completed protocol (9 in active SLIT group)	Randomized, double-blinded, placebo-controlled	SLIT treatment started with 0.000165μg of peanut protein with escalation to 0.066μg on the first day. Daily doses were taken for 16 weeks, with dose increase every 1-2 weeks. Build-up phase was continued until maintenance dose of 3.7 mg peanut protein per day (SLIT) reached, followed by 12 months of maintenance and OFC after 6 and 12 months. Subjects that passed OFC (toleration of 5 g peanut powder or at least 10-fold increase) stopped treatment and were rechallenged after 4 weeks. The others continued with 6 months of unblinded treatment. The subjects that reacted during OFC at 12 months to less than 5 g peanut powder continued treatment with OIT added. Finally, subjects underwent OFC with 10 g. Those that passed the challenge, discontinued treatment for 4 weeks and were then rechallenged (sustained unresponsiveness).	7 of originally 10 subjects in active SLIT group achieved 10-fold increase compared to the baseline (primary endpoint) In total, 9 subjects in the SLIT group continued with unblinded phase with active OIT added. Two had to stop OIT build-up due to side effects. The other 7 passed OFC after 6 months of add-on OIT. 1 of 10 in original SLIT group achieved sustained unresponsiveness.	Peanut-specific IgE increased at first and then decrease over time in both groups, however decrease was greater in the OIT group at 6 and 12 months. In the SLIT group, the median peanut-specific IgE increased from 163 kU_A_/L at baseline to 387 kU_A_/L after 6 months of maintenance before slightly decreasing after 12 months (273 kU_A_/L). In the SLIT group, peanut-specific median IgG4 levels increased from 0.9 mgA/L at baseline to 8.5 mgA/L after 12 months.	9% of doses in the SLIT group were associated with adverse reactions. In total, 9 of 10 SLIT subjects had symptoms with dosing. Antihistamines were required in 23.1% of SLIT doses. Epinephrine was required by one subject in the active SLIT/additional OIT group during OIT build-up.	NCT01084174	(255)
**Peanut:** see (273)	40 subjects (12-40 years)	Open-label Follow up study to (273)	Second phase of study by Fleischer et al. (273). Maintenance of peanut SLIT with daily doses of 165-1386 µg peanut protein for 164 weeks. OFCs were performed at 2 and 3 years of SLIT maintenance. Those subjects that passed 10 g peanut powder (5 g peanut protein) OFC were rechallenged after 8 weeks of treatment discontinuation to evaluate sustained unresponsiveness. Sustained unresponsiveness OFC included challenge to 10 g followed by open feeding of 2 tablespoons peanut butter 1 h later.	In higher-dose crossover group, 12 of 17 withdrew prior to final OFC. Of the remaining 5, 2 passed 10 g peanut powder OFC after 3 years and further achieved sustained unresponsiveness. In the initial active peanut SLIT group, 11 of 20 subjects withdrew prior to final OFC. Of the remaining 9, 2 passed OFC after 3 years and further achieved sustained unresponsiveness.	Total IgE levels, peanut-specific IgE and IgG4 levels were not statistically different in those defined as treatment responders (passed OFC) at year 2 and those that were non-responders. Percentage of CD63+ basophils was significantly lower in the 2-year responders than in non-responders.	In the time following the initial 44 weeks, 112 adverse events were reported by 12 high-dose crossover subjects and 83 adverse events were reported by 13 subjects in the original peanut SLIT group. In the peanut SLIT group, 1 life-threatening anaphylactic reaction occurred during year 3 OFC. However, only a mild contact reaction was considered definitely related to the study product.	NCT00580606	(274)
**Peanut:** see study by Kim et al. (272)	48 subjects (1-11 years) initially included: 19 subjects from the initial study, 11 subsequently enrolled subjects and 18 subjects from an additional study cohort that followed identical dosing protocol; 37 subjects completed SLIT therapy	Open-label; extension study of (272)	Initial study was described above (272). An additional cohort of patients that followed identical protocol were also included in the extension study. During the long-term extension study, subjects received SLIT with maintenance daily dose of 2 mg peanut protein (up to 5 years). After the final day of SLIT, sensitization was assessed by DBPCFC with 5 g peanut protein.	12 of 48 (25%) passed challenge with 5000 mg peanut protein without showing clinical symptoms. The 12 subjects discontinued SLIT for 2-4 weeks, were rechallenged and 10 subjects demonstrated sustained unresponsiveness. Overall, 37 of 48 subjects completed the SLIT treatment (9 after 3 years, 1 after 4 years, 27 after 5 years). 32 of 48 (67%) intention-to-treat subjects tolerated at least 750 mg peanut protein during DBPCFC.	The median peanut-specific IgE level decreased significant from baseline (83.9 kU_A_/L) to study completion (20.0 kU_A_/L). Median peanut-specific IgG4 level increased significantly from baseline (0.3 mg/L) to study completion (10.9 mg/L). The peanut-specific IgG4/peanut-specific IgE ration increased from 1.45 at baseline to 356.3. Ratio of peanut-specific basophil activation/non-specific activation decreased significantly.	Of 75,366 total doses, 3599 (4.78%) were associated with side effects affecting 45/48 subjects. During end-of-treatment DBPCFC, 12 subjects required epinephrine. During sustained unresponsiveness DBPCFC no epinephrine treatment was required.	No clinical trial number found; protocol and consent forms approved by the local institutional review board	(275)
**EPIT**								
**Peanut:** Viaskin peanut patch containing liquid formulation of peanut protein extract derived from defatted peanut flour; Viaskin peanut 100 µg (VP100) or 250 µg (VP250) used for treatment; dose of individual major allergens not determined; for placebo same device without peanut protein	74 subjects (4-25 years) started dosing: 24 in VP100 group, 25 in VP250 group, 25 placebo controls	Randomized, double-blind, placebo-controlled	At study entry, subjects underwent OFC with cumulative dose of 1044 mg peanut protein. Participants either received Viaskin Peanut 100 µg or Viaskin Peanut 250 µg. Patch was placed on upper arm (subjects older than 11 years) or the interscapular space (subjects aged 4-11 years). 1-6 application sited were used at 24-h intervals. Doses were increased by extending duration the patch was worn. In the first week, patch was worn 3 h/day, in week 2, 6 h/day and week 3, 12h/day. Patch was applied 24 h/day from day 22 on. At week 52, subjects underwent challenge with cumulative dose of 5044 mg peanut protein.	Primary endpoint was defined by passing week-52 OFC with 5044 mg peanut protein (cumulative) or at least 10-fold increase compared to baseline, which was achieved in 11 of 24 (46%) VP100-treated subjects, 12 of 25 (48%) VP250-treated subjects and 3 of 25 (12%) in the placebo group. Higher treatment response was observed in younger children.	Subjects that received active treatment had increased peanut-specific IgG4 levels and IgG4/IgE ratios compared to the placebo receiving subjects. No difference between treatments was seen for total IgE levels and percentage of peanut-specific IgE over the time of the study. Median frequencies of T cells producing IL-4 and IL-13 were lower at the VP250 dose compared to placebo, but not at the VP100 dose.	14.4% of placebo doses caused an adverse reaction in comparison to 79.8% of VP100 and VP250 doses. Most reactions were mild and occurred at patch site. A patch-site reaction of grade 4 was reported by one patient with VP100 dose at day 34. Reactions not limited to patch site were associated with 0.2% of placebo doses, 0.2% of VP100 doses and 0.1% of VP250 doses. No epinephrine was required with dosing.	NCT01904604	(276)
**Peanut:** Viaskin peanut patch containing liquid formulation of peanut protein extract derived from defatted peanut flour; Viaskin patch (VP) with 50 µg, 100 µg or 250 µg peanut protein used for treatment; dose of individual major allergens not determined; for placebo same device without peanut protein	221 (5-66 years) subjects: 53 in VP50 group, 56 in VP100 group, 56 in VP250 group, 56 in placebo group	Phase 2 double-blind, placebo-controlled dose-ranging study, followed by open-label extension (for 2 years)	Participants received patches containing either 50, 100 or 250 µg peanut protein. The patched were applied daily either on backs (children) or inner upper arms (adolescents and adults). In the first week, patch was worn 3 h/day, in week 2, 6 h/day and week 3, 12h/day. Patch was applied 24 h/day from the third week on. After 12 months, subjects continued with 2-year open-label extension. At 6 months all subjects received 250 µg patch.	Primary endpoint was defined as percentage of treatment responders after 12 months of treatment. Responders reached at least 10-times increase in the eliciting dose and/or at least 1000 mg peanut protein in OFC. This applied for 28 of 56 (50%) in the 250-µg group compared to 14 of 56 (25%) in the placebo group. No statistically significant difference in response rate was observed between the 100-µg group and the placebo control. The highest difference in response rate between the 250-µg group and the placebo group was seen in the age group between 6 and 11 years.	In patch-treated subjects, the median peanut-specific IgE levels increased over the first 3-6 months compared to the placebo group, followed by a decrease reaching almost baseline levels at 12 months. Peanut-specific IgG4 levels increased over the 12-months treatment period in all patch-treated subjects. After 12 months, mean peanut-specific IgG4 levels were greater for VP250 subjects than placebo subjects.	Treatment-emergent adverse events occurred primarily during the first months of therapy and twice as often in the peanut-patch groups compared to the placebo group. Most adverse events were local skin reactions. In total, 20 serious adverse events were reported in 17 subjects, but none of them were treatment-related and most occurred during food challenges.	NCT01675882	(277)
**Peanut:** 250 µg peanut protein-containing patch (Viaskin); dose of individual major allergens not determined; for placebo same device without peanut protein	356 subjects (4-11 years): 238 in peanut-patch group, 118 placebo controls	Randomized, double-blind, placebo-controlled (phase 3 trial)	Daily active treatment with 250 µg peanut protein-containing patch. Treatment responders were defined as those passing OFC after 12 months of treatment by reaching at least 300 mg (for those with baseline eliciting dose of ≤ 10 mg) or at least 1000 mg peanut protein (for those that had baseline eliciting dose of 10-300 mg). On the first day patch was worn 3 h/day, in week 1, 6 h/day (gradually increased) in week 2, 12h/day and thereafter patch was applied 24 h/day.	Primary endpoint was defined by differences in the respond rate between patch-treated and placebo-treated subjects determined by OFC after 12 months of treatment. 84 of 238 (35.3%) of peanut-patch treated subjects compared to 16 of 118 (13.6%) in the placebo group were considered responders.	Not reported	Incidence of treatment-emergent adverse events was 95.4%in the peanut-patch group and 89% in the placebo group. Most adverse reactions occurred at the application site and primarily within the first month. 4 patients in the peanut-patch group experienced adverse events that led to treatment discontinuation. 4.2% of subjects in the peanut-patch group and 5.1% in the placebo group reported serious adverse events at any time during the study (excluding OFC).	NCT02636699	(278)
**Molecular AIT**								
**Peanut:** rectally administered vaccine (EMP-123) consisting of recombinant modified Ara h 1, Ara h 2, and Ara h 3, encapsulated within heat/phenol inactivated *E.* *coli*.	10 peanut-allergic subjects (18-50 years) and 5 healthy subjects	Phase 1 trial	Rectally administration of EMP-123. Five healthy control subjects received 4 weekly escalating doses up to a maximum of 3063 µg modified peanut protein. Peanut-allergic patients received weekly dose escalations for 10 weeks (10-3063 µg), followed by 3 biweekly doses of 3063 µg (maximum dose).	Primary endpoint was defined as assessment of safety of EMP-123 in peanut-allergic subjects and healthy controls. 4 of 10 peanut-allergic patients completed dosing without experiencing symptoms. 1 subjects experienced rectal pruritus, but completed treatment and was considered non-reactive. 2 subjects had mild adverse reactions, 3 experienced more severe side effects, including 2 anaphylactic reactions. In the healthy subject group, 2 experienced diarrhea or loose stools after dosing.	In healthy subjects no immunological changes were observed. No significant changes in peanut-specific IgE levels from baseline to week 20 were observed for reactive and non-reactive subjects, however, baseline peanut- and Ara h 2-specific IgE levels were higher in the 5 reactive subjects. Peanut-specific IgG4 levels did not change significantly from baseline to week 20.	See clinical outcome	No clinical trial number found; study approved by the NIAID Data Safety Monitoring Board, the investigational review boards of Mount Sinai and Johns Hopkins, and the NIH Recombinant DNA Advisory Committee	(279)

Design of studies, numbers of participants, outcomes, side effects and references are listed.

There are methods available for determining major peanut allergens in natural allergen extracts (281) but the precise concentrations of the individual peanut allergens in the natural extracts is not known. Currently, there is no standardized procedure for OIT neither regarding the study design nor are there defined vaccines with known composition. Usually, OIT starts with a dose-escalation day, followed by a buildup phase during which increasing amounts of the allergen are ingested until the maintenance dose is reached. DBPCFC might be performed after a defined food avoidance period to confirm sustained desensitization in the treated subjects. Already in 2009, Jones et al. reported a clinical trial of peanut OIT (249). Since then, the efficacy and safety of peanut OIT have been extensively studied. OIT studies demonstrated successful desensitization and the production of protective IgG4 antibodies but reports of adverse reactions raised safety concerns (267, 269). Adverse reactions affecting the gastrointestinal and respiratory tract during peanut OIT are common (282). To reduce the risk of side effects and to accelerate the desensitization process, the supplementation of OIT with omalizumab, an anti-IgE monoclonal antibody, has been suggested (283–285). The optimal time point to start OIT, treatment duration and length of the maintenance phase are still a matter of debate. With exception of few studies (261, 265, 267–270), most studies involved less than 100 patients and the achieved clinical benefits were relatively modest when put into context with side effects. Accordingly there are different opinions about OIT. One metanalysis (286) concluded: “In patients with peanut allergy, high-certainty evidence shows that available peanut oral immunotherapy regimens considerably increase allergic and anaphylactic reactions over avoidance or placebo, despite effectively inducing desensitization. Safer peanut allergy treatment approaches and rigorous randomized controlled trials that evaluate patient-important outcomes are needed.” whereas another opinion was more optimistic (287). Nevertheless, Aimmune’s peanut OIT has been approved by FDA in the USA and is now marketed as “Palforzia” (https://www.fda.gov/vaccines-blood-biologics/allergenics/palforzia).

### 5.3 Sublingual Immunotherapy

Another possible form of immunotherapy for nut allergy is sublingual immunotherapy, which is given in the form of allergen-containing tablets or drops that must be kept under the tongue. One intention for the development of SLIT was the reduction of side effects and its simplified application for self-administration by the patients. However, clinical effects of SLIT are less pronounced than for SCIT for respiratory allergens (235) and there are only few studies, most of them performed in few patients for nut allergy (**Table 3**) (255, 271–275). Although few studies showed desensitization in some of the participants by the end of the therapy, the results regarding sustained unresponsiveness and long-term compliance are not encouraging (255, 274).

### 5.4 Epicutaneous Immunotherapy

Epicutaneous immunotherapy (EPIT) is a more recent approach which has been developed originally for AIT of respiratory allergy (288) but has now been evaluated also for AIT of peanut allergy (235, 289). EPIT is based on the direct application of an allergen-containing patch on the patient´s skin, similar as it is performed in APT. In theory, EPIT promises a reduced risk of systemic reactions and an uncomplicated application, also for children, due to its non-invasive nature. **Table 3** provides an overview of current EPIT studies for nut allergy (276–278), which, however, is currently limited exclusively to peanut. Moderate success for the treatment of peanut allergy was reported, with one study showing some efficacy in children between 6 and 11 years (277). A review of available data states that “EPIT might induce desensitization in peanut allergy and an increased risk of local adverse events (AEs). These findings should be interpreted with caution owing to the limited study and heterogeneity. More data in the older (children ≥ 12 years and adults) and other allergic diseases are needed” (289). The analysis of systemic peanut allergen-specific IgG responses has shown that epicutaneous allergen administration induces only a very modest production of allergen-specific IgG and mainly specific T cell activation (16).

### 5.5 Molecular Immunotherapy *via* the Subcutaneous Route

As already mentioned above, SCIT has not been developed for AIT of allergy to class I food allergens, most likely because of the risk of inducing anaphylactic side effects when natural allergen extracts are used (244, 245).

Regarding molecular AIT we found only one published study in which peanut allergic subjects had been treated by a molecular form of AIT using recombinant modified Ara h 1, 2 and 3 encapsulated in inactivated *Escherichia coli* (279) (**Table 3**) but half of the subjects (5/10) in this trial experienced adverse reactions, and two of them had anaphylactic reactions.

**Figure 4:**
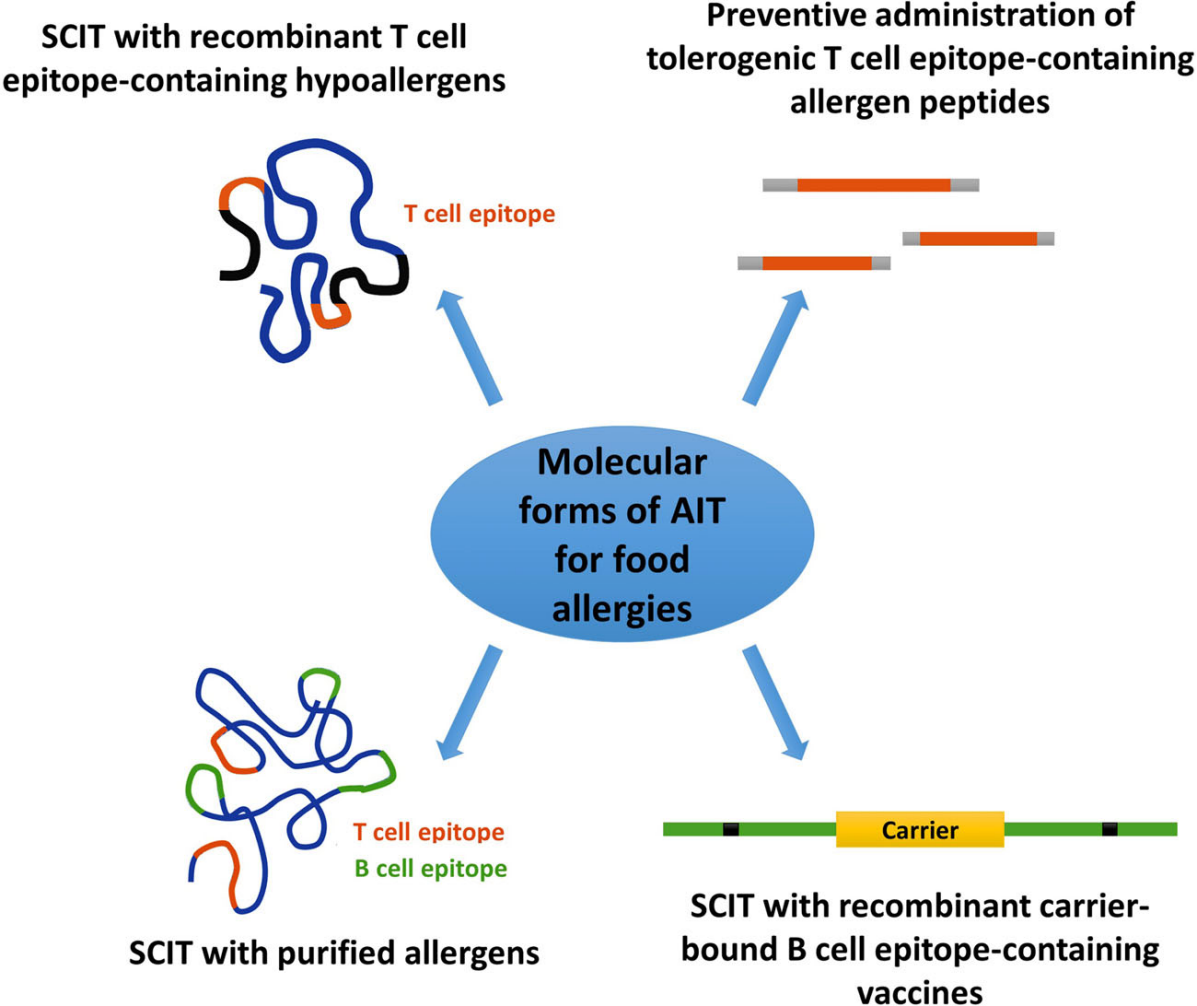
Molecular forms of AIT which can be used for SCIT approaches in nut allergy.

For AIT of respiratory allergy, several molecular AIT approaches have been evaluated already in clinical trials (**Figure 4**), yielding encouraging results in terms of inducing protective IgG responses, alterations of cellular immune responses and evidence for clinical efficacy (235). These approaches include SCIT with recombinant or purified major allergen molecules (290), SCIT based on recombinant hypoallergenic allergen derivatives with (291) and without allergen-specific T cell epitopes (237, 292). For the latter approaches the induction of allergen-specific blocking IgG antibodies has been demonstrated and evidence for clinical efficacy has been obtained. SCIT with allergen-derived T cell epitope-containing peptides has not been successful and an induction of allergen-specific IgG has only been demonstrated when relatively long peptides had been used [reviewed in (235)].

Regarding the development of molecular AIT approaches for treatment of allergy to class I food allergens, important and promising results have been collected for the major fish allergen parvalbumin which such as the major nut allergens represents a digestion-resistant and highly allergenic molecule (293). Within the European Union-funded research program FAST, a hypoallergenic recombinant mutant protein of the major carp allergen Cyp c 1 (294) has been produced, characterized and shown to be hypoallergenic *in vivo* (295–297). Furthermore, safety and ability to induce protective specific IgG responses has been demonstrated in first clinical trials for this molecular vaccine (https://clinicaltrials.gov/: NCT02017626; NCT02382718). Thus it has been proven that it is possible to develop recombinant hypoallergens for SCIT of class I allergens. First recombinant hypoallergenic derivatives of peanut allergens have been characterized in preclinical studies. In fact, several studies reported the production of modified allergen variants of the peanut allergens Ara h 1, Ara h 2 and Ara h 3 and demonstrated reduced IgE reactivity by immunoblotting using patient’s sera (298–300). More recently, the generation of hypoallergenic variants of Ara h 2 and Ara h 6 with decreased allergenic activity but preserved T-cell proliferation capacity has been described (301). Similarly, Tscheppe et al. reported the production of a novel Ara h 2 hypoallergen lacking linear and conformational IgE epitopes (47). IgE reactivity to the unfolded mutant was tested using sera from Ara h 2-sensitized patients and showed reduced IgE-binding capacity compared to natural Ara h 2. The Ara h 2 mutant exhibited low basophil activation ability but still induced T-cell proliferation.

It is known that for allergy to class II food allergens beneficial effects can be obtained by SCIT with the genuinely sensitizing cross-reactive respiratory allergens (40, 302) but the effects on food allergy seem to be lower due to limited cross-reactivity of the induced IgG antibodies (303).

Likewise, molecular AIT with recombinant hypoallergenic birch pollen allergen derivatives was found to induce also cross-protective IgG antibodies to cross-reactive food allergens (41, 291) but similar as for natural allergen extracts, there seems to be limited cross-reactivity of therapy-induced IgG with the cross-reactive food allergens. This has been observed in the clinical trials but also in preclinical studies investigating the cross-protective potential of antibodies induced with molecular vaccines made for the treatment of respiratory allergy (304, 305). Accordingly, it has been suggested to develop recombinant hypoallergens which incorporate also epitopes of the cross-reactive food allergen molecules (306).

### 5.6 Future Molecular Forms of AITs for Nut Allergy: How to Crack the Nut

Originally, recombinant hypoallergenic allergen derivatives have been made to incorporate allergen-specific T cell epitopes but it has been realized that also non-IgE reactive T cell epitopes can cause side effects by activating allergen-specific T cells leading to late phase side effects (192, 307, 308). The more recently developed technology of replacing allergen-specific T cell epitopes by unrelated carrier proteins (309) seems to reduce T cell-mediated side effects and has been shown to yield promising clinical data with approximately 25% improvement of symptoms over placebo when tested for SCIT of grass pollen allergy (237). One may therefore consider the development of carrier-based B cell epitope-containing vaccines by combining peptides derived from the IgE binding sites of the respiratory allergens and the corresponding cross-reactive class II food allergens to obtain combination vaccines for treatment of pollen allergy and the associated oral allergy syndrome (**Figure 4**, lower, right).

The technology of producing fusion proteins consisting of hypoallergenic peptides derived from IgE binding sites of allergens and allergen-unrelated carrier proteins may be applicable also for class I food allergens. However, it needs to be born in mind that it may be more difficult to identify hypoallergenic peptides in class I food allergens because they may harbor not only conformational IgE epitopes which can be easily disrupted but also sequential IgE epitopes of which some may be cryptic (i.e., hidden in the intact allergen structure and exposed only after digestion). It may therefore be difficult to identify non-allergenic peptides derived from the IgE binding sites of class I food allergens which are needed for the construction of the carrier-bound B cell epitope-containing vaccines. SCIT with recombinant purified class I food allergens is in principle possible but vaccines based on purified wild-type allergens may cause severe side effects. SCIT with recombinant T cell epitope-containing hypoallergens derived from class I food allergens seems possible and effective if the vaccines induce allergen-specific protective IgG antibodies but late phase, T cell-mediated side effects may occur. Treatment with T cell epitope-containing peptides from class I food allergens will likely not be successful because short peptides fail to induce protective IgG antibodies but tolerogenic peptides may be considered for preventive approaches (**Figure 4**).

**Figure 5:**
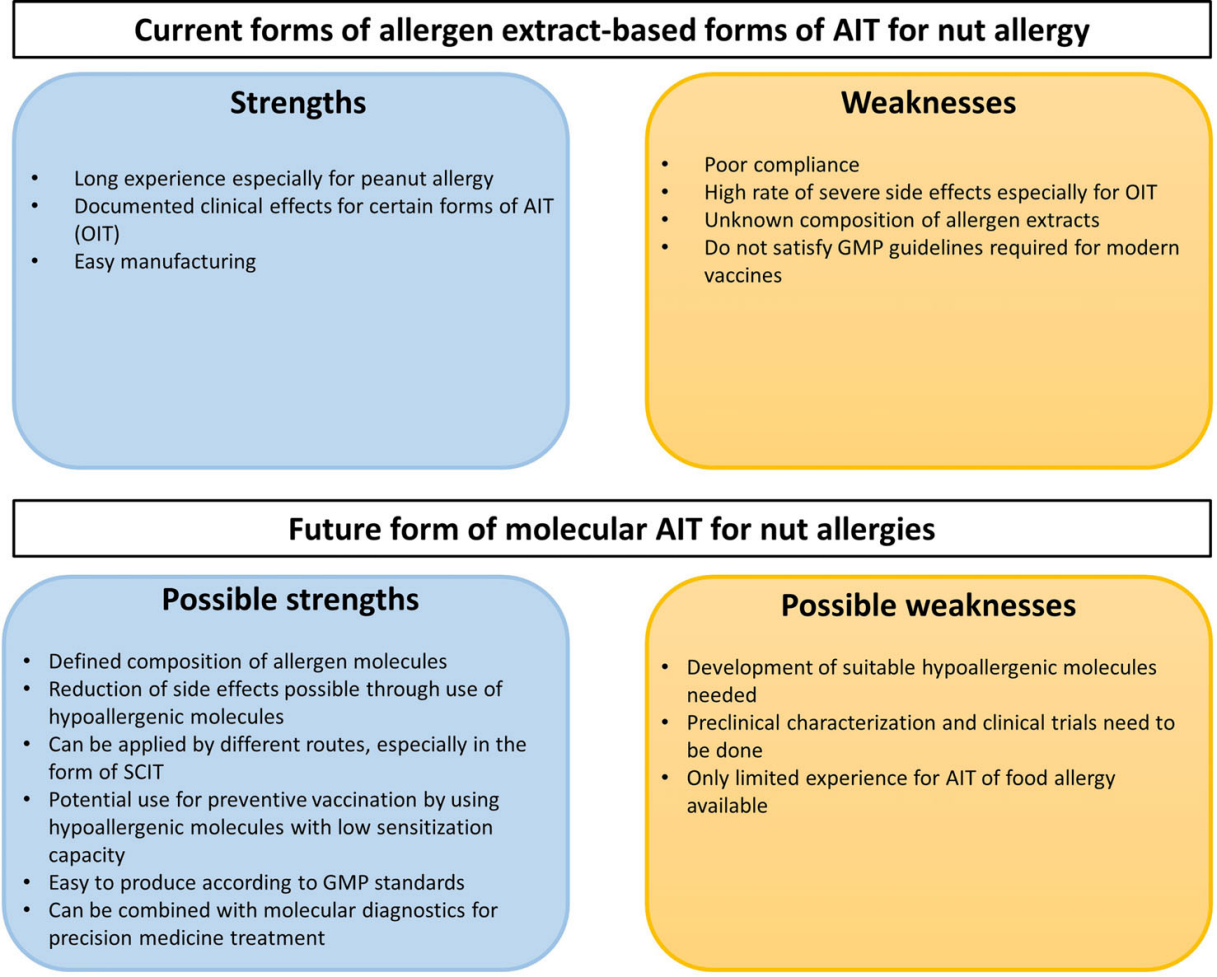
SWOT analysis of existing allergen extract-based forms of AIT for nut allergy and future molecular AIT approaches.

If one performs an analysis of strengths and weaknesses of current allergen extract-based AIT approaches for nut allergy and future molecular AIT vaccines several aspects need to be considered (**Figure 5**). Without doubt, advances have been made regarding the development of allergen extract-based AIT for nut allergy and experience has been collected in several clinical trials (**Figure 5** and **Table 3**). However, the major limitation for allergen extract-based forms of treatment resides in the fact that allergen extracts represent natural products which have major limitations regarding quality, allergen composition, purity and allergenic activity which only can be overcome by introducing molecular approaches for treatment (**Figure 5**) (193). It seems to be due to side effects that SCIT approaches with natural allergen extracts for treating allergy to class I food allergens were not pursued. Instead, mainly OIT approaches have been investigated in larger trials whereas SLIT and EPIT are still in an experimental stage. Side effects are still a concern in OIT with allergen extracts and may be overcome with molecular AIT technologies using hypoallergenic allergen derivatives (**Figure 5**).

Studies performed with molecular AIT approaches indicate high potential but more efforts are needed to advance this treatment into clinical trials and into clinical use. Accordingly, hypoallergenic derivatives need to be developed for the most important allergens, and thus a thorough preclinical and clinical characterization needs to be performed which will require large efforts and investment into the development (**Figure 5**). Most of the experiences have been collected for AIT of respiratory allergy but experience from preclinical and clinical trials in food allergy suggest a common mode of action indicating that SCIT with recombinant nut hypoallergens should be safe, induce protective IgG responses and exhibit clinical efficacy but clinical studies are lacking. Clear advantages of molecular AIT forms are the defined mode of production which satisfies Good Manufacturing Practice requirements needed for clinical studies. A major possible advantage is that molecular design will allow to develop safe and effective forms of AIT for allergy to class I food allergens. Furthermore, molecular AIT can be ideally combined with the already established forms of molecular diagnosis allowing the adequate selection of patients for treatment and also the monitoring of the treatment using molecular biomarkers (209, 236, 310).

**Figure 5** provides a summary of the SWOT analysis of existing allergen extract-based forms of AIT for nut allergy and future forms of molecular AIT but much more needs to be done regarding the preclinical and clinical development of molecular AIT forms for food allergy.

## 6 Summary and Conclusion

Nut allergies might lead to severe allergic reactions or even death, and yet the only current treatment option is avoidance of the allergen source. For AIT as well as in nut allergy diagnosis, extract-based methods are still used. Molecular diagnosis is an alternative to traditional allergen-extract based diagnosis and molecular AIT is a promising future perspective. Molecular AIT approaches require knowledge of molecular sensitization profiles in the population intended to treat. It is evident that currently available studies regarding prevalence of sensitization and allergy to nuts are highly heterogeneous regarding design and only few contain information about molecular sensitization profiles. Therefore, there is a need for molecular studies to obtain comparable data regarding the prevalence of allergy to certain nuts. Molecular IgE-based diagnosis for nut allergy diagnosis may reduce the risk of side effects by reducing the need for provocation tests and promises more comprehensive results. At the moment mainly oral forms of allergen-specific immunotherapy are studied which suffer from poor patients compliance and severe side effects. Molecular AIT is not yet well investigated for treatment of nut allergy although it promises a reduction of side effects through the use of recombinant hypoallergens.

## Author Contributions

RV, VF, and BES wrote the manuscript. RV, VF, and BES designed the figures and tables. VF, MvH, BL, MF-T, IS, OE, AA and MK contributed materials. VF, RV, BES, H-JH, MH, BL, MF-T, IS, OE, MK, MF-T, and AA critically read and revised the manuscript. All authors contributed to the article and approved the submitted version.

## Funding

Supported by the Danube Allergy Research Cluster funded by the Country of Lower Austria, by the MCCA PhD program of the Austrian Science Fund (FWF), by the Russian Academic Excellence Project 5-100, by a Megagrant of the Government of the Russian Federation, grant No 14.W03.31.0024, by a research grant from Worg Pharmaceuticals, Hangzhou, China and by grant from HVD Life Science, Vienna, Austria.

## Conflict of Interest

RV has received research grants from HVD Life Science, Vienna Austria, Viravaxx, Vienna, Austria and Worg Pharmaceuticals, Hangzhou, China and serves as a consultant for Viravaxx and Worg. MvH has received personal fees from Thermo Fisher Scientific, Sweden, and Hycor Biomedical LLC, CA, US., outside the submitted work.

The remaining authors declare that the research was conducted in the absence of any commercial or financial relationships that could be construed as a potential conflict of interest.

## Publisher’s Note

All claims expressed in this article are solely those of the authors and do not necessarily represent those of their affiliated organizations, or those of the publisher, the editors and the reviewers. Any product that may be evaluated in this article, or claim that may be made by its manufacturer, is not guaranteed or endorsed by the publisher.

## Abbreviations

Ig, Immunoglobulin; AIT, Allergen-specific immunotherapy; SCIT, Subcutaneous immunotherapy; PFAS, Pollen-food allergy syndrome; SPT, Skin prick test; DBPCFC, Double-blind placebo-controlled food challenges; LDC, Lip dose challenge; BAT, Basophil activation test; CCD, Cross-reactive carbohydrate determinant; PR-10, Pathogenesis-related class 10; nsLTP, Non-specific lipid transfer protein; OFC, Oral food challenge; APT, Atopy patch test; CRD, Component resolved diagnostics; SLIT, Sublingual immunotherapy; OIT, Oral immunotherapy; EPIT, Epicutaneous immunotherapy.

## References

[1] Blanco Mejia S Kendall CW Viguiliouk E Augustin LS Ha V Cozma AI. Effect of Tree Nuts on Metabolic Syndrome Criteria: A Systematic Review and Meta-Analysis of Randomised Controlled Trials. BMJ Open (2014) 4:e004660. doi: 10.1136/bmjopen-2013-004660 PMC4120343 25074070

[2] Del Gobbo LC Falk MC Feldman R Lewis K Mozaffarian D. Effects of Tree Nuts on Blood Lipids, Apolipoproteins, and Blood Pressure: Systematic Review, Meta-Analysis, and Dose-Response of 61 Controlled Intervention Trials. Am J Clin Nutr (2015) 102:1347–56. doi: 10.3945/ajcn.115.110965 PMC4658458 26561616

[3] Bock SA Muñoz-Furlong A Sampson HA. Fatalities Due to Anaphylactic Reactions to Foods. J Allergy Clin Immunol (2001) 107:191–3. doi: 10.1067/mai.2001.112031 11150011

[4] Bock SA Muñoz-Furlong A Sampson HA. Further Fatalities Caused by Anaphylactic Reactions to Food, 2001-2006. J Allergy Clin Immunol (2007) 119:1016–8. doi: 10.1016/j.jaci.2006.12.622 17306354

[5] Gonzalez-Estrada A Silvers SK Klein A Zell K Wang XF Lang DM. Epidemiology of Anaphylaxis at a Tertiary Care Center: A Report of 730 Cases. Ann Allergy Asthma Immunol (2017) 118:80–5. doi: 10.1016/j.anai.2016.10.025 28007089

[6] Kahveci M Akarsu A Koken G Sahiner UM Soyer O Sekerel BE. Food-Induced Anaphylaxis in Infants, as Compared to Toddlers and Preschool Children in Turkey. Pediatr Allergy Immunol (2020) 31:954–61. doi: 10.1111/pai.13320 32804444

[7] Sicherer SH Burks AW Sampson HA. Clinical Features of Acute Allergic Reactions to Peanut and Tree Nuts in Children. Pediatrics (1998) 102:e6. doi: 10.1542/peds.102.1.e6 9651458

[8] Sicherer SH Furlong TJ DeSimone J Sampson HA. The US Peanut and Tree Nut Allergy Registry: Characteristics of Reactions in Schools and Day Care. J Pediatr (2001) 138:560–5. doi: 10.1067/mpd.2001.111821 11295721

[9] Yu JW Kagan R Verreault N Nicolas N Joseph L St Pierre Y. Accidental Ingestions in Children With Peanut Allergy. J Allergy Clin Immunol (2006) 118:466–72. doi: 10.1016/j.jaci.2006.04.024 16890773

[10] King RM Knibb RC Hourihane JO. Impact of Peanut Allergy on Quality of Life, Stress and Anxiety in the Family. Allergy (2009) 64:461–8. doi: 10.1111/j.1398-9995.2008.01843.x 19076542

[11] McWilliam V Koplin J Lodge C Tang M Dharmage S Allen K. The Prevalence of Tree Nut Allergy: A Systematic Review. Curr Allergy Asthma Rep (2015) 15:54. doi: 10.1007/s11882-015-0555-8 26233427

[12] Han Y Kim J Ahn K. Food Allergy. Korean J Pediatr (2012) 55:153–8. doi: 10.3345/kjp.2012.55.5.153 PMC3362728 22670149

[13] Valenta R Hochwallner H Linhart B Pahr S. Food Allergies: The Basics. Gastroenterology (2015) 148:1120–31.e4. doi: 10.1053/j.gastro.2015.02.006 25680669 PMC4414527

[14] Astwood JD Leach JN Fuchs RL. Stability of Food Allergens to Digestion *In Vitro* . Nat Biotechnol (1996) 14:1269–73. doi: 10.1038/nbt1096-1269 9631091

[15] Brough HA Nadeau KC Sindher SB Alkotob SS Chan S Bahnson HT. Epicutaneous Sensitization in the Development of Food Allergy: What is the Evidence and How can This be Prevented? Allergy (2020) 75:2185–205. doi: 10.1111/all.14304 PMC7494573 32249942

[16] Campana R Moritz K Neubauer A Huber H Henning R Brodie TM. Epicutaneous Allergen Application Preferentially Boosts Specific T Cell Responses in Sensitized Patients. Sci Rep (2017) 7:11657. doi: 10.1038/s41598-017-10278-1 28912492 PMC5599525

[17] Lin J Sampson HA. The Role of Immunoglobulin E-Binding Epitopes in the Characterization of Food Allergy. Curr Opin Allergy Clin Immunol (2009) 9:357–63. doi: 10.1097/ACI.0b013e32832d05ba 19568005

[18] Burks AW Shin D Cockrell G Stanley JS Helm RM Bannon GA. Mapping and Mutational Analysis of the IgE-Binding Epitopes on Ara H 1, a Legume Vicilin Protein and a Major Allergen in Peanut Hypersensitivity. Eur J Biochem (1997) 245:334–9. doi: 10.1111/j.1432-1033.1997.t01-1-00334.x 9151961

[19] Stanley JS King N Burks AW Huang SK Sampson H Cockrell G. Identification and Mutational Analysis of the Immunodominant IgE Binding Epitopes of the Major Peanut Allergen Ara H 2. Arch Biochem Biophys (1997) 342:244–53. doi: 10.1006/abbi.1997.9998 9186485

[20] Stiefel G Anagnostou K Boyle RJ Brathwaite N Ewan P Fox AT. BSACI Guideline for the Diagnosis and Management of Peanut and Tree Nut Allergy. Clin Exp Allergy (2017) 47:719–39. doi: 10.1111/cea.12957 28836701

[21] Cetinkaya PG Buyuktiryaki B Soyer O Sahiner UM Sekerel BE. Factors Predicting Anaphylaxis in Children With Tree Nut Allergies. Allergy Asthma Proc (2019) 40:180–6. doi: 10.2500/aap.2019.40.4211 31018893

[22] Breiteneder H Pettenburger K Bito A Valenta R Kraft D Rumpold H. The Gene Coding for the Major Birch Pollen Allergen Betv1, is Highly Homologous to a Pea Disease Resistance Response Gene. EMBO J (1989) 8:1935–8. doi: 10.1002/j.1460-2075.1989.tb03597.x PMC401053 2571499

[23] Valenta R Duchêne M Pettenburger K Sillaber C Valent P Bettelheim P. Identification of Profilin as a Novel Pollen Allergen; IgE Autoreactivity in Sensitized Individuals. Science (1991) 253:557–60. doi: 10.1126/science.1857985 1857985

[24] Valenta R Duchene M Ebner C Valent P Sillaber C Deviller P. Profilins Constitute a Novel Family of Functional Plant Pan-Allergens. J Exp Med (1992) 175:377–85. doi: 10.1084/jem.175.2.377 PMC2119109 1370681

[25] Valenta R Ferreira F Grote M Swoboda I Vrtala S Duchêne M. Identification of Profilin as an Actin-Binding Protein in Higher Plants. J Biol Chem (1993) 268:22777–81. doi: 10.1016/S0021-9258(18)41594-3 7693678

[26] Valenta R Kraft D. Type I Allergic Reactions to Plant-Derived Food: A Consequence of Primary Sensitization to Pollen Allergens. J Allergy Clin Immunol (1996) 97:893–5. doi: 10.1016/s0091-6749(96)80062-5 8655883

[27] Kim M Ahn Y Yoo Y Kim DK Yang HJ Park HS. Clinical Manifestations and Risk Factors of Anaphylaxis in Pollen-Food Allergy Syndrome. Yonsei Med J (2019) 60:960–8. doi: 10.3349/ymj.2019.60.10.960 PMC6753338 31538431

[28] Reekers R Busche M Wittmann M Kapp A Werfel T. Birch Pollen–Related Foods Trigger Atopic Dermatitis in Patients With Specific Cutaneous T-Cell Responses to Birch Pollen Antigens. J Allergy Clin Immunol (1999) 104:466–72. doi: 10.1016/S0091-6749(99)70395-7 10452773

[29] Wassmann-Otto A Heratizadeh A Wichmann K Werfel T. Birch Pollen-Related Foods can Cause Late Eczematous Reactions in Patients With Atopic Dermatitis. Allergy (2018) 73:2046–54. doi: 10.1111/all.13454 29654628

[30] Letner D Farris A Khalili H Garber J. Pollen-Food Allergy Syndrome is a Common Allergic Comorbidity in Adults With Eosinophilic Esophagitis. Dis Esophagus (2018) 31. doi: 10.1093/dote/dox122 29087472

[31] Spergel J Aceves SS. Allergic Components of Eosinophilic Esophagitis. J Allergy Clin Immunol (2018) 142:1–8. doi: 10.1016/j.jaci.2018.05.001 29980277 PMC6083871

[32] Valenta R Karaulov A Niederberger V Gattinger P van Hage M Flicker S. Molecular Aspects of Allergens and Allergy. Adv Immunol (2018) 138:195–256. doi: 10.1016/bs.ai.2018.03.002 29731005

[33] Vrtala S Hirtenlehner K Vangelista L Pastore A Eichler HG Sperr WR. Conversion of the Major Birch Pollen Allergen, Bet V 1, Into Two Nonanaphylactic T Cell Epitope-Containing Fragments: Candidates for a Novel Form of Specific Immunotherapy. J Clin Invest (1997) 99:1673–81. doi: 10.1172/JCI119330 PMC507987 9120011

[34] Bohle B Zwölfer B Heratizadeh A Jahn-Schmid B Antonia YD Alter M. Cooking Birch Pollen-Related Food: Divergent Consequences for IgE- and T Cell-Mediated Reactivity *In Vitro* and *In Vivo* . J Allergy Clin Immunol (2006) 118:242–9. doi: 10.1016/j.jaci.2006.03.011 16815162

[35] Kazemi-Shirazi L Pauli G Purohit A Spitzauer S Fröschlc R Hoffmann-Sommergruber K. Quantitative IgE Inhibition Experiments With Purified Recombinant Allergens Indicate Pollen-Derived Allergens as the Sensitizing Agents Responsible for Many Forms of Plant Food Allergy. J Allergy Clin Immunol (2000) 105:116–25. doi: 10.1016/S0091-6749(00)90186-6 10629461

[36] Fritsch R Bohle B Vollmann U Wiedermann U Jahn-Schmid B Krebitz M. Bet V 1, the Major Birch Pollen Allergen, and Mal D 1, the Major Apple Allergen, Cross-React at the Level of Allergen-Specific T Helper Cells. J Allergy Clin Immunol (1998) 102:679–86. doi: 10.1016/s0091-6749(98)70287-8 9802379

[37] Westman M Lupinek C Bousquet J Andersson N Pahr S Baar A. Early Childhood IgE Reactivity to Pathogenesis-Related Class 10 Proteins Predicts Allergic Rhinitis in Adolescence. J Allergy Clin Immunol (2015) 135:1199–206.e1-11. doi: 10.1016/j.jaci.2014.10.042 25528361 PMC6597345

[38] Elisyutina O Lupinek C Fedenko E Litovkina A Smolnikov E Ilina N. IgE-Reactivity Profiles to Allergen Molecules in Russian Children With and Without Symptoms of Allergy Revealed by Micro-Array Analysis. Pediatr Allergy Immunol (2021) 32:251–63. doi: 10.1111/pai.13354 PMC7891667 32869350

[39] Elisyutina O Fedenko E Campana R Litovkina A Ilina N Kudlay D. Bet V 1-Specific IgE Levels and PR-10 Reactivity Discriminate Silent Sensitization From Phenotypes of Birch Allergy. Allergy (2019) 74:2525–8. doi: 10.1111/all.13931 PMC6911368 31145475

[40] Bucher X Pichler WJ Dahinden CA Helbling A. Effect of Tree Pollen Specific, Subcutaneous Immunotherapy on the Oral Allergy Syndrome to Apple and Hazelnut. Allergy (2004) 59:1272–6. doi: 10.1111/j.1398-9995.2004.00626.x 15507095

[41] Niederberger V Reisinger J Valent P Krauth MT Pauli G van Hage M. Vaccination With Genetically Modified Birch Pollen Allergens: Immune and Clinical Effects on Oral Allergy Syndrome. J Allergy Clin Immunol (2007) 119:1013–6. doi: 10.1016/j.jaci.2006.12.661 17292956

[42] Boyce JA Assa’ad A Burks AW Jones SM Sampson HA Wood RA. Guidelines for the Diagnosis and Management of Food Allergy in the United States: Report of the NIAID-Sponsored Expert Panel. J Allergy Clin Immunol (2010) 126:S1–58. doi: 10.1016/j.jaci.2010.10.007 21134576 PMC4241964

[43] Upton J Alvaro M Nadeau K. A Perspective on the Pediatric Death From Oral Food Challenge Reported From the Allergy Vigilance Network. Allergy (2019) 74:1035–6. doi: 10.1111/all.13791 30893474

[44] Akarsu A Soyer O Sahiner UM Valenta R Sekerel BE. Improving the Diagnostic Utility of Lip Dose Challenges to Diagnose Tree Nut Allergy. J Allergy Clin Immunol Pract (2021) 9:534–6.e2. doi: 10.1016/j.jaip.2020.08.061 32947027

[45] Matricardi PM Kleine-Tebbe J Hoffmann HJ Valenta R Hilger C Hofmaier S. EAACI Molecular Allergology User’s Guide. Pediatr Allergy Immunol (2016) 27:1–250. doi: 10.1111/pai.12563 27288833

[46] Du Toit G Roberts G Sayre PH Bahnson HT Radulovic S Santos AF. Randomized Trial of Peanut Consumption in Infants at Risk for Peanut Allergy. N Engl J Med (2015) 372:803–13. doi: 10.1056/NEJMoa1414850 PMC4416404 25705822

[47] Tscheppe A Palmberger D van Rijt L Kalic T Mayr V Palladino C. Development of a Novel Ara H 2 Hypoallergen With No IgE Binding or Anaphylactogenic Activity. J Allergy Clin Immunol (2020) 145:229–38. doi: 10.1016/j.jaci.2019.08.036 PMC7100897 31525384

[48] Burney P Summers C Chinn S Hooper R van Ree R Lidholm J. Prevalence and Distribution of Sensitization to Foods in the European Community Respiratory Health Survey: A EuroPrevall Analysis. Allergy (2010) 65:1182–8. doi: 10.1111/j.1398-9995.2010.02346.x 20180791

[49] Osborne NJ Koplin JJ Martin PE Gurrin LC Lowe AJ Matheson MC. Prevalence of Challenge-Proven IgE-Mediated Food Allergy Using Population-Based Sampling and Predetermined Challenge Criteria in Infants. J Allergy Clin Immunol (2011) 127:668–76. doi: 10.1016/j.jaci.2011.01.039 21377036

[50] Burney PG Potts J Kummeling I Mills EN Clausen M Dubakiene R. The Prevalence and Distribution of Food Sensitization in European Adults. Allergy (2014) 69:365–71. doi: 10.1111/all.12341 24372074

[51] Nwaru BI Hickstein L Panesar S Roberts G Muraro A Sheikh A. Prevalence of Common Food Allergies in Europe: A Systematic Review and Meta-Analysis. Allergy (2014) 69:992–1007. doi: 10.1111/all.12423 24816523

[52] Wollmann E Hamsten C Sibanda E Ochome M Focke-Tejkl M Asarnoj A. Natural Clinical Tolerance to Peanut in African Patients is Caused by Poor Allergenic Activity of Peanut IgE. Allergy (2015) 70:638–52. doi: 10.1111/all.12592 PMC6597349 25683061

[53] Venter C Hasan Arshad S Grundy J Pereira B Bernie Clayton C Voigt K. Time Trends in the Prevalence of Peanut Allergy: Three Cohorts of Children From the Same Geographical Location in the UK. Allergy (2010) 65:103–8. doi: 10.1111/j.1398-9995.2009.02176.x 20078504

[54] Hourihane JO Aiken R Briggs R Gudgeon LA Grimshaw KE DunnGalvin A. The Impact of Government Advice to Pregnant Mothers Regarding Peanut Avoidance on the Prevalence of Peanut Allergy in United Kingdom Children at School Entry. J Allergy Clin Immunol (2007) 119:1197–202. doi: 10.1016/j.jaci.2006.12.670 17353036

[55] Nicolaou N Poorafshar M Murray C Simpson A Winell H Kerry G. Allergy or Tolerance in Children Sensitized to Peanut: Prevalence and Differentiation Using Component-Resolved Diagnostics. J Allergy Clin Immunol (2010) 125:191–7.e1-13. doi: 10.1016/j.jaci.2009.10.008 20109746

[56] Luyt DK Vaughan D Oyewole E Stiefel G. Ethnic Differences in Prevalence of Cashew Nut, Pistachio Nut and Almond Allergy. Pediatr Allergy Immunol (2016) 27:651–4. doi: 10.1111/pai.12582 27091782

[57] Du Toit G Katz Y Sasieni P Mesher D Maleki SJ Fisher HR. Early Consumption of Peanuts in Infancy is Associated With a Low Prevalence of Peanut Allergy. J Allergy Clin Immunol (2008) 122:984–91. doi: 10.1016/j.jaci.2008.08.039 19000582

[58] Osterballe M Mortz CG Hansen TK Andersen KE Bindslev-Jensen C. The Prevalence of Food Hypersensitivity in Young Adults. Pediatr Allergy Immunol (2009) 20:686–92. doi: 10.1111/j.1399-3038.2008.00842.x 19594854

[59] Pénard-Morand C Raherison C Kopferschmitt C Caillaud D Lavaud F Charpin D. Prevalence of Food Allergy and its Relationship to Asthma and Allergic Rhinitis in Schoolchildren. Allergy (2005) 60:1165–71. doi: 10.1111/j.1398-9995.2005.00860.x 16076302

[60] Uotila R Kukkonen AK Pelkonen AS Mäkelä MJ. Cross-Sensitization Profiles of Edible Nuts in a Birch-Endemic Area. Allergy (2016) 71:514–21. doi: 10.1111/all.12826 26706253

[61] Mustafayev R Civelek E Orhan F Yüksel H Boz AB Sekerel BE. Similar Prevalence, Different Spectrum: IgE-Mediated Food Allergy Among Turkish Adolescents. Allergol Immunopathol (Madr) (2013) 41:387–96. doi: 10.1016/j.aller.2012.05.005 23036440

[62] Orhan F Karakas T Cakir M Aksoy A Baki A Gedik Y. Prevalence of Immunoglobulin E-Mediated Food Allergy in 6-9-Year-Old Urban Schoolchildren in the Eastern Black Sea Region of Turkey. Clin Exp Allergy (2009) 39:1027–35. doi: 10.1111/j.1365-2222.2009.03263.x 19400894

[63] Kaya A Erkoçoğlu M Civelek E Çakır B Kocabaş CN. Prevalence of Confirmed IgE-Mediated Food Allergy Among Adolescents in Turkey. Pediatr Allergy Immunol (2013) 24:456–62. doi: 10.1111/pai.12097 23772635

[64] Noorbakhsh R Mortazavi SA Sankian M Shahidi F Tehrani M Azad FJ. Pistachio Allergy-Prevalence and *In Vitro* Cross-Reactivity With Other Nuts. Allergol Int (2011) 60:425–32. doi: 10.2332/allergolint.10-OA-0222 21593580

[65] Khazaei HA Hashemi SR Aghamohammadi A Farhoudi F Rezaei N. The Study of Type 1 Allergy Prevalence Among People of South-East of Iran by Skin Prick Test Using Common Allergens. Iran J Allergy Asthma Immunol (2003) 2:165–8. 17301375

[66] Jeong K Lee SY Ahn K Kim J Lee HR Suh DI. A Multicenter Study on Anaphylaxis Caused by Peanut, Tree Nuts, and Seeds in Children and Adolescents. Allergy (2017) 72:507–10. doi: 10.1111/all.13096 27892597

[67] Sun X Zhao J Wang Q Shi G Yang J Ming L. Prevalence of Allergen Sensitization Among 15,534 Patients With Suspected Allergic Diseases in Henan Province, China. Asian Pac J Allergy Immunol (2019) 37:57–64. doi: 10.12932/AP-160817-0137 29602285

[68] Chen J Hu Y Allen KJ Ho MH Li H. The Prevalence of Food Allergy in Infants in Chongqing, China. Pediatr Allergy Immunol (2011) 22:356–60. doi: 10.1111/j.1399-3038.2011.01139.x 21265885

[69] Hu Y Chen J Li H. Comparison of Food Allergy Prevalence Among Chinese Infants in Chongqing, 2009 Versus 1999. Pediatr Int (2010) 52:820–4. doi: 10.1111/j.1442-200X.2010.03166.x 20487367

[70] Lee MP Saffari SE Loh W Goh SH Goh A Chiang WC. A 5-Year Retrospective Review of Children With Peanut Allergy in the Largest Paediatric Hospital in Singapore. Asia Pac Allergy (2020) 10:e6. doi: 10.5415/apallergy.2020.10.e6 32099828 PMC7016321

[71] Shek LP Cabrera-Morales EA Soh SE Gerez I Ng PZ Yi FC. A Population-Based Questionnaire Survey on the Prevalence of Peanut, Tree Nut, and Shellfish Allergy in 2 Asian Populations. J Allergy Clin Immunol (2010) 126:324–31. doi: 10.1016/j.jaci.2010.06.003 20624649

[72] Chiang WC Pons L Kidon MI Liew WK Goh A Burks AW. Serological and Clinical Characteristics of Children With Peanut Sensitization in an Asian Community. Pediatr Allergy Immunol (2010) 21:e429–38. doi: 10.1111/j.1399-3038.2009.00930.x 19702675

[73] Liew WK Chiang WC Goh AE Lim HH Chay OM Chang S. Paediatric Anaphylaxis in a Singaporean Children Cohort: Changing Food Allergy Triggers Over Time. Asia Pac Allergy (2013) 3:29–34. doi: 10.5415/apallergy.2013.3.1.29 23403810 PMC3563018

[74] Cheng CW Lin YC Nong BR Liu PY Huang YF Lu LY. Nut Sensitization Profile in Southern Taiwan. J Microbiol Immunol Infect (2020) 53:791–6. doi: 10.1016/j.jmii.2018.12.005 30683627

[75] Imamura T Kanagawa Y. Ebisawa M. A Survey of Patients With Self-Reported Severe Food Allergies in Japan. Pediatr Allergy Immunol (2008) 19:270–4. doi: 10.1111/j.1399-3038.2007.00621.x 18397411

[76] Sicherer SH Muñoz-Furlong A Godbold JH Sampson HA. US Prevalence of Self-Reported Peanut, Tree Nut, and Sesame Allergy: 11-Year Follow-Up. J Allergy Clin Immunol (2010) 125:1322–6. doi: 10.1016/j.jaci.2010.03.029 20462634

[77] Sicherer SH Muñoz-Furlong A Burks AW Sampson HA. Prevalence of Peanut and Tree Nut Allergy in the US Determined by a Random Digit Dial Telephone Survey. J Allergy Clin Immunol (1999) 103:559–62. doi: 10.1016/s0091-6749(99)70224-1 10200001

[78] Sicherer SH Muñoz-Furlong A Sampson HA. Prevalence of Peanut and Tree Nut Allergy in the United States Determined by Means of a Random Digit Dial Telephone Survey. J Allergy Clin Immunol (2003) 112:1203–7. doi: 10.1016/S0091-6749(03)02026-8 14657884

[79] Ruiz Segura LT Figueroa Pérez E Nowak-Wegrzyn A Siepmann T Larenas-Linnemann D. Food Allergen Sensitization Patterns in a Large Allergic Population in Mexico. Allergol Immunopathol (Madr) (2020) 48:553–9. doi: 10.1016/j.aller.2020.02.004 32444115

[80] Bedolla-Barajas M Bedolla-Pulido TR Macriz-Romero N Morales-Romero J Robles-Figueroa M. Prevalence of Peanut, Tree Nut, Sesame, and Seafood Allergy in Mexican Adults. Rev Invest Clin (2015) 67:379–86. 26950743

[81] Ben-Shoshan M Harrington DW Soller L Fragapane J Joseph L St Pierre Y. A Population-Based Study on Peanut, Tree Nut, Fish, Shellfish, and Sesame Allergy Prevalence in Canada. J Allergy Clin Immunol (2010) 125:1327–35. doi: 10.1016/j.jaci.2010.03.015 20451985

[82] McWilliam V Peters R Tang ML Dharmage S Ponsonby AL Gurrin L. Patterns of Tree Nut Sensitization and Allergy in the First 6 Years of Life in a Population-Based Cohort. J Allergy Clin Immunol (2019) 143:644–650.e5. doi: 10.1016/j.jaci.2018.07.038 30171872

[83] Sasaki M Koplin JJ Dharmage SC Field MJ Sawyer SM McWilliam V. Prevalence of Clinic-Defined Food Allergy in Early Adolescence: The SchoolNuts Study. J Allergy Clin Immunol (2018) 141:391–398.e4. doi: 10.1016/j.jaci.2017.05.041 28755784

[84] Mittermann I Dzoro S Gattinger P Botha M Basera W Facey-Thomas HE. Molecular IgE Sensitization Profiles of Urban and Rural Children in South Africa. Pediatr Allergy Immunol (2021) 32:234–41. doi: 10.1111/pai.13377 32969537

[85] Vetander M Helander D Flodström C Ostblom E Alfvén T Ly DH. Anaphylaxis and Reactions to Foods in Children–a Population-Based Case Study of Emergency Department Visits. Clin Exp Allergy (2012) 42:568–77. doi: 10.1111/j.1365-2222.2011.03954.x 22417215

[86] Soto-Quiros M Gutierrez I Calvo N Araya C Karlberg J Hanson LA. Allergen Sensitization of Asthmatic and Nonasthmatic Schoolchildren in Costa Rica. Allergy (1998) 53:1141–7. doi: 10.1111/j.1398-9995.1998.tb03833.x 9930589

[87] Martínez J Méndez C Talesnik E Campos E Viviani P Sánchez I. Pruebas Cutáneas De Hipersensibilidad Inmediata En Una Población Pediátrica Seleccionada. Rev Med Chil (2005) 133:195–201. doi: 10.4067/s0034-98872005000200007 15824830

[88] Rodríguez-Ortiz PG Muñoz-Mendoza D Arias-Cruz A González-Díaz SN Herrera-Castro D Vidaurri-Ojeda AC. Características Epidemiológicas De Pacientes Con Alergia a Alimentos Atendidos En El Centro Regional De Alergias E Inmunologia Clínica De Monterrey. Rev Alerg Mex (2009) 56:185–91. 20088450

[89] Chen J Liao Y Zhang H Zhao H Chen J Li H. Prevalence of Food Allergy in Children Under 2 Years of Age in Three Cities in China. Zhonghua Er Ke Za Zhi (2012) 50:5–9. 22456067

[90] Lee AJ Thalayasingam M Lee BW. Food Allergy in Asia: How Does it Compare? Asia Pac Allergy (2013) 3:3–14. doi: 10.5415/apallergy.2013.3.1.3 23403837 PMC3563019

[91] Venter C Maslin K Patil V Kurukulaaratchy R Grundy J Glasbey G. The Prevalence, Natural History and Time Trends of Peanut Allergy Over the First 10 Years of Life in Two Cohorts Born in the Same Geographical Location 12 Years Apart. Pediatr Allergy Immunol (2016) 27:804–11. doi: 10.1111/pai.12616 27434312

[92] Kim M Lee JY Jeon HY Yang HK Lee KJ Han Y. Prevalence of Immediate-Type Food Allergy in Korean Schoolchildren in 2015: A Nationwide, Population-Based Study. Allergy Asthma Immunol Res (2017) 9:410–6. doi: 10.4168/aair.2017.9.5.410 PMC5500695 28677354

[93] Peters RL Koplin JJ Gurrin LC Dharmage SC Wake M Ponsonby AL. The Prevalence of Food Allergy and Other Allergic Diseases in Early Childhood in a Population-Based Study: HealthNuts Age 4-Year Follow-Up. J Allergy Clin Immunol (2017) 140:145–153.e8. doi: 10.1016/j.jaci.2017.02.019 28514997

[94] Vereda A van Hage M Ahlstedt S Ibañez MD Cuesta-Herranz J van Odijk J. Peanut Allergy: Clinical and Immunologic Differences Among Patients From 3 Different Geographic Regions. J Allergy Clin Immunol (2011) 127:603–7. doi: 10.1016/j.jaci.2010.09.010 21093026

[95] Asarnoj A Movérare R Ostblom E Poorafshar M Lilja G Hedlin G. IgE to Peanut Allergen Components: Relation to Peanut Symptoms and Pollen Sensitization in 8-Year-Olds. Allergy (2010) 65:1189–95. doi: 10.1111/j.1398-9995.2010.02334.x 20146729

[96] Asarnoj A Hamsten C Lupinek C Melén E Andersson N Anto JM. Prediction of Peanut Allergy in Adolescence by Early Childhood Storage Protein-Specific IgE Signatures: The BAMSE Population-Based Birth Cohort. J Allergy Clin Immunol (2017) 140:587–590.e7. doi: 10.1016/j.jaci.2016.12.973 28192142

[97] Allergen Nomenclature, the Official Site for the Systematic Allergen Nomenclature (2021). Available at: http://www.allergen.org.

[98] Burks AW Cockrell G Stanley JS Helm RM Bannon GA. Recombinant Peanut Allergen Ara H I Expression and IgE Binding in Patients With Peanut Hypersensitivity. J Clin Invest (1995) 96:1715–21. doi: 10.1172/JCI118216 PMC185807 7560062

[99] Clarke MC Kilburn SA Hourihane JO Dean KR Warner JO Dean TP. Serological Characteristics of Peanut Allergy. Clin Exp Allergy (1998) 28:1251–7. doi: 10.1046/j.1365-2222.1998.00386.x 9824392

[100] Kleber-Janke T Crameri R Appenzeller U Schlaak M Becker WM. Selective Cloning of Peanut Allergens, Including Profilin and 2S Albumins, by Phage Display Technology. Int Arch Allergy Immunol (1999) 119:265–74. doi: 10.1159/000024203 10474031

[101] Burks AW Williams LW Connaughton C Cockrell G O’Brien TJ Helm RM. Identification and Characterization of a Second Major Peanut Allergen, Ara *h* II, With Use of the Sera of Patients With Atopic Dermatitis and Positive Peanut Challenge. J Allergy Clin Immunol (1992) 90:962–9. doi: 10.1016/0091-6749(92)90469-i 1460200

[102] Koppelman SJ Wensing M Ertmann M Knulst AC Knol EF. Relevance of Ara H1, Ara H2 and Ara H3 in Peanut-Allergic Patients, as Determined by Immunoglobulin E Western Blotting, Basophil-Histamine Release and Intracutaneous Testing: Ara H2 is the Most Important Peanut Allergen. Clin Exp Allergy (2004) 34:583–90. doi: 10.1111/j.1365-2222.2004.1923.x 15080811

[103] Kukkonen AK Pelkonen AS Mäkinen-Kiljunen S Voutilainen H Mäkelä MJ. Ara H 2 and Ara 6 Are the Best Predictors of Severe Peanut Allergy: A Double-Blind Placebo-Controlled Study. Allergy (2015) 70:1239–45. doi: 10.1111/all.12671 26095653

[104] Valcour A Jones JE Lidholm J Borres MP Hamilton RG. Sensitization Profiles to Peanut Allergens Across the United States. Ann Allergy Asthma Immunol (2017) 119:262–266.e1. doi: 10.1016/j.anai.2017.06.021 28890021

[105] Rabjohn P Helm EM Stanley JS West CM Sampson HA Burks AW. Molecular Cloning and Epitope Analysis of the Peanut Allergen Ara H 3. J Clin Invest (1999) 103:535–42. doi: 10.1172/JCI5349 PMC408104 10021462

[106] Cabanos C Tandang-Silvas Odijk V Brostedt P Tanaka A Utsumi S. Expression, Purification, Cross-Reactivity and Homology Modeling of Peanut Profilin. Protein Expr Purif (2010) 73:36–45. doi: 10.1016/j.pep.2010.03.005 20230899

[107] Schmidt H Krause S Gelhaus C Petersen A Janssen O Becker WM. Detection and Structural Characterization of Natural Ara H 7, the Third Peanut Allergen of the 2S Albumin Family. J Proteome Res (2010) 9:3701–9. doi: 10.1021/pr1002406 20443639

[108] Blankestijn MA Otten HG Suer W Weimann A Knol EF Knulst AC. Specific IgE to Peanut 2S Albumin Ara H 7 has a Discriminative Ability Comparable to Ara H 2 and 6. Clin Exp Allergy (2018) 48:60–5. doi: 10.1111/cea.13030 28906044

[109] Mittag D Akkerdaas J Ballmer-Weber BK Vogel L Wensing M WM B. Ara H 8, a Bet V 1-Homologous Allergen From Peanut, Is a Major Allergen in Patients With Combined Birch Pollen and Peanut Allergy. J Allergy Clin Immunol (2004) 114:1410–7. doi: 10.1016/j.jaci.2004.09.014 15577846

[110] Krause S Reese G Randow S Zennaro D Quaratino D Palazzo P. Lipid Transfer Protein (Ara H 9) as a New Peanut Allergen Relevant for a Mediterranean Allergic Population. J Allergy Clin Immunol (2009) 124:771–778.e5. doi: 10.1016/j.jaci.2009.06.008 19665774

[111] Lauer I Dueringer N Pokoj S Rehm S Zoccatelli G Reese G. The non-Specific Lipid Transfer Protein, Ara H 9, is an Important Allergen in Peanut. Clin Exp Allergy (2009) 39:1427–37. doi: 10.1111/j.1365-2222.2009.03312.x 19624524

[112] Schwager C Kull S Krause S Schocker F Petersen A Becker WM. Development of a Novel Strategy to Isolate Lipophilic Allergens (Oleosins) From Peanuts. PloS One (2015) 10:e0123419. doi: 10.1371/journal.pone.0123419 25860789 PMC4393030

[113] Schwager C Kull S Behrends J Röckendorf N Schocker F Frey A. Peanut Oleosins Associated With Severe Peanut Allergy-Importance of Lipophilic Allergens for Comprehensive Allergy Diagnostics. J Allergy Clin Immunol (2017) 140:1331–1338.e8. doi: 10.1016/j.jaci.2017.02.020 28342912

[114] Petersen A Kull S Rennert S Becker WM Krause S Ernst M. Peanut Defensins: Novel Allergens Isolated From Lipophilic Peanut Extract. J Allergy Clin Immunol (2015) 136:1295–301. doi: 10.1016/j.jaci.2015.04.010 26037551

[115] Teuber SS Dandekar AM Peterson WR Sellers CL. Cloning and Sequencing of a Gene Encoding a 2S Albumin Seed Storage Protein Precursor From English Walnut (Juglans Regia), a Major Food Allergen. J Allergy Clin Immunol (1998) 101:807–14. doi: 10.1016/S0091-6749(98)70308-2 9648708

[116] Costa J Carrapatoso I Oliveira MB Mafra I. Walnut Allergens: Molecular Characterization, Detection and Clinical Relevance. Clin Exp Allergy (2014) 44:319–41. doi: 10.1111/cea.12267 24382327

[117] Teuber SS Jarvis KC Dandekar AM Peterson WR Ansari AA. Identification and Cloning of a Complementary DNA Encoding a Vicilin-Like Proprotein, Jug R 2, From English Walnut Kernel (Juglans Regia), a Major Food Allergen. J Allergy Clin Immunol (1999) 104:1311–20. doi: 10.1016/s0091-6749(99)70029-1 10589017

[118] Pastorello EA Farioli L Pravettoni V Robino AM Scibilia J Fortunato D. Lipid Transfer Protein and Vicilin are Important Walnut Allergens in Patients Not Allergic to Pollen. J Allergy Clin Immunol (2004) 114:908–14. doi: 10.1016/j.jaci.2004.06.020 15480333

[119] Teuber SS Peterson WR Uratsu S Dandekar A Roux KH Sathe SK. Identification and Cloning of Jug R 4, a Major Food Allergen From English Walnut Belonging to the Legumin Group. J Allergy Clin Immunol (2003) 111:S248. doi: 10.1016/S0091-6749(03)80879-5

[120] Wallowitz M Peterson WR Uratsu S Comstock SS Dandekar AM Teuber SS. Jug R 4, a Legumin Group Food Allergen From Walnut (Juglans Regia Cv. Chandler). J Agric Food Chem (2006) 54:8369–75. doi: 10.1021/jf061329s 17032053

[121] Wangorsch A Jamin A Lidholm J Gräni N Lang C Ballmer-Weber B. Identification and Implication of an Allergenic PR-10 Protein From Walnut in Birch Pollen Associated Walnut Allergy. Mol Nutr Food Res (2017) 61. doi: 10.1002/mnfr.201600902 28070926

[122] Dubiela P Kabasser S Smargiasso N Geiselhart S Bublin M Hafner C. Jug R 6 is the Allergenic Vicilin Present in Walnut Responsible for IgE Cross-Reactivities to Other Tree Nuts and Seeds. Sci Rep (2018) 8:11366. doi: 10.1038/s41598-018-29656-4 30054513 PMC6063931

[123] Zhang YZ Du WX Fan Y Yi J Lyu SC Nadeau KC. Purification and Characterization of a Black Walnut (Juglans Nigra) Allergen, Jug N 4. J Agric Food Chem (2017) 65:454–62. doi: 10.1021/acs.jafc.6b04387 27936684

[124] Pastorello EA Vieths S Pravettoni V Farioli L Trambaioli C Fortunato D. Identification of Hazelnut Major Allergens in Sensitive Patients With Positive Double-Blind, Placebo-Controlled Food Challenge Results. J Allergy Clin Immunol (2002) 109:563–70. doi: 10.1067/mai.2002.121946 11898007

[125] Hofmann C Scheurer S Rost K Graulich E Jamin A Foetisch K. Cor a 1-Reactive T Cells and IgE are Predominantly Cross-Reactive to Bet V 1 in Patients With Birch Pollen-Associated Food Allergy to Hazelnut. J Allergy Clin Immunol (2013) 131:1384–92.e6. doi: 10.1016/j.jaci.2012.10.037 23246018

[126] De Knop KJ Verweij MM Grimmelikhuijsen M Philipse E Hagendorens MM Bridts CH. Age-Related Sensitization Profiles for Hazelnut (Corylus Avellana) in a Birch-Endemic Region. Pediatr Allergy Immunol (2011) 22:e139–49. doi: 10.1111/j.1399-3038.2011.01112.x 21342279

[127] Hirschwehr R Valenta R Ebner C Ferreira F Sperr WR Valent P. Identification of Common Allergenic Structures in Hazel Pollen and Hazelnuts: A Possible Explanation for Sensitivity to Hazelnuts in Patients Allergic to Tree Pollen. J Allergy Clin Immunol (1992) 90:927–36. doi: 10.1016/0091-6749(92)90465-e 1281178

[128] Schocker F Lüttkopf D Scheurer S Petersen A Cisteró-Bahima A Enrique E. Recombinant Lipid Transfer Protein Cor a 8 From Hazelnut: A New Tool for *In Vitro* Diagnosis of Potentially Severe Hazelnut Allergy. J Allergy Clin Immunol (2004) 113:141–7. doi: 10.1016/j.jaci.2003.09.013 14713920

[129] Flinterman AE Akkerdaas JH den Hartog Jager CF Rigby NM Fernandez-Rivas M Hoekstra MO. Lipid Transfer Protein-Linked Hazelnut Allergy in Children From a non-Mediterranean Birch-Endemic Area. J Allergy Clin Immunol (2008) 121:423–428.e2. doi: 10.1016/j.jaci.2007.10.009 18036652

[130] Blanc F Bernard H Ah-Leung S Przybylski-Nicaise L Skov PS Purohit A. Further Studies on the Biological Activity of Hazelnut Allergens. Clin Transl Allergy (2015) 5:26. doi: 10.1186/s13601-015-0066-7 26191402 PMC4506444

[131] Beyer K Grishina G Bardina L Grishin A Sampson HA. Identification of an 11S Globulin as a Major Hazelnut Food Allergen in Hazelnut-Induced Systemic Reactions. J Allergy Clin Immunol (2002) 110:517–23. doi: 10.1067/mai.2002.127434 12209105

[132] Ebo DG Verweij MM Sabato V Hagendorens MM Bridts CH De Clerck LS. Hazelnut Allergy: A Multi-Faced Condition With Demographic and Geographic Characteristics. Acta Clin Belg (2012) 67:317–21. doi: 10.2143/ACB.67.5.2062683 23189537

[133] Gruehn S Suphioglu C O’Hehir RE Volkmann D. Molecular Cloning and Characterization of Hazel Pollen Protein (70 Kd) as a Luminal Binding Protein (BiP): A Novel Cross-Reactive Plant Allergen. Int Arch Allergy Immunol (2003) 131:91–100. doi: 10.1159/000070924 12811017

[134] Lauer I Foetisch K Kolarich D Ballmer-Weber BK Conti A Altmann F. Hazelnut (Corylus Avellana) Vicilin Cor a 11: Molecular Characterization of a Glycoprotein and its Allergenic Activity. Biochem J (2004) 383:327–34. doi: 10.1042/BJ20041062 PMC1134074 15233621

[135] Verweij MM Hagendorens MM Trashin S Cucu T De Meulenaer B Devreese B. Age-Dependent Sensitization to the 7S-Vicilin-Like Protein Cor a 11 From Hazelnut (Corylus Avellana) in a Birch-Endemic Region. J Investig Allergol Clin Immunol (2012) 22:245–51. 22812192

[136] Zuidmeer-Jongejan L Fernández-Rivas M Winter MG Akkerdaas JH Summers C Lebens A. Oil Body-Associated Hazelnut Allergens Including Oleosins are Underrepresented in Diagnostic Extracts But Associated With Severe Symptoms. Clin Transl Allergy (2014) 4:4. doi: 10.1186/2045-7022-4-4 24484687 PMC4015814

[137] Garino C Zuidmeer L Marsh J Lovegrove A Morati M Versteeg S. Isolation, Cloning, and Characterization of the 2S Albumin: A New Allergen From Hazelnut. Mol Nutr Food Res (2010) 54:1257–65. doi: 10.1002/mnfr.200900456 20373288

[138] Masthoff LJ Mattsson L Zuidmeer-Jongejan L Lidholm J Andersson K Akkerdaas JH. Sensitization to Cor a 9 and Cor a 14 is Highly Specific for a Hazelnut Allergy With Objective Symptoms in Dutch Children and Adults. J Allergy Clin Immunol (2013) 132:393–9. doi: 10.1016/j.jaci.2013.02.024 23582909

[139] Faber MA De Graag M van der Heijden C Sabato V Hagendorens MM Bridts CH. Cor a 14: Missing Link in the Molecular Diagnosis of Hazelnut Allergy? Int Arch Allergy (2014) 164:200–6. doi: 10.1159/000365050 25034302

[140] Ahn K Bardina L Grishina G Beyer K Sampson HA. Identification of Two Pistachio Allergens, Pis V 1 and Pis V 2, Belonging to the 2S Albumin and 11S Globulin Family. Clin Exp Allergy (2009) 39:926–34. doi: 10.1111/j.1365-2222.2009.03259.x 19522997

[141] Willison LN Tawde P Robotham JM Penney RM Teuber SS Sathe SK. Pistachio Vicilin, Pis V 3, is Immunoglobulin E-Reactive and Cross-Reacts With the Homologous Cashew Allergen, Ana O 1. Clin Exp Allergy (2008) 38:1229–38. doi: 10.1111/j.1365-2222.2008.02998.x 18479490

[142] Ayuso R Grishina G Ahn K Bardina L Beyer K Sampson H. Identification of a MnSOD-Like Protein as a New Major Pistachio Allergen. J Allergy Clin Immunol (2007) 119:S115. doi: 10.1016/j.jaci.2006.11.433

[143] Noorbakhsh R Mortazavi SA Sankian M Shahidi F Assarehzadegan MA Varasteh A. Cloning, Expression, Characterization, and Computational Approach for Cross-Reactivity Prediction of Manganese Superoxide Dismutase Allergen From Pistachio Nut. Allergol Int (2010) 59:295–304. doi: 10.2332/allergolint.10-OA-0174 20567132

[144] Willison LN Sathe SK Roux KH. Production and Analysis of Recombinant Tree Nut Allergens. Methods (2014) 66:34–43. doi: 10.1016/j.ymeth.2013.07.033 23911839

[145] Wang F Robotham JM Teuber SS Tawde P Sathe SK Roux KH. Ana O 1, a Cashew (Anacardium Occidental) Allergen of the Vicilin Seed Storage Protein Family. J Allergy Clin Immunol (2002) 110:160–6. doi: 10.1067/mai.2002.125208 12110836

[146] Wang F Robotham JM Teuber SS Sathe SK Roux KH. Ana O 2, a Major Cashew (Anacardium Occidentale L.) Nut Allergen of the Legumin Family. Int Arch Allergy Immunol (2003) 132:27–39. doi: 10.1159/000073262 14555856

[147] Robotham JM Wang F Seamon V Teuber SS Sathe SK Sampson HA. Ana O 3, an Important Cashew Nut (Anacardium Occidentale L.) Allergen of the 2S Albumin Family. J Allergy Clin Immunol (2005) 115:1284–90. doi: 10.1016/j.jaci.2005.02.028 15940148

[148] Tawde P Venkatesh YP Wang F Teuber SS Sathe SK Roux KH. Cloning and Characterization of Profilin (Pru Du 4), a Cross-Reactive Almond (Prunus Dulcis) Allergen. J Allergy Clin Immunol (2006) 118:915–22. doi: 10.1016/j.jaci.2006.05.028 17030246

[149] Abolhassani M Roux KH. cDNA Cloning, Expression and Characterization of an Allergenic 60s Ribosomal Protein of Almond (Prunus Dulcis). Iran J Allergy Asthma Immunol (2009) 8:77–84. 19671936

[150] Sathe SK Wolf WJ Roux KH Teuber SS Venkatachalam M Sze-Tao KW. Biochemical Characterization of Amandin, the Major Storage Protein in Almond (Prunus Dulcis L.). J Agric Food Chem (2002) 50:4333–41. doi: 10.1021/jf020007v 12105967

[151] Willison LN Tripathi P Sharma G Teuber SS Sathe SK Roux KH. Cloning, Expression and Patient IgE Reactivity of Recombinant Pru Du 6, an 11S Globulin From Almond. Int Arch Allergy Immunol (2011) 156:267–81. doi: 10.1159/000323887 21720172

[152] Kabasser S Hafner C Chinthrajah S Sindher SB Kumar D Kost LE. Identification of Pru Du 6 as a Potential Marker Allergen for Almond Allergy. Allergy (2020) 76:1463–72. doi: 10.1111/all.14613 PMC8247360 33020913

[153] Che H Zhang Y Jiang S Jin T Lyu SC Nadeau KC. Almond (Prunus Dulcis) Allergen Pru Du 8, the First Member of a New Family of Food Allergens. J Agric Food Chem (2019) 67:8626–31. doi: 10.1021/acs.jafc.9b02781 31287307

[154] Pastorello EA Farioli L Pravettoni V Ispano M Conti A Ansaloni R. Sensitization to the Major Allergen of Brazil Nut is Correlated With the Clinical Expression of Allergy. J Allergy Clin Immunol (1998) 102:1021–7. doi: 10.1016/S0091-6749(98)70341-0 9847444

[155] Alcocer MJ Murtagh GJ Bailey K Dumoulin M Meseguer AS Parker MJ. The Disulphide Mapping, Folding and Characterisation of Recombinant Ber E 1, an Allergenic Protein, and SFA8, Two Sulphur-Rich 2S Plant Albumins. J Mol Biol (2002) 324:165–75. doi: 10.1016/S0022-2836(02)01061-6 12421566

[156] Guo F Jin T Howard A Zhang YZ. Purification, Crystallization and Initial Crystallographic Characterization of Brazil-Nut Allergen Ber E 2. Acta Cryst F (2007) 63:976–9. doi: 10.1107/S1744309107051445 PMC2339761 18007055

[157] Beyer K Bardina L Grishina G Ashraf A Teuber S Niggemann B. Identification of a New Brazil Nut Allergen - Ber E 2. J Allergy Clin Immunol (2008) 121:247. doi: 10.1016/j.jaci.2007.12.980

[158] Sharma GM Irsigler A Dhanarajan P Ayuso R Bardina L Sampson HA. Cloning and Characterization of 2S Albumin, Car I 1, a Major Allergen in Pecan. J Agric Food Chem (2011) 59:4130–9. doi: 10.1021/jf104319d 21395309

[159] Zhang Y Lee B Du WX Lyu SC Nadeau KC Grauke LJ. Identification and Characterization of a New Pecan [Carya Illinoinensis (Wangenh.) K. Koch] Allergen, Car I 2. J. Agric Food Chem (2016) 64:4146–51. doi: 10.1021/acs.jafc.6b00884 27128197

[160] Sharma GM Irsigler A Dhanarajan P Ayuso R Bardina L Sampson HA. Cloning and Characterization of an 11S Legumin, Car I 4, a Major Allergen in Pecan. J Agric Food Chem (2011) 59:9542–52. doi: 10.1021/jf2017447 21718052

[161] Morales M López-Matas MÁ Moya R Carnés J. Cross-Reactivity Among non-Specific Lipid-Transfer Proteins From Food and Pollen Allergenic Sources. Food Chem (2014) 165:397–402. doi: 10.1016/j.foodchem.2014.05.101 25038692

[162] Hemmings O Du Toit G Radulovic S Lack G F. Santos A. Ara H 2 is the Dominant Peanut Allergen Despite Similarities With Ara H 6. J Allergy Clin Immunol (2020) 146:621–630.e5. doi: 10.1016/j.jaci.2020.03.026 32298698 PMC7482438

[163] Asarnoj A Glaumann S Elfström L Lilja G Lidholm J Nilsson C. Anaphylaxis to Peanut in a Patient Predominantly Sensitized to Ara H 6. Int Arch Allergy Immunol (2012) 159:209–12. doi: 10.1159/000336027 22677622

[164] Codreanu F Collignon O Roitel O Thouvenot B Sauvage C Vilain A-C. A Novel Immunoassay Using Recombinant Allergens Simplifies Peanut Allergy Diagnosis. Int Arch Allergy Immunol (2011) 154:216–26. doi: 10.1159/000321108 20861643

[165] Ballmer-Weber BK Lidholm J Fernández-Rivas M Seneviratne S Hanschmann KM Vogel L. IgE Recognition Patterns in Peanut Allergy are Age Dependent: Perspectives of the EuroPrevall Study. Allergy (2015) 70:391–407. doi: 10.1111/all.12574 25620497

[166] Maleki SJ Chung SY Champagne ET Raufman JP. The Effects of Roasting on the Allergenic Properties of Peanut Proteins. J Allergy Clin Immunol (2000) 106:763–8. doi: 10.1067/mai.2000.109620 11031348

[167] Beyer K Morrow E Li XM Bardina L Bannon GA Burks A. Effects of Cooking Methods on Peanut Allergenicity. J Allergy Clin Immunol (2001) 107:1077–81. doi: 10.1067/mai.2001.115480 11398088

[168] Mondoulet L Paty E Drumare MF Ah-Leung S Scheinmann P Willemot RM. Influence of Thermal Processing on the Allergenicity of Peanut Proteins. J Agric Food Chem (2005) 53:4547–53. doi: 10.1021/jf050091p 15913323

[169] Vissers YM Blanc F Skov PS Johnson PE Rigby NM Przybylski-Nicaise L. Effect of Heating and Glycation on the Allergenicity of 2S Albumins (Ara H 2/6) From Peanut. PloS One (2011) 6:e23998. doi: 10.1371/journal.pone.0023998 21901150 PMC3162016

[170] Buyuktiryaki B Cavkaytar O Sahiner UM Yilmaz EA Yavuz ST Soyer O. Cor a 14, Hazelnut-Specific IgE, and SPT as a Reliable Tool in Hazelnut Allergy Diagnosis in Eastern Mediterranean Children. J Allergy Clin Immunol Pract (2016) 4:265–72.e3. doi: 10.1016/j.jaip.2015.12.012 26843406

[171] Akkerdaas JH Schocker F Vieths S Versteeg S Zuidmeer L Hefle SL. Cloning of Oleosin, a Putative New Hazelnut Allergen, Using a Hazelnut cDNA Library. Mol Nutr Food Res (2006) 50:18–23. doi: 10.1002/mnfr.200500147 16288502

[172] Datema MR Zuidmeer-Jongejan L Asero R Barreales L Belohlavkova S de Blay F. Hazelnut Allergy Across Europe Dissected Molecularly: A EuroPrevall Outpatient Clinic Survey. J Allergy Clin Immunol (2015) 136:382–91. doi: 10.1016/j.jaci.2014.12.1949 25772593

[173] Costa J Silva I Vicente AA Oliveira M Mafra I. Pistachio Nut Allergy: An Updated Overview. Crit Rev Food Sci Nutr (2019) 59:546–62. doi: 10.1080/10408398.2017.1379947 28925724

[174] Flückiger S Scapozza L Mayer C Blaser K Folkers G Crameri R. Immunological and Structural Analysis of IgE-Mediated Cross-Reactivity Between Manganese Superoxide Dismutases. Int Arch Allergy Immunol (2002) 128:292–303. doi: 10.1159/000063862 12218367

[175] Hasegawa M Inomata N Yamazaki H Morita A Kirino M Ikezawa Z. Clinical Features of Four Cases With Cashew Nut Allergy and Cross-Reactivity Between Cashew Nut and Pistachio. Allergol Int (2009) 58:209–15. doi: 10.2332/allergolint.08-OA-0010 19240380

[176] Salcedo G Sánchez-Monge R Barber D Díaz-Perales A. Plant non-Specific Lipid Transfer Proteins: An Interface Between Plant Defence and Human Allergy. Biochim Biophys Acta (2007) 1771:781–91. doi: 10.1016/j.bbalip.2007.01.001 17349819

[177] Zuidmeer L van Ree R. Lipid Transfer Protein Allergy: Primary Food Allergy or Pollen/Food Syndrome in Some Cases. Curr Opin Allergy Clin Immunol (2007) 7:269–73. doi: 10.1097/ACI.0b013e32814a5401 17489047

[178] Mayer C Appenzeller U Seelbach H Achatz G Oberkofler H Breitenbach M. Humoral and Cell-Mediated Autoimmune Reactions to Human Acidic Ribosomal P2 Protein in Individuals Sensitized to Aspergillus Fumigatus P2 Protein. J Exp Med (1999) 189:1507–12. doi: 10.1084/jem.189.9.1507 PMC2193053 10224291

[179] Roux KH Teuber SS Robotham JM Sathe SK. Detection and Stability of the Major Almond Allergen in Foods. J Agric Food Chem (2001) 49:2131–6. doi: 10.1021/jf001307k 11368566

[180] Venkatachalam M Teuber SS Roux KH Sathe SK. Effects of Roasting, Blanching, Autoclaving, and Microwave Heating on Antigenicity of Almond (Prunus Dulcis L.) Proteins. J Agric Food Chem (2002) 50:3544–8. doi: 10.1021/jf020012z 12033826

[181] Rayes H Raza AA Williams A Matthews S Arshad SH. Specific IgE to Recombinant Protein (Ber E 1) for the Diagnosis of Brazil Nut Allergy. Clin Exp Allergy (2016) 46:654–6. doi: 10.1111/cea.12693 26684696

[182] Sutherland MF O’Hehir RE Czarny D Suphioglu C. Macadamia Nut Anaphylaxis: Demonstration of Specific IgE Reactivity and Partial Cross-Reactivity With Hazelnut. J Allergy Clin Immunol (1999) 104:889–90. doi: 10.1016/S0091-6749(99)70304-0 10518838

[183] Herbst RA Wahl R Frosch PJ. Specific IgE Reactivity and Identification of Potential Allergens in Macadamia Allergy. J Eur Acad Dermatol Venereol (2010) 24:1361–3. doi: 10.1111/j.1468-3083.2010.03642.x 20337822

[184] Ehlers AM Rohwer S Otten HG Brix B Le TM Suer W. IgE-Binding to Vicilin-Like Antimicrobial Peptides is Associated With Systemic Reactions to Macadamia Nut. Clin Transl Allergy (2020) 10:55. doi: 10.1186/s13601-020-00364-5 33292574 PMC7709350

[185] Knott E Gürer CK Ellwanger J Ring J Darsow U. Macadamia Nut Allergy. J Eur Acad Dermatol Venereol (2008) 22:1394–5. doi: 10.1111/j.1468-3083.2008.02657.x 18331311

[186] Ekbote A Hayman G Bansal A. Macadamia Nut Allergy: Potentially Misleading Specific IgE Results. Allergy (2010) 65:1345. doi: 10.1111/j.1398-9995.2010.02354.x 20337625

[187] van Hage M Hamsten C Valenta R. ImmunoCAP Assays: Pros and Cons in Allergology. J Allergy Clin Immunol (2017) 140:974–7. doi: 10.1016/j.jaci.2017.05.008 28552762

[188] Bindslev-Jensen C Ballmer-Weber BK Bengtsson U Blanco C Ebner C Hourihane J. Standardization of Food Challenges in Patients With Immediate Reactions to Foods–Position Paper From the European Academy of Allergology and Clinical Immunology. Allergy (2004) 59:690–7. doi: 10.1111/j.1398-9995.2004.00466.x 15180754

[189] Sampson HA Gerth van Wijk R Bindslev-Jensen C Sicherer S Teuber SS Burks AW. Standardizing Double-Blind, Placebo-Controlled Oral Food Challenges: American Academy of Allergy, Asthma & Immunology-European Academy of Allergy and Clinical Immunology PRACTALL Consensus Report. J Allergy Clin Immunol (2012) 130:1260–74. doi: 10.1016/j.jaci.2012.10.017 23195525

[190] Vazquez-Ortiz M Ludman S Aston A Noimark L Turner PJ. Lip Dose Challenges in Food Allergy: Current Practice and Diagnostic Utility in the United Kingdom. J Allergy Clin Immunol Pract (2019) 7:2770–2774.e3. doi: 10.1016/j.jaip.2019.04.037 31078761 PMC6848915

[191] Ansotegui IJ Melioli G Canonica GW Caraballo L Villa E Ebisawa M. IgE Allergy Diagnostics and Other Relevant Tests in Allergy, a World Allergy Organization Position Paper. World Allergy Organ J (2020) 13:100080. doi: 10.1016/j.waojou.2019.100080 32128023 PMC7044795

[192] Campana R Moritz K Marth K Neubauer A Huber H Henning R. Frequent Occurrence of T Cell-Mediated Late Reactions Revealed by Atopy Patch Testing With Hypoallergenic Rbet V 1 Fragments. J Allergy Clin Immunol (2016) 137:601–609.e8. doi: 10.1016/j.jaci.2015.08.042 26518092 PMC4748398

[193] Valenta R Karaulov A Niederberger V Zhernov Y Elisyutina O Campana R. Allergen Extracts for *In Vivo* Diagnosis and Treatment of Allergy: Is There a Future? J Allergy Clin Immunol Pract (2018) 6:1845–1855.e2. doi: 10.1016/j.jaip.2018.08.032 30297269 PMC6390933

[194] Zivanovic M Atanasković-Marković M Medjo B Gavrović-Jankulović M Smiljanić K Tmušić V. Evaluation of Food Allergy in Children by Skin Prick Tests With Commercial Extracts and Fresh Foods, Specific IgE and, Open Oral Food Challenge-Our Five Years Experience in Food Allergy Work-Up. Iran J Allergy Asthma Immunol (2017) 16:127–32. 28601052

[195] Rancé F Juchet A Brémont F Dutau G. Correlations Between Skin Prick Tests Using Commercial Extracts and Fresh Foods, Specific IgE, and Food Challenges. Allergy (1997) 52:1031–5. doi: 10.1111/j.1398-9995.1997.tb02427.x 9360758

[196] Cetinkaya PG Karaguzel D Esenboğa S Sahiner UM Soyer O Buyuktiryaki B. Pistachio and Cashew Nut Allergy in Childhood: Predictive Factors Towards Development of a Decision Tree. Asian Pac J Allergy Immunol (2021) 39:53–61. doi: 10.12932/AP-281018-0429 31310145

[197] Valenta R Lidholm J Niederberger V Hayek B Kraft D Grönlund H. The Recombinant Allergen-Based Concept of Component-Resolved Diagnostics and Immunotherapy (CRD and CRIT). Clin Exp Allergy (1999) 29:896–904. doi: 10.1046/j.1365-2222.1999.00653.x 10383589

[198] Dang TD Tang M Choo S Licciardi PV Koplin JJ Martin PE. Increasing the Accuracy of Peanut Allergy Diagnosis by Using Ara H 2. J Allergy Clin Immunol (2012) 129:1056–63. doi: 10.1016/j.jaci.2012.01.056 22385632

[199] Klemans RJ Broekman HC Knol EF Bruijnzeel-Koomen CA Otten HG Pasmans SG. Ara H 2 is the Best Predictor for Peanut Allergy in Adults. J Allergy Clin Immunol Pract (2013) 1:632–8.e1. doi: 10.1016/j.jaip.2013.07.014 24565711

[200] Klemans RJ Otte D Knol M Knol EF Meijer Y Gmelig-Meyling FH. The Diagnostic Value of Specific IgE to Ara H 2 to Predict Peanut Allergy in Children is Comparable to a Validated and Updated Diagnostic Prediction Model. J Allergy Clin Immunol (2013) 131:157–63. doi: 10.1016/j.jaci.2012.08.010 23026497

[201] Lieberman JA Glaumann S Batelson S Borres MP Sampson HA Nilsson C. The Utility of Peanut Components in the Diagnosis of IgE-Mediated Peanut Allergy Among Distinct Populations. J Allergy Clin Immunol Pract (2013) 1:75–82. doi: 10.1016/j.jaip.2012.11.002 24229825

[202] Beyer K Grabenhenrich L Härtl M Beder A Kalb B Ziegert M. Predictive Values of Component-Specific IgE for the Outcome of Peanut and Hazelnut Food Challenges in Children. Allergy (2015) 70:90–8. doi: 10.1111/all.12530 25308885

[203] Inoue Y Sato S Takahashi K Yanagida N Yamamoto H Shimizu N. Component-Resolved Diagnostics can be Useful for Identifying Hazelnut Allergy in Japanese Children. Allergol Int (2020) 69:239–45. doi: 10.1016/j.alit.2019.10.001 31680009

[204] Lange L Lasota L Finger A Vlajnic D Büsing S Meister J. Ana O 3-Specific IgE is a Good Predictor for Clinically Relevant Cashew Allergy in Children. Allergy (2017) 72:598–603. doi: 10.1111/all.13050 27644013

[205] van der Valk JP Gerth van Wijk R Vergouwe Y Steyerberg EW Reitsma M Wichers HJ. Sige Ana O 1, 2 and 3 Accurately Distinguish Tolerant From Allergic Children Sensitized to Cashew Nuts. Clin Exp Allergy (2017) 47:113–20. doi: 10.1111/cea.12794 27513566

[206] Sato S Movérare R Ohya Y Ito K Nagao M Borres MP. Ana O 3-Specific IgE is a Predictive Marker for Cashew Oral Food Challenge Failure. J Allergy Clin Immunol Pract (2019) 7:2909–2911.e4. doi: 10.1016/j.jaip.2019.04.049 31108216

[207] Sato S Yamamoto M Yanagida N Ito K Ohya Y Imai T. Jug R 1 Sensitization is Important in Walnut-Allergic Children and Youth. J Allergy Clin Immunol Pract (2017) 5:1784–1786.e1. doi: 10.1016/j.jaip.2017.04.025 28552380

[208] Karsonova A Riabova K Villazala-Merino S Campana R Niederberger V Eckl-Dorna J. Highly Sensitive ELISA-Based Assay for Quantification of Allergen-Specific IgE Antibody Levels. Allergy (2020) 75:2668–70. doi: 10.1111/all.14325 PMC7687237 32302409

[209] Huang HJ Campana R Akinfenwa O Curin M Sarzsinszky E Karsonova A. Microarray-Based Allergy Diagnosis: Quo Vadis? Front Immunol (2020) 11:594978. doi: 10.3389/fimmu.2020.594978 33679689 PMC7928321

[210] Hiller R Laffer S Harwanegg C Huber M Schmidt WM Twardosz A. Microarrayed Allergen Molecules: Diagnostic Gatekeepers for Allergy Treatment. FASEB J (2002) 16:414–6. doi: 10.1096/fj.01-0711fje 11790727

[211] Hoang JA Celik A Lupinek C Valenta R Duan L Dai R. Modeling the Conversion Between Specific IgE Test Platforms for Nut Allergens in Children and Adolescents. Allergy (2020) 76:831–41. doi: 10.1111/all.14529 32738829

[212] Garib V Rigler E Gastager F Campana R Dorofeeva Y Gattinger P. Determination of IgE and IgG Reactivity to More Than 170 Allergen Molecules in Paper-Dried Blood Spots. J Allergy Clin Immunol (2019) 143:437–40. doi: 10.1016/j.jaci.2018.08.047 PMC6392173 30392720

[213] Dubiela P Dölle-Bierke S Aurich S Worm M Hoffmann-Sommergruber K. Component-Resolved Diagnosis in Adult Patients With Food-Dependent Anaphylaxis. World Allergy Organ J (2021) 14:100530. doi: 10.1016/j.waojou.2021.100530 33767803 PMC7973241

[214] Lichtenstein LM Osler AG. Studies on the Mechanisms of Hypersensitivity Phenomena. IX. Histamine Release From Human Leukocytes by Ragweed Pollen Antigen. J Exp Med (1964) 120:507–30. doi: 10.1084/jem.120.4.507 PMC2137776 14212116

[215] Valenta R Sperr W Ferreira F Valent P Sillaber C Tejkl M. Induction of Specific Histamine Release From Basophils With Purified Natural and Recombinant Birch Pollen Allergens. J Allergy Clin Immunol (1993) 91:88–97. doi: 10.1016/0091-6749(93)90300-5 7678614

[216] Hoffmann HJ Santos AF Mayorga C Nopp A Eberlein B Ferrer M. The Clinical Utility of Basophil Activation Testing in Diagnosis and Monitoring of Allergic Disease. Allergy (2015) 70:1393–405. doi: 10.1111/all.12698 26198455

[217] Caraballo L Valenta R Acevedo N Zakzuk J. Are the Terms Major and Minor Allergens Useful for Precision Allergology? Front Immunol (2021) 12:651500. doi: 10.3389/fimmu.2021.651500 33763086 PMC7982392

[218] Mari A Iacovacci P Afferni C Barletta B Tinghino R Di Felice G. Specific IgE to Cross-Reactive Carbohydrate Determinants Strongly Affect the *In Vitro* Diagnosis of Allergic Diseases. J Allergy Clin Immunol (1999) 103:1005–11. doi: 10.1016/S0091-6749(99)70171-5 10359878

[219] Gattinger P Mittermann I Lupinek C Hofer G Keller W Bidovec Stojkovic U. Recombinant Glycoproteins Resembling Carbohydrate-Specific IgE Epitopes From Plants, Venoms and Mites. EBioMedicine (2019) 39:33–43. doi: 10.1016/j.ebiom.2018.12.002 30581149 PMC6354707

[220] Hemmer W Altmann F Holzweber F Gruber C Wantke F Wöhrl S. ImmunoCAP Cellulose Displays Cross-Reactive Carbohydrate Determinant (CCD) Epitopes and can Cause False-Positive Test Results in Patients With High Anti-CCD IgE Antibody Levels. J Allergy Clin Immunol (2018) 141:372–381.e3. doi: 10.1016/j.jaci.2017.04.028 28506851

[221] Altmann F. Coping With Cross-Reactive Carbohydrate Determinants in Allergy Diagnosis. Allergol J Int (2016) 25:98–105. doi: 10.1007/s40629-016-0115-3 PMC5016538 27656353

[222] Cabauatan CR Lupinek C Scheiblhofer S Weiss R Focke-Tejkl M Bhalla PL. Allergen Microarray Detects High Prevalence of Asymptomatic IgE Sensitizations to Tropical Pollen-Derived Carbohydrates. J Allergy Clin Immunol (2014) 133:910–4.e5. doi: 10.1016/j.jaci.2013.10.004 24315449 PMC6597356

[223] Mari A. IgE to Cross-Reactive Carbohydrate Determinants: Analysis of the Distribution and Appraisal of the *In Vivo* and *In Vitro* Reactivity. Int Arch Allergy Immunol (2002) 129:286–95. doi: 10.1159/000067591 12483033

[224] van der Veen MJ van Ree R Aalberse RC Akkerdaas J Koppelman SJ Jansen HM. Poor Biologic Activity of Cross-Reactive IgE Directed to Carbohydrate Determinants of Glycoproteins. J Allergy Clin Immunol (1997) 100:327–34. doi: 10.1016/S0091-6749(97)70245-8 9314344

[225] Hauswirth AW Natter S Ghannadan M Majlesi Y Schernthaner GH Sperr WR. Recombinant Allergens Promote Expression of CD203c on Basophils in Sensitized Individuals. J Allergy Clin Immunol (2002) 110:102–9. doi: 10.1067/mai.2002.125257 12110828

[226] Lötzsch B Dölle S Vieths S Worm M. Exploratory Analysis of CD63 and CD203c Expression in Basophils From Hazelnut Sensitized and Allergic Individuals. Clin Transl Allergy (2016) 6:45. doi: 10.1186/s13601-016-0134-7 27999658 PMC5153676

[227] Kaul S Lüttkopf D Kastner B Vogel L Höltz G Vieths S. Mediator Release Assays Based on Human or Murine Immunoglobulin E in Allergen Standardization. Clin Exp Allergy (2007) 37:141–50. doi: 10.1111/j.1365-2222.2006.02618.x 17210052

[228] Glaumann S Nopp A Johansson SG Rudengren M Borres MP Nilsson C. Basophil Allergen Threshold Sensitivity, CD-Sens, IgE-Sensitization and DBPCFC in Peanut-Sensitized Children. Allergy (2012) 67:242–7. doi: 10.1111/j.1398-9995.2011.02754.x 22126416

[229] Duan L Celik A Hoang JA Schmidthaler K So D Yin X. Basophil Activation Test Shows High Accuracy in the Diagnosis of Peanut and Tree Nut Allergy: The Markers of Nut Allergy Study. Allergy (2021) 76:1800–12. doi: 10.1111/all.14695 PMC8608143 33300157

[230] Patil SU Steinbrecher J Calatroni A Smith N Ma A Ruiter B. Early Decrease in Basophil Sensitivity to Ara H 2 Precedes Sustained Unresponsiveness After Peanut Oral Immunotherapy. J Allergy Clin Immunol (2019) 144:1310–1319.e4. doi: 10.1016/j.jaci.2019.07.028 31377342 PMC6905043

[231] Orgel K Burk C Smeekens J Suber J Hardy L Guo R. Blocking Antibodies Induced by Peanut Oral and Sublingual Immunotherapy Suppress Basophil Activation and are Associated With Sustained Unresponsiveness. Clin Exp Allergy (2019) 49:461–70. doi: 10.1111/cea.13305 PMC6438743 30383313

[232] de Silva D Geromi M Panesar SS Muraro A Werfel T Hoffmann-Sommergruber K. Acute and Long-Term Management of Food Allergy: Systematic Review. Allergy (2014) 69:159–67. doi: 10.1111/all.12314 24215577

[233] Larché M Akdis CA Valenta R. Immunological Mechanisms of Allergen-Specific Immunotherapy. Nat Rev Immunol (2006) 6:761–71. doi: 10.1038/nri1934 16998509

[234] Shamji MH Layhadi JA Sharif H Penagos M Durham SR. Immunological Responses and Biomarkers for Allergen-Specific Immunotherapy Against Inhaled Allergens. J Allergy Clin Immunol Pract (2021) 9:1769–78. doi: 10.1016/j.jaip.2021.03.029 33781958

[235] Dorofeeva Y Shilovskiy I Tulaeva I Focke-Tejkl M Flicker S Kudlay D. Past, Present, and Future of Allergen Immunotherapy Vaccines. Allergy (2020) 76:131–49. doi: 10.1111/all.14300 PMC7818275 32249442

[236] Shamji MH Valenta R Jardetzky T Verhasselt V Durham SR Würtzen PA. The Role of Allergen-Specific IgE, IgG and IgA in Allergic Disease. Allergy (2021). doi: 10.1111/all.14908 PMC8601105 33999439

[237] Niederberger V Neubauer A Gevaert P Zidarn M Worm M Aberer W. Safety and Efficacy of Immunotherapy With the Recombinant B-Cell Epitope-Based Grass Pollen Vaccine BM32. J Allergy Clin Immunol (2018) 142:497–509.e9. doi: 10.1016/j.jaci.2017.09.052 29361332 PMC6392176

[238] Eckl-Dorna J Weber M Stanek V Linhart B Ristl R Waltl EE. Two Years of Treatment With the Recombinant Grass Pollen Allergy Vaccine BM32 Induces a Continuously Increasing Allergen-Specific IgG4 Response. EBioMedicine (2019) 50:421–32. doi: 10.1016/j.ebiom.2019.11.006 PMC6921329 31786130

[239] Orengo JM Radin AR Kamat V Badithe A Ben LH Bennett BL. Treating Cat Allergy With Monoclonal IgG Antibodies That Bind Allergen and Prevent IgE Engagement. Nat Commun (2018) 9:1421. doi: 10.1038/s41467-018-03636-8 29650949 PMC5897525

[240] Gevaert P De Craemer J De Ruyck N Rottey S de Hoon J Hellings PW. Novel Antibody Cocktail Targeting Bet V 1 Rapidly and Sustainably Treats Birch Allergy Symptoms in a Phase 1 Study. J Allergy Clin Immunol (2021) 11:S0091–6749(21)00904–0. doi: 10.1016/j.jaci.2021.05.039 34126156

[241] Shamji MH Singh I Layhadi JA Ito C Karamani A Kouser L. Passive Prophylactic Administration With a Single Dose of Anti-Fel D 1 Monoclonal Antibodies REGN1908-1909 in Cat Allergen-Induced Allergic Rhinitis: A Randomized, Double-Blind, Placebo Controlled Trial. Am J Respir Crit Care Med (2021) 204:23–33. doi: 10.1164/rccm.202011-4107OC 33651675 PMC8437124

[242] Passalacqua G Bagnasco D Canonica GW. 30 Years of Sublingual Immunotherapy. Allergy (2020) 75:1107–20. doi: 10.1111/all.14113 31715001

[243] Kiel MA Röder E Gerth van Wijk R Al MJ Hop WC Rutten-van Mölken MP. Real-Life Compliance and Persistence Among Users of Subcutaneous and Sublingual Allergen Immunotherapy. J Allergy Clin Immunol (2013) 132:353–60.e2. doi: 10.1016/j.jaci.2013.03.013 23651609

[244] Oppenheimer J Nelson H Bock S Christensen F Leung D. Treatment of Peanut Allergy With Rush Immunotherapy. J Allergy Clin Immunol (1992) 90:256–62. doi: 10.1016/0091-6749(92)90080-l 1500630

[245] Nelson H Lahr J Rule R Bock A Leung D. Treatment of Anaphylactic Sensitivity to Peanuts by Immunotherapy With Injections of Aqueous Peanut Extract1. J Allergy Clin Immunol (1997) 99:744–51. doi: 10.1016/S0091-6749(97)80006-1 9215240

[246] Cooper PJ Darbyshire J Nunn AJ Warner JO. A Controlled Trial of Oral Hyposensitization in Pollen Asthma and Rhinitis in Children. Clin Allergy (1984) 14:541–50. doi: 10.1111/j.1365-2222.1984.tb02242.x 6391735

[247] Taudorf E Laursen LC Djurup R Kappelgaard E Pedersen CT Søborg M. Oral Administration of Grass Pollen to Hay Fever Patients. An Efficacy Study in Oral Hyposensitization. Allergy (1985) 40:321–35. doi: 10.1111/j.1398-9995.1985.tb00243.x 3898904

[248] Möller C Dreborg S Lanner A Björkstén B. Oral Immunotherapy of Children With Rhinoconjunctivitis Due to Birch Pollen Allergy. A Double Blind Study. Allergy (1986) 41:271–9. doi: 10.1111/j.1398-9995.1986.tb02028.x 3530030

[249] Jones SM Pons L Roberts JL Scurlock AM Perry TT Kulis M. Clinical Efficacy and Immune Regulation With Peanut Oral Immunotherapy. J Allergy Clin Immunol (2009) 124:292–30197. doi: 10.1016/j.jaci.2009.05.022 19577283 PMC2725434

[250] Blumchen K Ulbricht H Staden U Dobberstein K Beschorner J de Oliveira LC. Oral Peanut Immunotherapy in Children With Peanut Anaphylaxis. J Allergy Clin Immunol (2010) 126:83–91.e1. doi: 10.1016/j.jaci.2010.04.030 20542324

[251] Varshney P Jones SM Scurlock AM Perry TT Kemper A Steele P. A Randomized Controlled Study of Peanut Oral Immunotherapy: Clinical Desensitization and Modulation of the Allergic Response. J Allergy Clin Immunol (2011) 127:654–60. doi: 10.1016/j.jaci.2010.12.1111 PMC3060783 21377034

[252] Anagnostou K Clark A King Y Islam S Deighton J Ewan P. Efficacy and Safety of High-Dose Peanut Oral Immunotherapy With Factors Predicting Outcome. Clin Exp Allergy (2011) 41:1273–81. doi: 10.1111/j.1365-2222.2011.03699.x 21414048

[253] Anagnostou K Islam S King Y Foley L Pasea L Bond S. Assessing the Efficacy of Oral Immunotherapy for the Desensitisation of Peanut Allergy in Children (STOP II): A Phase 2 Randomised Controlled Trial. Lancet (2014) 383:1297–304. doi: 10.1016/S0140-6736(13)62301-6 PMC4255069 24485709

[254] Vickery BP Scurlock AM Kulis M Steele PH Kamilaris J Berglund JP. Sustained Unresponsiveness to Peanut in Subjects Who Have Completed Peanut Oral Immunotherapy. J Allergy Clin Immunol (2014) 133:468–475.e6. doi: 10.1016/j.jaci.2013.11.007 24361082 PMC3960331

[255] Narisety SD Frischmeyer-Guerrerio PA Keet CA Gorelik M Schroeder J Hamilton RG. A Randomized, Double-Blind, Placebo-Controlled Pilot Study of Sublingual Versus Oral Immunotherapy for the Treatment of Peanut Allergy. J Allergy Clin Immunol (2015) 135:1275–82. doi: 10.1016/j.jaci.2014.11.005 PMC4430665 25528358

[256] Bird JA Feldman M Arneson A Dougherty I Brown LS Burk CM. Modified Peanut Oral Immunotherapy Protocol Safely and Effectively Induces Desensitization. J Allergy Clin Immunol Pract (2015) 3:433–5. doi: 10.1016/j.jaip.2014.11.020 25609341

[257] Tang ML Ponsonby AL Orsini F Tey D Robinson M Su EL. Administration of a Probiotic With Peanut Oral Immunotherapy: A Randomized Trial. J Allergy Clin Immunol (2015) 135:737–44.e8. doi: 10.1016/j.jaci.2014.11.034 25592987

[258] Kukkonen AK Uotila R Malmberg LP Pelkonen AS Mäkelä MJ. Double-Blind Placebo-Controlled Challenge Showed That Peanut Oral Immunotherapy was Effective for Severe Allergy Without Negative Effects on Airway Inflammation. Acta Paediatr (2017) 106:274–81. doi: 10.1111/apa.13668 27859599

[259] Vickery BP Berglund JP Burk CM Fine JP Kim EH Kim JI. Early Oral Immunotherapy in Peanut-Allergic Preschool Children is Safe and Highly Effective. J Allergy Clin Immunol (2017) 139:173–181.e8. doi: 10.1016/j.jaci.2016.05.027 27522159 PMC5222765

[260] Bird JA Spergel JM Jones SM Rachid R Assa’ad AH Wang J. Efficacy and Safety of AR101 in Oral Immunotherapy for Peanut Allergy: Results of ARC001, a Randomized, Double-Blind, Placebo-Controlled Phase 2 Clinical Trial. J Allergy Clin Immunol Pract (2018) 6:476–485.e3. doi: 10.1016/j.jaip.2017.09.016 29092786

[261] PALISADE Group Vickery BP Vereda A Casale TB Beyer K Du Toit G. AR101 Oral Immunotherapy for Peanut Allergy. N Engl J Med (2018) 379:1991–2001. doi: 10.1056/NEJMoa1812856 30449234

[262] Nagakura KI Yanagida N Sato S Nishino M Asaumi T Ogura K. Low-Dose Oral Immunotherapy for Children With Anaphylactic Peanut Allergy in Japan. Pediatr Allergy Immunol (2018) 29:512–8. doi: 10.1111/pai.12898 29603410

[263] Fauquert JL Michaud E Pereira B Bernard L Gourdon-Dubois N Rouzaire PO. Peanut Gastrointestinal Delivery Oral Immunotherapy in Adolescents: Results of the Build-Up Phase of a Randomized, Double-Blind, Placebo-Controlled Trial (PITA Study). Clin Exp Allergy (2018) 48:862–74. doi: 10.1111/cea.13148 29665158

[264] Blumchen K Trendelenburg V Ahrens F Gruebl A Hamelmann E Hansen G. Efficacy, Safety, and Quality of Life in a Multicenter, Randomized, Placebo-Controlled Trial of Low-Dose Peanut Oral Immunotherapy in Children With Peanut Allergy. J Allergy Clin Immunol Pract (2019) 7:479–491.e10. doi: 10.1016/j.jaip.2018.10.048 30423449

[265] Wasserman RL Hague AR Pence DM Sugerman RW Silvers SK Rolen JG. Real-World Experience With Peanut Oral Immunotherapy: Lessons Learned From 270 Patients. J Allergy Clin Immunol Pract (2019) 7:418–426.e4. doi: 10.1016/j.jaip.2018.05.023 29859333

[266] Elizur A Appel MY Nachshon L Levy MB Epstein-Rigbi N Pontoppidan B. Walnut Oral Immunotherapy for Desensitisation of Walnut and Additional Tree Nut Allergies (Nut CRACKER): A Single-Centre, Prospective Cohort Study. Lancet Child Adolesc Health (2019) 3:312–21. doi: 10.1016/S2352-4642(19)30029-X 30926371

[267] Chinthrajah RS Purington N Andorf S Long A O’Laughlin KL Lyu SC. Sustained Outcomes in a Large Double-Blind, Placebo-Controlled, Randomized Phase 2 Study of Peanut Immunotherapy. Lancet (2019) 394:1437–49. doi: 10.1016/S0140-6736(19)31793-3 PMC6903389 31522849

[268] Moraly T Pelletier de Chambure D Verdun S Preda C Seynave M Vilain AC. Oral Immunotherapy for Hazelnut Allergy: A Single-Center Retrospective Study on 100 Patients. J Allergy Clin Immunol Pract (2020) 8:704–709.e4. doi: 10.1016/j.jaip.2019.10.045 31751759

[269] Hourihane JO Beyer K Abbas A Fernández-Rivas M Turner PJ Blumchen K. Efficacy and Safety of Oral Immunotherapy With AR101 in European Children With a Peanut Allergy (ARTEMIS): A Multicentre, Double-Blind, Randomised, Placebo-Controlled Phase 3 Trial. Lancet Child Adolesc Health (2020) 4:728–39. doi: 10.1016/S2352-4642(20)30234-0 32702315

[270] Vickery BP Vereda A Nilsson C Du Toit G Shreffler WG Burks AW. Continuous and Daily Oral Immunotherapy for Peanut Allergy: Results From a 2-Year Open-Label Follow-on Study. J Allergy Clin Immunol Pract (2021) 9:1879–1889.e14. doi: 10.1016/j.jaip.2020.12.029 33359589

[271] Enrique E Pineda F Malek T Bartra J Basagaña M Tella R. Sublingual Immunotherapy for Hazelnut Food Allergy: A Randomized, Double-Blind, Placebo-Controlled Study With a Standardized Hazelnut Extract. J Allergy Clin Immunol (2005) 116:1073–9. doi: 10.1016/j.jaci.2005.08.027 16275379

[272] Kim EH Bird JA Kulis M Laubach S Pons L Shreffler W. Sublingual Immunotherapy for Peanut Allergy: Clinical and Immunologic Evidence of Desensitization. J Allergy Clin Immunol (2011) 127:640–646.e1. doi: 10.1016/j.jaci.2010.12.1083 21281959 PMC3052379

[273] Fleischer DM Burks AW Vickery BP Scurlock AM Wood RA Jones SM. Sublingual Immunotherapy for Peanut Allergy: A Randomized, Double-Blind, Placebo-Controlled Multicenter Trial. J Allergy Clin Immunol (2013) 131:119–127.e7. doi: 10.1016/j.jaci.2012.11.011 23265698 PMC3550002

[274] Burks AW Wood RA Jones SM Sicherer SH Fleischer DM Scurlock AM. Sublingual Immunotherapy for Peanut Allergy: Long-Term Follow-Up of a Randomized Multicenter Trial. J Allergy Clin Immunol (2015) 135:1240–8. doi: 10.1016/j.jaci.2014.12.1917 PMC4527157 25656999

[275] Kim EH Yang L Ye P Guo R Li Q Kulis MD. Long-Term Sublingual Immunotherapy for Peanut Allergy in Children: Clinical and Immunologic Evidence of Desensitization. J Allergy Clin Immunol (2019) 144:1320–1326.e1. doi: 10.1016/j.jaci.2019.07.030 31493887 PMC6842439

[276] Jones SM Sicherer SH Burks AW Leung DY Lindblad RW Dawson P. Epicutaneous Immunotherapy for the Treatment of Peanut Allergy in Children and Young Adults. J Allergy Clin Immunol (2017) 139:1242–1252.e9. doi: 10.1016/j.jaci.2016.08.017 28091362 PMC8609774

[277] Sampson HA Shreffler WG Yang WH Sussman GL Brown-Whitehorn TF Nadeau KC. Effect of Varying Doses of Epicutaneous Immunotherapy *vs.* Placebo on Reaction to Peanut Protein Exposure Among Patients With Peanut Sensitivity: A Randomized Clinical Trial. JAMA (2017) 318:1798–809. doi: 10.1001/jama.2017.16591 PMC5820709 29136445

[278] Fleischer DM Greenhawt M Sussman G Bégin P Nowak-Wegrzyn A Petroni D. Effect of Epicutaneous Immunotherapy *vs.* Placebo on Reaction to Peanut Protein Ingestion Among Children With Peanut Allergy: The PEPITES Randomized Clinical Trial. JAMA (2019) 321:946–55. doi: 10.1001/jama.2019.1113 PMC6439674 30794314

[279] Wood RA Sicherer SH Burks AW Grishin A Henning AK Lindblad R. A Phase 1 Study of Heat/Phenol-Killed, E. Coli-Encapsulated, Recombinant Modified Peanut Proteins Ara H 1, Ara H 2, and Ara H 3 (EMP-123) for the Treatment of Peanut Allergy. Allergy (2013) 68:803–8. doi: 10.1111/all.12158 PMC3663889 23621498

[280] Andorf S Purington N Block WM Long AJ Tupa D Brittain E. Anti-IgE Treatment With Oral Immunotherapy in Multifood Allergic Participants: A Double-Blind, Randomised, Controlled Trial. Lancet Gastroenterol Hepatol (2018) 3:85–94. doi: 10.1016/S2468-1253(17)30392-8 29242014 PMC6944204

[281] Berglund JP Szczepanski N Penumarti A Beavers A Kesselring J Orgel K. Preparation and Analysis of Peanut Flour Used in Oral Immunotherapy Clinical Trials. J Allergy Clin Immunol Pract (2017) 5:1098–104. doi: 10.1016/j.jaip.2016.11.034 PMC5503789 28132800

[282] Hofmann AM Scurlock AM Jones SM Palmer KP Lokhnygina Y Steele PH. Safety of a Peanut Oral Immunotherapy Protocol in Children With Peanut Allergy. J Allergy Clin Immunol (2009) 124:286–91. doi: 10.1016/j.jaci.2009.03.045 PMC2731305 19477496

[283] Schneider LC Rachid R LeBovidge J Blood E Mittal M Umetsu DT. A Pilot Study of Omalizumab to Facilitate Rapid Oral Desensitization in High-Risk Peanut-Allergic Patients. J Allergy Clin Immunol (2013) 132:1368–74. doi: 10.1016/j.jaci.2013.09.046 PMC4405160 24176117

[284] MacGinnitie AJ Rachid R Gragg H Little SV Lakin P Cianferoni A. Omalizumab Facilitates Rapid Oral Desensitization for Peanut Allergy. J Allergy Clin Immunol (2017) 139:873–881.e8. doi: 10.1016/j.jaci.2016.08.010 27609658 PMC5369605

[285] Yee CS Albuhairi S Noh E El-Khoury K Rezaei S Abdel-Gadir A. Long-Term Outcome of Peanut Oral Immunotherapy Facilitated Initially by Omalizumab. J Allergy Clin Immunol Pract (2019) 7:451–461.e7. doi: 10.1016/j.jaip.2018.09.015 30267889

[286] Chu DK Wood RA French S Fiocchi A Jordana M Waserman S. Oral Immunotherapy for Peanut Allergy (PACE): A Systematic Review and Meta-Analysis of Efficacy and Safety. Lancet (2019) 393:2222–32. doi: 10.1016/S0140-6736(19)30420-9 31030987

[287] Eiwegger T Anagnostou K Arasi S Bégin P Ben-Shoshan M Beyer K. Conflicting Verdicts on Peanut Oral Immunotherapy From the Institute for Clinical and Economic Review and US Food and Drug Administration Advisory Committee: Where do We Go From Here? J Allergy Clin Immunol (2020) 145:1153–6. doi: 10.1016/j.jaci.2019.10.021 31678426

[288] Senti G Graf N Haug S Rüedi N von Moos S Sonderegger T. Epicutaneous Allergen Administration as a Novel Method of Allergen-Specific Immunotherapy. J Allergy Clin Immunol (2009) 124:997–1002. doi: 10.1016/j.jaci.2009.07.019 19733905

[289] Xiong L Lin J Luo Y Chen W Dai J. The Efficacy and Safety of Epicutaneous Immunotherapy for Allergic Diseases: A Systematic Review and Meta-Analysis. Int Arch Allergy Immunol (2020) 181:170–82. doi: 10.1159/000504366 31801149

[290] Pauli G Larsen TH Rak S Horak F Pastorello E Valenta R. Efficacy of Recombinant Birch Pollen Vaccine for the Treatment of Birch-Allergic Rhinoconjunctivitis. J Allergy Clin Immunol (2008) 122:951–60. doi: 10.1016/j.jaci.2008.09.017 19000581

[291] Niederberger V Horak F Vrtala S Spitzauer S Krauth MT Valent P. Vaccination With Genetically Engineered Allergens Prevents Progression of Allergic Disease. Proc Natl Acad Sci U.S.A. (2004) 101 Suppl 2:14677–82. doi: 10.1073/pnas.0404735101 PMC521981 15310844

[292] Zieglmayer P Focke-Tejkl M Schmutz R Lemell P Zieglmayer R Weber M. Mechanisms, Safety and Efficacy of a B Cell Epitope-Based Vaccine for Immunotherapy of Grass Pollen Allergy. EBioMedicine (2016) 11:43–57. doi: 10.1016/j.ebiom.2016.08.022 27650868 PMC5049999

[293] Swoboda I Bugajska-Schretter A Verdino P Keller W Sperr WR Valent P. Recombinant Carp Parvalbumin, the Major Cross-Reactive Fish Allergen: A Tool for Diagnosis and Therapy of Fish Allergy. J Immunol (2002) 168:4576–84. doi: 10.4049/jimmunol.168.9.4576 11971005

[294] Swoboda I Bugajska-Schretter A Linhart B Verdino P Keller W Schulmeister U. A Recombinant Hypoallergenic Parvalbumin Mutant for Immunotherapy of IgE-Mediated Fish Allergy. J Immunol (2007) 178:6290–6. doi: 10.4049/jimmunol.178.10.6290 17475857

[295] Douladiris N Linhart B Swoboda I Gstöttner A Vassilopoulou E Stolz F. *In Vivo* Allergenic Activity of a Hypoallergenic Mutant of the Major Fish Allergen Cyp C 1 Evaluated by Means of Skin Testing. J Allergy Clin Immunol (2015) 136:493–5.e8. doi: 10.1016/j.jaci.2015.01.015 25746971 PMC6597366

[296] Zuidmeer-Jongejan L Huber H Swoboda I Rigby N Versteeg SA Jensen BM. Development of a Hypoallergenic Recombinant Parvalbumin for First-in-Man Subcutaneous Immunotherapy of Fish Allergy. Int Arch Allergy Immunol (2015) 166:41–51. doi: 10.1159/000371657 25765512

[297] Zuidmeer-Jongejan L Fernandez-Rivas M Poulsen LK Neubauer A Asturias J Blom L. FAST: Towards Safe and Effective Subcutaneous Immunotherapy of Persistent Life-Threatening Food Allergies. Clin Transl Allergy (2012) 2:5. doi: 10.1186/2045-7022-2-5 22409908 PMC3386014

[298] King N Helm R Stanley JS Vieths S Lüttkopf D Hatahet L. Allergenic Characteristics of a Modified Peanut Allergen. Mol Nutr Food Res (2005) 49:963–71. doi: 10.1002/mnfr.200500073 16189800

[299] Rabjohn P West CM Connaughton C Sampson HA Helm RM Burks AW. Modification of Peanut Allergen Ara H 3: Effects on IgE Binding and T Cell Stimulation. Int Arch Allergy Immunol (2002) 128:15–23. doi: 10.1159/000057999 12037397

[300] Bannon GA Cockrell G Connaughton C West CM Helm R Stanley JS. Engineering, Characterization and *In Vitro* Efficacy of the Major Peanut Allergens for Use in Immunotherapy. Int Arch Allergy Immunol (2001) 124:70–2. doi: 10.1159/000053672 11306930

[301] Bublin M Kostadinova M Radauer C Varga EM Hafner C Schmidthaler K. Engineering of Structural Variants of the Major Peanut Allergens Ara H 2 and Ara H 6 for Allergen-Specific Immunotherapy. J Allergy Clin Immunol (2019) 143:1226–1229.e10. doi: 10.1016/j.jaci.2018.10.039 30414861

[302] Mauro M Russello M Incorvaia C Gazzola G Frati F Moingeon P. Birch-Apple Syndrome Treated With Birch Pollen Immunotherapy. Int Arch Allergy Immunol (2011) 156:416–22. doi: 10.1159/000323909 21832831

[303] Gadermaier E Flicker S Aberer W Egger C Reider N Focke M. Analysis of the Antibody Responses Induced by Subcutaneous Injection Immunotherapy With Birch and Fagales Pollen Extracts Adsorbed Onto Aluminum Hydroxide. Int Arch Allergy Immunol (2010) 151:17–27. doi: 10.1159/000232567 19672093

[304] Marth K Breyer I Focke-Tejkl M Blatt K Shamji MH Layhadi J. A Nonallergenic Birch Pollen Allergy Vaccine Consisting of Hepatitis PreS–fused Bet V 1 Peptides Focuses Blocking IgG Toward IgE Epitopes and Shifts Immune Responses to a Tolerogenic and Th1 Phenotype. J Immunol (2013) 190:3068–78. doi: 10.4049/jimmunol.1202441 PMC4148560 23440415

[305] Campana R Vrtala S Maderegger B Jertschin P Stegfellner G Swoboda I. Hypoallergenic Derivatives of the Major Birch Pollen Allergen Bet V 1 Obtained by Rational Sequence Reassembly. J Allergy Clin Immunol (2010) 126:1024–31. doi: 10.1016/j.jaci.2010.05.023 20638112

[306] Hofer H Asam C Hauser M Nagl B Laimer J Himly M. Tackling Bet V 1 and Associated Food Allergies With a Single Hybrid Protein. J Allergy Clin Immunol (2017) 140:525–533.e10. doi: 10.1016/j.jaci.2016.09.055 27939703 PMC5693327

[307] Purohit A Niederberger V Kronqvist M Horak F Grönneberg R Suck R. Clinical Effects of Immunotherapy With Genetically Modified Recombinant Birch Pollen Bet V 1 Derivatives. Clin Exp Allergy (2008) 38:1514–25. doi: 10.1111/j.1365-2222.2008.03042.x 18564326

[308] Haselden BM Kay AB Larché M. Immunoglobulin E-Independent Major Histocompatibility Complex-Restricted T Cell Peptide Epitope-Induced Late Asthmatic Reactions. J Exp Med (1999) 189:1885–94. doi: 10.1084/jem.189.12.1885 PMC2192970 10377184

[309] Valenta R Campana R Niederberger V. Recombinant Allergy Vaccines Based on Allergen-Derived B Cell Epitopes. Immunol Lett (2017) 189:19–26. doi: 10.1016/j.imlet.2017.04.015 28472641 PMC6390931

[310] Shamji MH Kappen JH Akdis M Jensen-Jarolim E Knol EF Kleine-Tebbe J. Biomarkers for Monitoring Clinical Efficacy of Allergen Immunotherapy for Allergic Rhinoconjunctivitis and Allergic Asthma: An EAACI Position Paper. Allergy (2017) 72:1156–73. doi: 10.1111/all.13138 28152201

